# Identifying causal genetic variants for high-altitude adaptation through blood eQTL analysis in plateau populations

**DOI:** 10.1038/s42003-026-10840-6

**Published:** 2026-08-26

**Authors:** Chenghui Zhao, Jiawei Guan, Junhua Liu, Zhe Zhang, Mingxiang Zhu, Lei Fu, Anlin Dai, Kai Lin, Luo Zhang, Weidong Wang, Kunlun He, Jinlong Shi

**Affiliations:** 1 Medical Innovation Research Department of PLA General Hospital, Beijing, China; 2 Key Laboratory of Biomedical Engineering and Translational Medicine, Ministry of Industry and Information Technology, Chinese PLA General Hospital, Beijing, China; 3 National Engineering Research Center of Medical Big Data, Chinese PLA General Hospital, Beijing, China; 4 School of Computer Science and Technology, Dalian University of Technology, Dalian, Liaoning China

**Keywords:** Quantitative trait, Population genetics

## Abstract

A substantial number of genetic variants have been associated with high-altitude adaptation (HAA), yet most of them are located in non-coding genomic regions, leaving their specific functions and underlying mechanisms largely unknown. In this study, we analyze whole-genome and transcriptome sequencing data from a self-established cohort comprising 61 native highlanders (NHs) and 164 acclimatized newcomers (ANs), identifying 6,586 cis- and 34,203 trans-expression quantitative trait loci (eQTLs), along with 130 cell type-specific eQTLs. By further combining these data with a large East Asia (~30% Tibetan) genome-wide association study (GWAS) cohort, we employ colocalization and causal inference analyses to prioritize 85 cis-eQTLs associated with HAA and identify several novel candidate causal genes, including *EXOC8*, which is experimentally confirmed to regulate erythroid differentiation. Additionally, network analysis of these causal genes uncovers multiple regulatory pathways, mainly involving energy metabolism, autophagy, ubiquitination and inflammation. Our study offers a comprehensive eQTL map and reveals causal chains of “variant-gene-phenotype” for HAA-related traits, which provides new insights into potential regulatory mechanisms and targets for prevention and treatment of altitude sickness.

## Abstract (web summary)

A substantial number of genetic variants have been associated with high-altitude adaptation (HAA), yet their specific functions and underlying mechanisms remain largely unknown. In this study, the authors performed whole-genome and transcriptome sequencing of 61 native highlanders (NHs) and 164 acclimatized newcomers (ANs), yielding new insights into potential regulatory mechanisms and targets for prevention and treatment of altitude sickness.

## Introduction

High altitude promotes the formation of special and complex phenotypes. Tibetan-population has been regarded as an ideal subject for deciphering the phenotype-forming mechanisms of high-altitude adaptation (HAA), for their adaptive traits to high-altitude environments^1^. Over the past decade, genome-wide scans have uncovered numerous adaptive genetic variants (AGVs) and functional genes in Tibetans, such as *EPAS1* and *EGLN1*^2–6^. Despite the insights gained from current research into the mechanisms of adaptive genetic variations, several pivotal questions remain unresolved. To begin with, the challenge lies in the lack of large-scale transcriptome data suitable for HAA research, further analysis such as eQTL and causal inference, are unfeasible to establish the causal links between variants and phenotypes. Secondly, elucidating the mechanistic roles of those AGVs situated in non-coding genomic regions presents a formidable challenge. Lastly, known adaptive traits are the mixed results of response to plateau environment and ethnic characteristics, fine-grained integration analysis of multi-omics and phenotype is needed to clarify the different influencing mechanisms and action pathways for HAA.

Combined with causal inference methods such as transcriptome-wide association studies (TWAS), Mendelian randomization (MR), and genetic co-localization (coloc) analyses, eQTL analysis can identify potential causal variants and infer the regulatory link of “variant-gene-phenotype”. Among these, cis-eQTLs elucidate the proximal regulatory effects, while trans-eQTLs reveal the distal downstream genes and pathways influenced by genetic variants. It is essential to acknowledge the pronounced tissue and cell type specificity of eQTLs^7^. However, current research on eQTLs related to high-altitude adaptation has predominantly based on placental tissue^8^, with a notable absence of data from blood tissues. Given the pivotal role of blood physiological traits under hypoxic conditions, which encompasses hemoglobin concentration, red blood cell count, and nitric oxide (NO) levels^9^, the inclusion of blood tissue data is imperative. Moreover, blood physiological traits serve as a critical indicator of immune function changes.

**Fig. 1:**
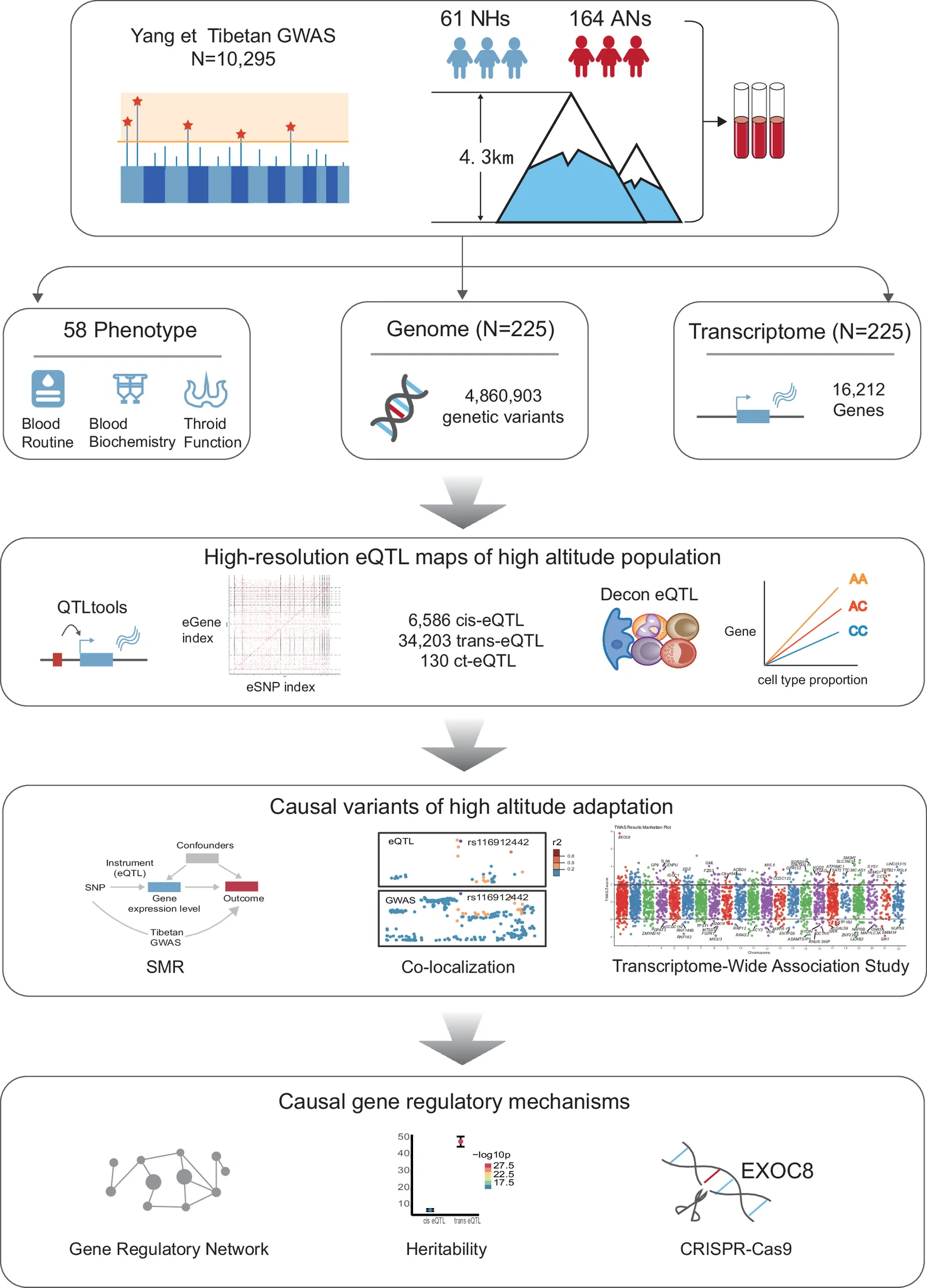
Graphic summary of datasets and analyses performed in the present study. This schematic illustrates the workflow of the study, including the data sources, preprocessing steps, analytical methods, and key outputs.

To delineate the genetic underpinnings of HAA phenotypes, pinpoint causal genes, and decipher regulatory mechanisms, we conducted a comprehensive study involving 225 human blood samples collected from the Tibetan Plateau, comprising 61 genetically homogeneous native highlanders (NHs; ≥3 generations without interethnic marriage) and 164 acclimatized newcomers (ANs) who are migrants of native lowlanders (Fig. 1 and Supplementary Table S1). We examined 58 blood physiological traits and performed whole genomic and transcriptomic sequencing, thereby constructing a comprehensive eQTL map for HAA and analyzing the differences in immune cell components between ANs and NHs populations. Utilizing causal inference techniques such as Mendelian randomization, colocalization, and TWAS, we prioritized potential causal variants and candidate regulatory genes linked to blood physiological traits associated with HAA. Notably, we experimentally validated *EXOC8* as an important functional gene for regulating erythroid differentiation. Ultimately, we elucidated the regulatory networks and pathway mechanisms of those causal genes, and categorized those genes into four distinct modules of energy metabolism, autophagy, ubiquitination, and inflammation. Our work provides valuable resources and promotes novel insights of HAA research.

## Results

### Unraveling the complex physiological adaptations to high-altitude environments

High-altitude adaptation is a complex phenotype that encompasses a range of physiological responses across various organs and tissues, with blood being one of the most critical systems involved^1^. To systematically investigate the physiological adaptations in NHs associated with chronic high-altitude exposure, we conducted a comprehensive comparative analysis of 58 phenotypic traits between ANs and NHs populations using the Mann-Whitney U test. We identified 28 distinct traits (*P*-value < 0.05) including body mass index (BMI), and 27 hematological traits such as hemoglobin (HGB), red blood cell count (RBC), hematocrit (HCT), eosinophils (Eos), creatinine (CREA), uric acid (UA), and free thyroxine (FT4), through routine blood tests, serum biochemistry, and thyroid function assessments (Supplementary Fig. S1 and Supplementary Table S2). This analysis offers a comprehensive understanding of the physiological mechanisms underlying high-altitude adaptation, highlighting the critical role of hematological traits in shaping the unique resilience of NHs populations to extreme environments.

### eQTL map of ANs-NHs population and functional characteristics

**Fig. 2:**
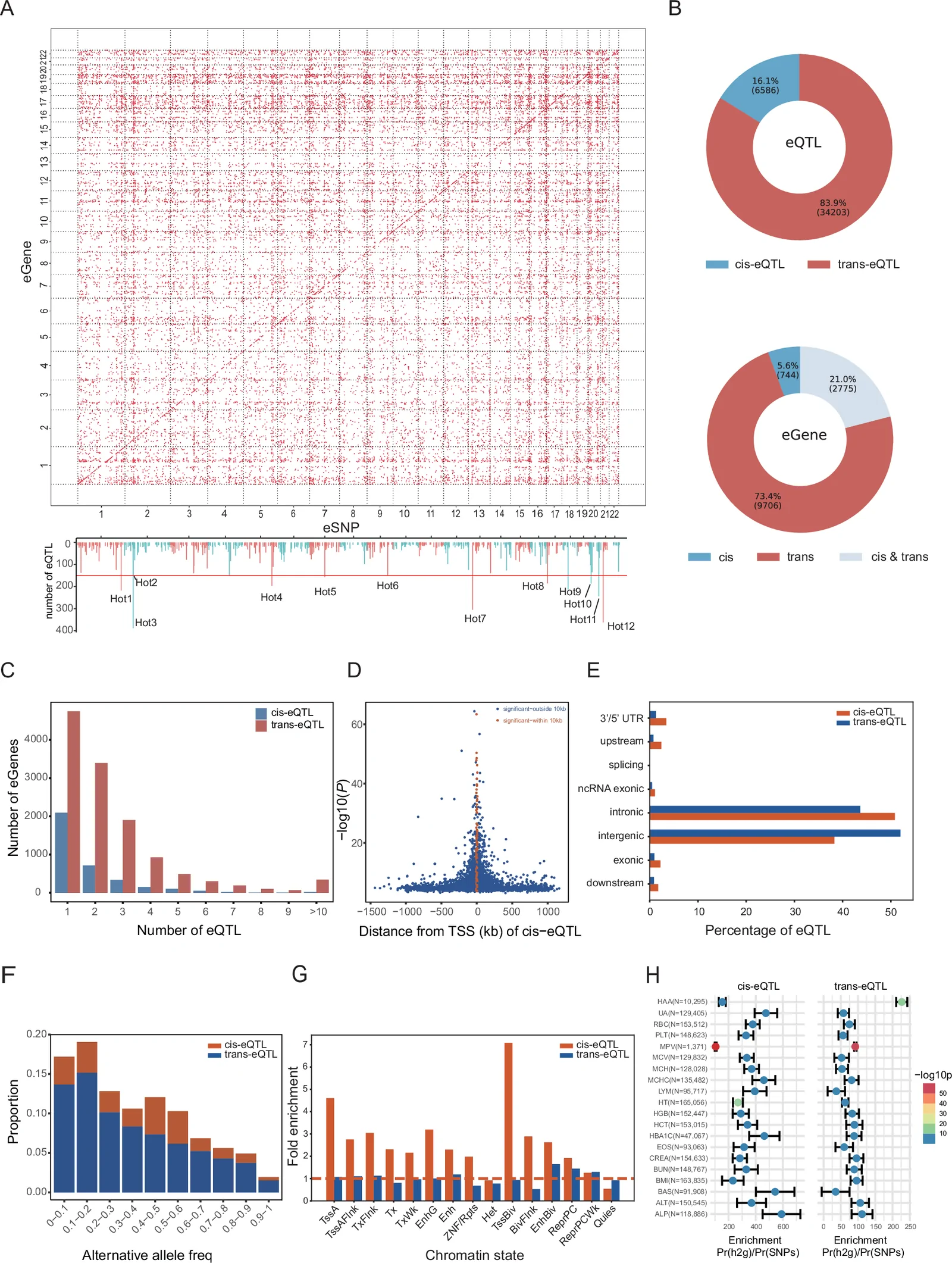
Characterizations of ANs-NHs eQTLs maps. **A** Scatterplot of 6,586 cis- (< 1 Mb, BH FDR < 0.05) and 34,203 trans- (>5 Mb, *P* < 1 × 10^−6^) high-confidence eQTLs (*x* axis), with expression of 13,225 eGenes (*y* axis), each dot represents a detected eQTL-eGene pair. The eQTL hotspots, where eSNPs at this locus regulate eGenes at distinct genomic locations, were annotated on the *x*-axis. **B** Pie charts showing the number and proportion of cis-/trans-eQTLs and eGenes. **C** Counts of eGenes regulated by different numbers of cis-/trans-eQTLs. **D** Distribution of eSNPs around the eGene TSS. **E** Distribution of cis- and trans-eQTLs on genomic functional regions. **F** Proportion of cis-/trans-eQTLs in each Alternative allele freq (AAF) interval. **G** Enrichment of eQTLs in chromatin state. TssA (Active TSS), TssAFlnk (Flanking active TSS), TxFlnk (Transcr. at gene 5’ and 3’), Tx (Strong transcription), TxWk (Weak transcription), EhnG (Genic enhancers), Ehn (Enhancers), Znf/Rpts (ZNF genes + repeats), Het (Heterochromatin), TssBiv (Bivalent/poised Tss), BivFlnk (Flanking bivalent Tss/Enh), EnhBiv (Bivalent enhancer), ReprPC (Repressed Polycomb), ReprPCWk (Weak repressed Polycomb), Quies (Quiescent/low). H LD score regression results showing heritability partitioned enrichments of cis-eQTL and trans-eQTL annotations across multiple high-altitude adaptation (HAA)-related trait GWAS. For the HAA phenotype, this analysis captures enrichment of high Fst signals rather than conventional heritability. Enrichments are colored by -log10(P), and error bars represent standard error (SE). UA urine acid, RBC Red blood Cell, PLT Platelet, MPV Mean Platelet Volume, MCV Mean Corpuscular Volume, MCH Mean Corpuscular Hemoglobin, MCHC Mean Corpuscular Hemoglobin Concentration, Lym Lymphocyte, HT Height, HGB Hemoglobin, HCT Hematocrit, HBA1C glycated hemoglobin, Eos Eosinophil, CREA creatinine, BUN blood urea nitrogen, BMI body mass index, Bas Basophil, ALT Alanine aminotransferase, ALP alkaline phosphatase.

Following rigorous quality control and normalization of genetic data and gene expression profiles from high-altitude populations, we obtained a dataset comprising 16,212 genes and 4,860,903 single nucleotide polymorphisms (SNPs). Principal component analysis (PCA) of genome and transcriptome distinctly segregated ANs and NHs populations (Supplementary Fig. S2A, S2B), a separation that is further corroborated by data from the 1000 Genomes Project (Supplementary Fig. S2C). Utilizing QTLtools, we identified 6586 cis-eQTLs with a false discovery rate (FDR) below 0.05, covering 3519 cis-eGenes and 5.943 cis-eSNPs, alongside 34,203 trans-eQTLs (*P* < 1e-6), encompassing 12,481 trans-eGenes and 17,457 trans-eSNPs (Fig. 2A, B). High-altitude eQTLs exhibited a non-uniform distribution, forming 12 distinct hotspots (Fig. 2A and Supplementary Table S3). The most prenominated hotspot spanned 836 kilobases (kb) on chromosome 2, ranging from 46,118,839 to 46,955,663 base pairs (bp). Notably, hotspot 1 and 3, located on chromosomes 1 and 2, respectively, overlapped with the *EGLN1* and *EPAS1* loci, genes known to be implicated in high-altitude adaptation. Most eGenes were influenced by one or two eQTLs (Fig. 2C), indicating that their expression variability is governed by a relatively straightforward genetic architecture. Cis-eQTLs were notably enriched within a 10 kb region proximal to transcription start site (TSS) (Fig. 2D), with the majority of eSNPs situated in non-coding regions such as introns and intergenic regions (Fig. 2E). As the frequency of the alternative allele increased, the proportion of eQTLs decreased (Fig. 2F); trans-regulation maintains stable effect, while cis-regulation prefers negative effect (Supplementary Fig. S3A). These observations suggest that the allelic variants specific to high-altitude populations exert distinct regulatory effects on gene expression.

In addition, we annotated cis- and trans-eSNPs leveraging the chromatin state data of K562 cell line, which is known as suitable for studying erythroid differentiation, a significant enrichment in regions of active TSS, promoters, enhancers, and within genes encoding zinc-finger proteins (ZNF) (Fig. 2G). These eSNPs were also enriched in histone marks such as H3K4me3, H3K9ac, and H3K27ac (Supplementary Fig. S3B). This enrichment pattern provides compelling evidence that eQTLs are located within regions critical for the regulation of gene expression^10^. Subsequently, we examined the enrichment of eSNPs in transcription factor (TF) binding sites and identified significant enrichment in 10 ANs-NHs differentially expressed TFs. Among these, *TFAP2A* interacts with *HIF-1*^11^, *USF2* plays a critical role in hypoxic transcriptional response^12^, and *STAT1* acts as a transcriptional suppressor of *HIF1A*^13^ (Supplementary Fig. S3C).

Recent studies suggest that the genetic basis of complex phenotypes is often enriched in conserved regions and enhancer^14^. Here, we explored the specific patterns of genetic variants influencing HAA phenotypes, which were different between ANs and NHs populations. GWAS summary of BBJ (blood physiological traits) and Yang’s East Asia population (Yang-GWAS, ~10 K EAS samples, ~30% are Tibetans, which regarded population specificity of Tibetan as one phenotypic indicator) were employed to evaluate the heritability and high Fst enrichment. We found traits controlled by HAA-related loci (such as HCT, HGB, RBC) undergone significant heritability enrichment of eQTLs. Notably, these eQTLs show significant enrichment of high Fst variants, indicating strong population-specific selection in NHs. Furthermore, compared with cis-eQTLs, trans-eQTLs demonstrate a substantially greater enrichment of high Fst variants linked to NHs HAA phenotypes, with an enrichment fold (Pr(H2g)/Pr(SNPs)) of 48.28 (Fig. 2H).

### The consistency and specificity of eQTLs between different populations

**Fig. 3:**
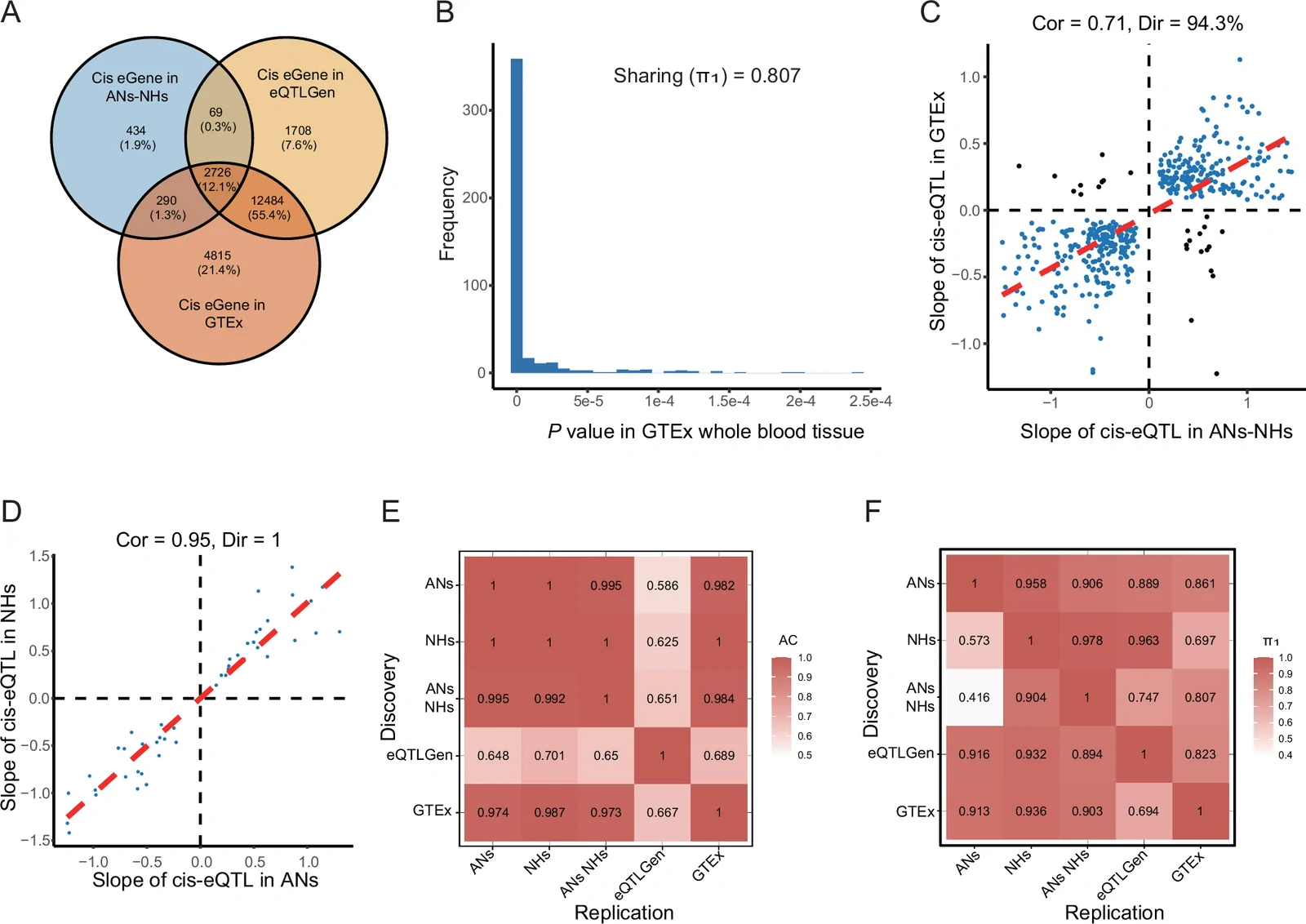
eQTLs consistency of ANs, NHs, ANs-NHs, GTEx, and eQTLGen. **A** Venn diagram of overlapping cis-eGenes among ANs-NHs, GTEx whole blood tissues and eQTLGen projects. **B** *P* value distribution of ANs-NHs eSNPs in GTEx whole blood tissues. **C** Comparison of scaled effect sizes from significant cis-eQTLs in ANs-NHs and in GTEx whole blood tissues (FDR < 0.05). ‘Cor’ is two-tailed Spearman’s rank correlation (*P* < 2e-16), ‘Dir’ denotes the proportion of variants sharing identical regulatory directions between two eQTL datasets. **D** Comparison of scaled effect sizes from significant cis-eQTLs in ANs and NHs. Overlapping eQTLs along with the AC **E** and π1 values **F** of cis-eQTLs between ANs, NHs, ANs-NHs, GTEx whole blood tissues, and eQTLGen projects.

We compared our ANs-NHs eQTL results with GTEx^15^ and eQTLGen^16^ using multiple statistics. First, we observed that 3085 (87.7%) of the 3519 cis-eGenes in our data overlapped with those in GTEx blood tissues and eQTLGen (Fig. 3A). However, there were only 4,600 (36.9%) trans-eGenes overlapped with eQTLGen (Supplementary Fig. S4A). Second, ANs-NHs eSNPs showed Storey’s π1 of 0.807 (Fig. 3B), with 94.3% demonstrating the same direction of effect as in GTEx blood tissues (Spearman correlation, 0.71, Fig. 3C). To shed light on the intricate regulatory mechanisms operating within NHs’ HAA, we executed a separate comparative eQTL analysis in both ANS and NHs cohorts, using both established and inferred covariates (detailed in Methods). We observed strong concordance in the regulatory direction of shared eSNPs between ANs and NHs populations (Spearman correlation, 0.95, Fig. 3D). Notably, 96% of ANs-shared and 98.5% of NHs-shared eSNPs with GTEx exhibited consistent regulatory effects (Spearman correlation = 0.74 and 0.77, respectively, Supplementary Fig. S4B, S4C). While both populations displayed perfect allelic concordance (AC = 1, Fig. 3E), ANs showed a lower Storey’s π1 (0.574, Fig. 3F) compared to NHs, indicating divergent regulatory architectures that may reflect population-specific adaptations to high-altitude environments. The eQTL results, derived from both the merged ANs-NHs population and each population independently, also demonstrated an impressively high level of congruence (AC > 99.2%, Fig. 3E).

We identified 10,032 trans-eGenes common to both populations and an additional 1,711 trans-eGenes that were specific to the NHs population (Supplementary Fig. S4D). These NHs-specific eGenes were enriched in pathways related to autophagy, mitochondrial gene expression, and mitochondrial autophagy, suggesting unique adaptation to the high-altitude environment (Supplementary Fig. S4E). Furthermore, a significantly NHs-specific trans-eSNP (rs72877497, Fst = 0.457) was found to regulate *FCGR2A*, a gene encoding a member of the immunoglobulin Fc receptor family^17^ (Supplementary Table S4).

### Cell type-specific eQTLs linked to HAA

Significant variations in the proportions of specific immune cells were found in the hematological profiling comparison between ANs and NHs populations (Supplementary Table S5 and Supplementary Fig. S5A, FDR < 0.05). Notably, the NHs cohort exhibited a higher proportion of T regulatory cells, coupled with a lower proportion of NK cells and naive B cells, which contribute to the mitigation of inflammatory reactions induced by the hypoxia conditions characteristics of high altitudes^18^. Besides, the most pronounced difference between ANs and NHs populations was noted in the granulocyte fraction (1.2%, FDR = 0.051). The disparity in immune function parameters is considered a pivotal aspect of the adaptive capabilities in high-altitude environments. We also observed varying correlations between predicted cell types (0.01 ≤ Pearson’s correlation (*R*) ≤ 1; Supplementary Fig. S5B).

Utilizing Decon-QTL, we identified 130 cis-ieQTLs (FDR < 0.05). Within these cell type ieQTLs, 12 ieGenes were recognized. The most substantial subset of interactions was observed with IgD+ IgM− B cells, comprising 89 ieQTLs (68.5%), which also represented the most divergent cell fraction (Supplementary Table S6). These ieQTLs provide valuable insights into cell type-specific genetic factors associated with HAA. For instance, the *HBD* gene, which encodes essential structural subunits of hemoglobin, were transcriptionally upregulated by the rs117543228 variant in HLA-DR+ regulatory T cells (FDR = 5.23e^−12^), suggesting a potential mechanism of high-altitude adaptation through enhanced hemoglobin synthesis and consequently improved systemic oxygen transport under chronic hypoxia. We further identified that rs201299572 upregulates *NCOA4* expression in HLA-DR+ regulatory T cells (FDR = 3.59e^−7^), which may support efficient erythropoiesis through modulating erythroid differentiation^19^, thereby potentially contributing to high-altitude adaptation.

### Shared genetic architecture of cis-eQTLs and HAA Traits

Summary-data-based Mendelian Randomization (SMR) techniques and colocalization analyses were used to rigorously assess the potential genetic pleiotropy between these cis-eQTLs and the spectrum of 20 HAA traits (19 differential hematological and physical traits in Supplementary Table S2, 1 derived from Yang-GWAS). Through a comprehensive SMR analysis, we identified 2141 cis-eQTLs significantly associated with 2020 genes (*P* < 0.05; Supplementary Table S7). Additionally, 2344 cis-eQTLs passed HEIDI’s multiple testing correction (*P* > 0.05). Notably, 824 out of the 2141 cis-eQTLs demonstrated colocalization between eQTL effects and GWAS results for the 20 HAA traits (PP4 > 0.7). This colocalization substantiates the shared causal SNPs supporting the forming of HAA traits through regulating gene expression, thereby provides compelling evidence for the genetic underpinnings of hypoxia-induced phenotypic variability.

**Fig. 4:**
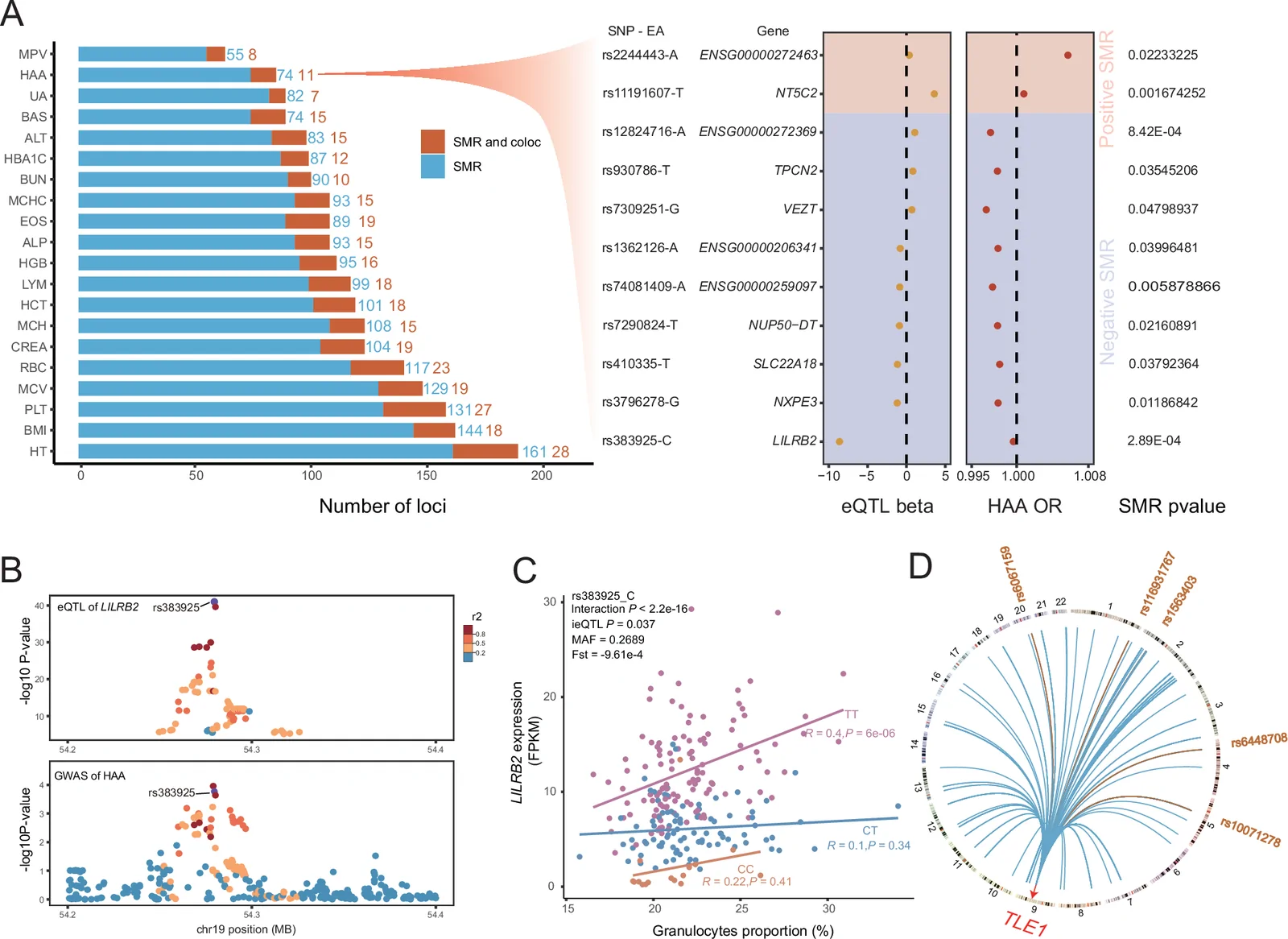
Co-localization and SMR analysis of HAA-related traits. **A** Numbers of significant eQTL loci in SMR (blue) and those in both SMR and co-localization (coloc; red) for 20 HAA-related traits. **B** Regional scatter plots of eQTLs for *LILRB2* (top) and GWAS for Yang-GWAS (bottom) in a 200-kb region centered on rs383925. Dot colors represent the linkage disequilibrium (measured by r^2^) of each variant with the causal variant (rs383925). **C** Cell-type ieQTL for *LILRB2* showing interactions with predicted granulocyte proportions. Each dot represents a sample. Colors indicate the SNP genotype, with yellow being the minor allele. Values under the alleles are the Pearson’s correlation coefficients and corresponding *P* value derived using a one-sided *F*-test; eQTL *P* values were derived using the standard normal distribution from meta-analyzed z-scores. FPKM, fragments per kilobase of exon per million fragments mapped. **D** Circos plot of autosomes indicating the cis- and trans-regulation of *TLE1*. Brown lines indicate regulation of *TLE1* by causal eSNPs.

Furthermore, we focused on Yang-GWAS and identified 136 cis-eSNPs associated with 145 cis-eGenes, all with statistical significance (*P* < 0.05). Among these, 11 cis-eSNPs showed evidence of colocalization (Fig. 4A), 2 of which had a positive SMR beta coefficient, indicating that increased gene expression is associated with better adaptation. Conversely, the remaining 9 cis-eSNPs indicated an inverse relationship with negative beta coefficients. We underscore two notable instances where MR and colocalization analyses converge on likely causal genes. Our examination revealed that elevated expression of *LILRB2* is associated with HAA (*P*_SMR_ = 2.89e-4, *P*_HEIDI_ = 0.7913). *LILRB2*, a member of the leukocyte immunoglobulin-like receptor family, transduces inhibitory signals that dampen immune response activation, thereby aiding in the modulation of inflammatory responses that are induced by hypoxia^20^. Additionally, the eQTL and GWAS signals colocalized with a posterior probability of 0.9274 (Fig. 4B). Intriguingly, ieQTL analyses indicated a positive interaction between increasing expression of *LILRB2* and rising proportions of granulocytes for the risk allele rs383925-C, with an interaction *P*-value of 2.2 × 10^−16^ (Fig. 4C). Furthermore, we identified the SNP rs930786 in proximity to *TPCN2*, which is associated with both *TPCN2* expression levels and HAA. *TPCN2* encodes a calcium-regulated ion channel situated on lysosomal membranes. This channel is implicated in the control of pigmentation by modulating melanosome pH and size, contributing to the adaptive mechanisms of NHs populations in response to high-intensity solar radiation^21^.

In our colocalization study, we identified a cohort of 704 genes associated with HAA (Supplementary Table S8). Under hypoxic stress, Notch1, in partnership with HIF-1α, orchestrates the regulation of hypoxia-responsive genes^22^. The mitochondrial proteins *OPA1* and *MRPL3* are instrumental in modulating energy metabolism, ensuring cellular bioenergetics are preserved amidst oxygen scarcity^23,24^. *GSTM4* counters the oxidative stress induced by hypoxia by detoxifying ROS^25^, and *PARP1* repairs DNA damage^26^, thereby protecting cells from damage. Hemoglobin subunits, as encoded by *HBG1* and *HBD*, and *VEGFA* are key drivers of angiogenesis^27^, which is critical for augmenting oxygen supply to tissues.

### Trans-eQTL elucidated the potential downstream effects of variants associated with HAA

Trans-eQTLs can identify the downstream consequences of trait-associated variants. Based on ANs-NHs populations, we identified 867 trans-eQTLs for 782 eGenes and 659 eSNPs of 20 HAA traits using SMR (Supplementary Table S9). As for Yang-GWAS, there were 286 trans-eQTLs consisting of 231 trans-eGenes and 181 trans-eSNPs. The high Fst enrichment of causal variants identified through SMR was discovered to be remarkably high, with a Pr(H2g)/Pr(SNPs) ratio of 6401.22 (Supplementary Fig. S6A), which illustrated that these potential causal variants were very important for HAA genetics basis.

Among these 181 potential trans-causal variants, 47 are adaptive genetic variants specific to NHs population (Fst > 0.2), 11 of which are situated in genomic regions that are well recognized as NHs-specific (Table 1), and 94 of which are situated in regulatory regions (Supplementary Table S10). Notably, eSNPs within *EPAS1* locus showed a significant correlation with the expression levels of *PURB*, which implicated in the regulation of hemopoiesis^28^ (Supplementary Fig. S6B). Additionally, eSNPs at *EGLN1* locus were found to be in association with the expression of *PDE8A*, a gene pivotal for cardiac function^29^ (Supplementary Fig. S6C). Furthermore, eSNPs within *CRIPT* locus demonstrated a robust association with genes implicated in the Toll-like receptor signaling pathway (*TLR1*), pulmonary hypertension regulation (*MAGED1*)^30^, and the regulation of cAMP-dependent protein kinase activity (*PKIG*) (Supplementary Fig. 6D). Among the potential trans-causal eGenes identified, 13 genes—*ZBTB16*, *TLE1*, *RCCD1*, *PURB*, *CKLF*, *MAGED1*, *AUTS2*, *TLR1*, *RELA*, *ERBB2*, *NEURL1, OPLAH* and *TLR6*—were found to be related to hypoxia (Supplementary Table S11). Of these genes, *NEURL1* is associated with BMI and *OPLAH* is associated with Bos and Eos. Importantly, *TLE1*, a transcriptional corepressor belonging to the TLE family, has the capacity to suppress NF-kappaB signaling^31^ and modulate HIF-1 signaling pathways and is regulated by majority of adaptive genetic variants identified in this study (Fig. 4D). In addition, all potential trans-causal eGenes were found to be correlated with HGB levels (Spearman correlation, *P* < 0.01; Supplementary Fig. S7A–M).

**Table1:** Causal variants of trans-eQTL

SNP	Fst	Base Gene	Gene Region	Target Gene	Function
rs1563403	0.45697	*CRIPT*	intergenic	*RNA5SP155*, *CKLF*, *TLE1*, *ARPC1B*, *MS4A6A*, *PKIG*, *RCBTB2*, *JAML*, *DBNDD1*	Notch signaling pathway (*TLE1*), Neutrophil Extravasation (*JAML*), Regulation Of cAMP-dependent Protein Kinase Activity (*PKIG*)
rs72797404	0.41077	*EPAS1*	intronic	*GLB1L*, *TTTY10*, *DGKK*, *DDX3X*, *ZFX*	Glycerolipid metabolism (*DGKK*), Positive Regulation of Protein Polyubiquitination (*DDX3X*)
rs117611189	0.39799	*CRIPT*	intergenic	*KATNBL1*, *MAGED1*, *AUTS2*, *CLEC7A*, *TLR1*	Toll-like receptor signaling pathway (*TLR1*), C-type lectin receptor signaling pathway (*CLEC7A*), Positive Regulation Of Interleukin-6 Production (*TLR1*, *CLEC7A*)
rs1562451	0.3955	*EPAS1*	intronic	*PURB*	Regulation Of Hemopoiesis (*PURB*)
rs7571218	0.39132	*EPAS1*	intronic	*RCCD1*, *CCDC93*	Endocytic Recycling (*CCDC93*)
rs6750698	0.37567	*RHOQ*	intronic	*ZNF322*, *EIF1AX*	RNA transport (*EIF1AX*), Regulation of Transcription by RNA Polymerase II (*ZNF322*)
rs12097901	0.34065	*EGLN1*	exonic	*PDE8A*	Cortisol synthesis and secretion (*PDE8A*)
rs2153364	0.33786	*EGLN1*	intergenic	*SFMBT2*	Negative Regulation of Gene Expression (*SFMBT2*)
rs2584462	0.31605	*ADH1C*	intergenic	*PRSS21*	Proteolysis (*PRSS21*)
rs11125089	0.26169	*SOCS5*	intergenic	*REM2*, *NPDC1*, *EIF1AY*	RNA transport (*EIF1AY*), Calcium Channel Regulator Activity (*REM2*)
rs61318674	0.24454	*ACTL8*	intergenic	*PRCC*, *MIR635*, *KHSRP*, *ZNF362*, *MAGED1*, *NAA10*	Negative Regulation Of Nitric Oxide Biosynthetic Process (*KHSRP*)

### TWAS reveals novel key genes associated with HAA traits and experimental validation

**Fig. 5:**
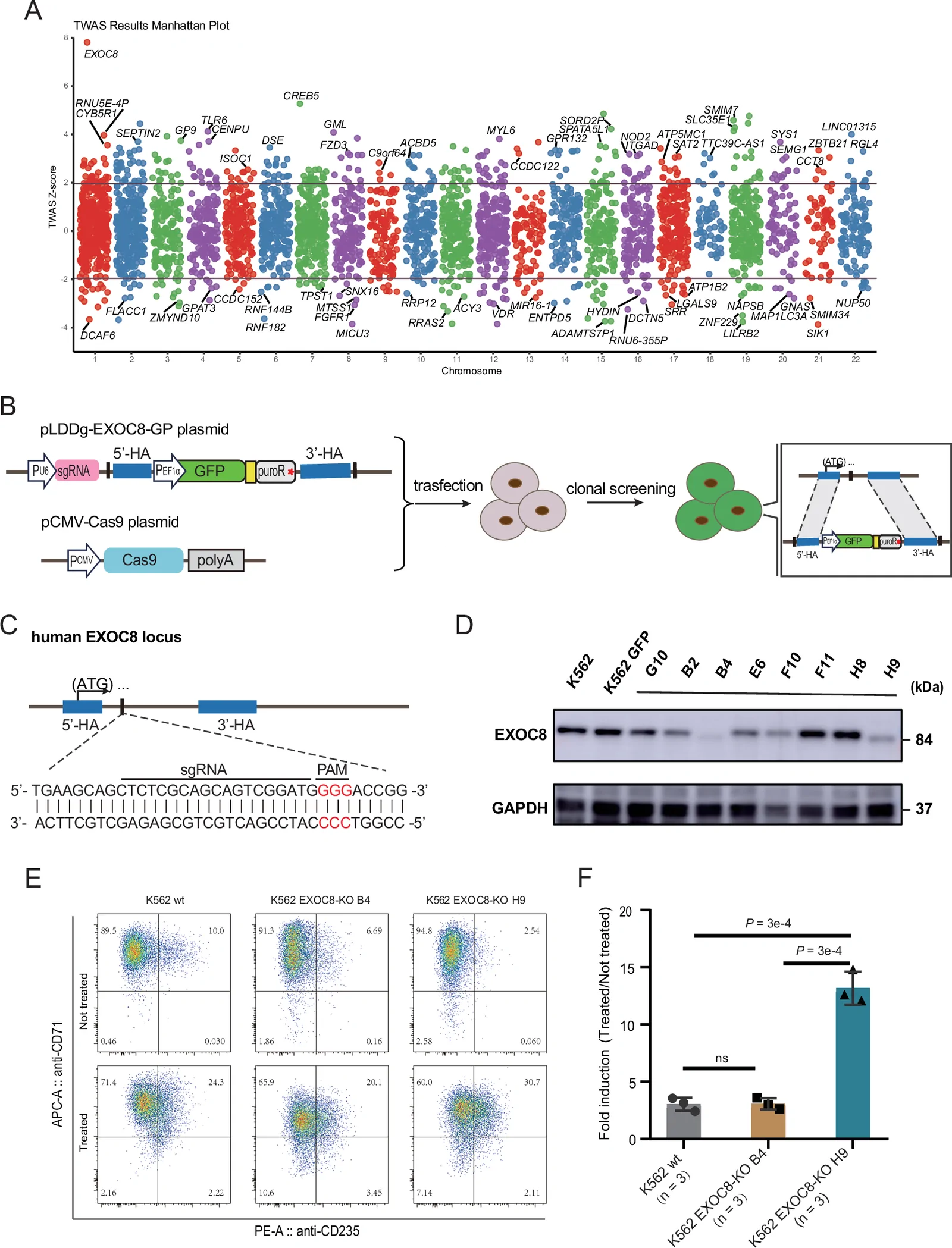
Transcriptome-wide association study for HAA and functional validation of EXOC8. **A** Manhattan plot of TWAS results. TWAS Z-scores (*y*-axis) are plotted against gene positions (*x*-axis) on each of the chromosomes. The gray lines indicate the Z-scores at the *P* value of 0.05, and genes exceeding this threshold are defined as TWAS significant genes in the following analysis. **B** The construction strategy of K562 EXOC8 knockout cells using CRISPR/Cas9 technology. **C** Diagram of the human EXOC8 locus. The sgRNA of EXOC8 target sequence and the protospacer adjacent motif (PAM) are indicated. Meanwhile, 5’HA and 3’HA refers to the locations of left and right homolougy arm, respectively. **D** Western blot analysis of the EXOC8 in different K562 EXOC8 knockout cell clones, where lane 1 is K562 widetype, lane 2 is K562 GFP as a mock control, lane 3–10 are K562 EXOC8-KO G10, B2, B4, E6, F10, F11, H8, and H9, respectively. And GADPH was used as a loading control. The uncropped and unedited Western blot images corresponding to this panel are provided in Supplementary Fig. S9F. **E** Flow cytometric analysis of erythroid differentiation in different K562 cell groups. Usually, the cell surface markers CD235a+/CD71+ double positive populations were used as the indicators of erythroid differentiation maturation. The gating strategy for the flow cytometry analysis is provided in Supplementary Fig. S9E. **F** Histogram analysis of erythroid differentiation across various K562 cell groups, with the fold induction of CD235a+/CD71+ populations induced by hemin and Ara-C treatment relative to the untreated control indicated on the vertical axis.

Given that high-altitude adaptive traits are predominantly associated with blood, we infer that blood eQTL data will provide a novel perspective for the dissection of HAA, especially considering the sensitivity of TWAS to tissue origins. We utilized Yang-GWAS^4^ and our blood eQTL dataset to identify genes whose estimated cis-regulatory expression is associated with high-altitude adaptive events. We identified 50 genes that were significantly associated with HAA by TWAS (FDR < 0.05; Fig. 5A, Supplementary Table S12). Among these, five novel HAA-related genes were discovered: *EXOC8*, *CREB5*, *MYL6*, *TLR6* and *DSE*. These five genes showed significant associations with HGB and RBC levels (Supplementary Fig. S8). Notably, *EXOC8* was further substantiated to be associated with HAA by both colocalization and fine-mapping analyses (Supplementary Fig. S9A, S9B), also showing significant differential expression between ANs and NHs populations. Our analytical results reveal that EXOC8 engages in protein-protein interactions with CDC42, INS, and EGFR. Notably, these three genes have been previously established as functional interactors of both HIF1A and EPAS1. Furthermore, CDC42 and INS are known to positively regulate erythropoiesis^32,33^, suggesting a plausible mechanistic pathway through which EXOC8 may influence erythropoiesis, potentially via modulation of CDC42 or INS activity (Supplementary Fig S9C). In a recent TWAS study, *EXOC8* was identified as significantly associated with red blood cell (RBC) traits, highlighting its potential role in erythropoiesis under hypoxic conditions^34^.

Here we hypothesis that reduced *EXOC8* expression may foster erythroid differentiation, considering *EXOC8* exhibits elevated expression levels in NHs relative to ANs individuals. To validate this, we initially generated a knockout (KO) model of *EXOC8* utilizing the CRISPR/Cas9 system. The construction strategy is shown in Fig. 5B, C and Methods. Different monoclonal cells were screened out and assessed by Western blotting analysis to identify human EXOC8 protein expression (Fig. 5D). The results showed that the EXOC8 protein in cell clones B4 and H9 was successfully knocked out, also labeled as K562 EXOC8 KO-B4/H9, respectively. Furthermore, we used common cell surface markers CD235a+/CD71+ double positive as the indicators of erythroid differentiation and maturation in our experiment. Flow cytometry analysis data demonstrated that after treatment with hemin and Arc-C, the fold induction of erythroid differentiation in the K562 *EXOC8* knockout cells was significantly higher than that in control groups (Fig. 5E, F and Supplementary Table S13), although the deletion of *EXOC8* gene can down-regulate the expression of CD235a/CD71 in K562 cells under traditional culture condition, when compared with wild-type K562 cells (Supplementary Fig. S9D). Together, these results suggest that the *EXOC8* gene is an important factor involved in and regulates erythroid differentiation, and knockdown of *EXOC8* significantly promotes the fold induction of erythroid differentiation of K562 cells, which is exactly consistent with our hypothesis.

### Network analyses of causal gene sets illustrate potential functional interactions and effects in HAA

Genes identified by SMR (*P* < 0.05), Coloc (PPH4 > 0.7), and TWAS (*P* < 0.05) are merged into a causal gene set (1,254 genes) for HAA (Supplementary Fig. S10 top). To further investigate the genetic basis of 23 differential blood and physical traits, we constructed causal gene set for each of these traits in the same way. 99 genes are shared with all 20 traits, and 539 genes are NHs-specific (Supplementary Fig. S10 down). The former provides a common genetic basis for those differential traits associated with HAA, meanwhile the later supports the phenotype-forming for NHs.

**Fig. 6:**
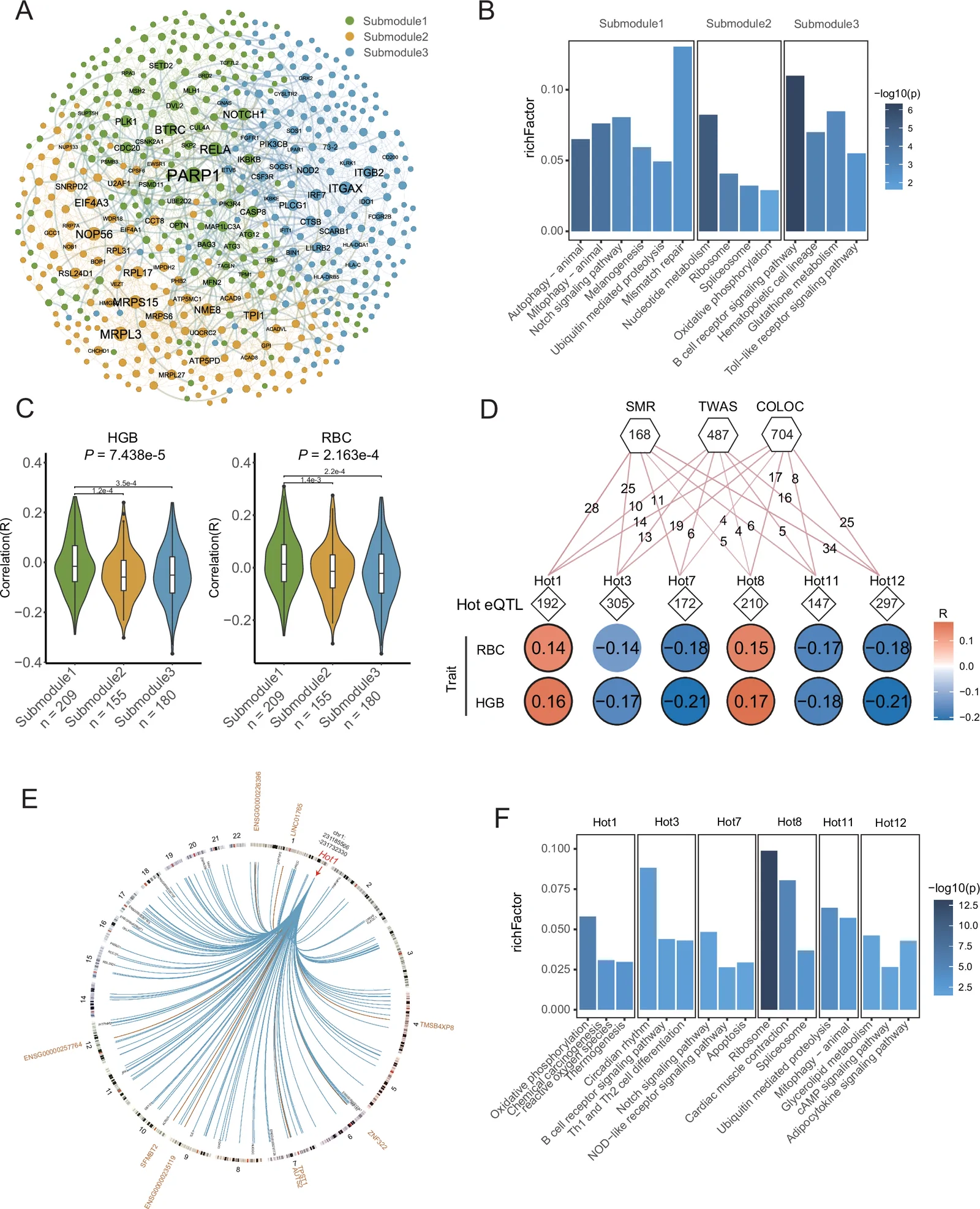
Network analysis and function enrichment of HAA causal genes. **A** PPI network of HAA causal genes. The genes with high degrees are labeled in the network. The network was divided into three submodules by Louvain algorithm. **B** Overrepresented KEGG terms for three submodules. **C** Correlation of causal genes in different submodules with HGB (right) and RBC (left). In each box plot, borders represent the first and third quartiles, center line denotes median, and whiskers extend to 1.5 times the interquartile range beyond the quartiles. **D** Identification of eQTL hotspots significantly associated with RBC and HGB. The two rows of the heatmap represent correlations between hotspots and HAA traits. The thickness of red solid line indicates the number of genes regulated by the eQTL hotspot in each causal gene sets. **E** Circos plot of autosome genes under cis- and trans-regulation by variants in Hot1 region. Brown lines indicate causal regulations. **F** Overrepresented KEGG terms for five hotspots.

To reveal the functional interactions of those causal genes (Supplementary Fig. S10 top), causal gene regulatory network was constructed based on protein-protein interactions (PPI). The network was divided into three submodules via Louvain algorithm (Fig. 6A and Supplementary Table S14), of which submodule1 (209 genes) was enriched in the KEGG pathway for autophagy, mitophagy and ubiquitin mediated proteolysis; submodule2 (155 genes) was relevant to nucleotide metabolism, ribosome, spliceosome and oxidative phosphorylation; and submodule3 (180 genes) was mainly enriched in B cell receptor signaling pathway, hematopoietic cell lineage, glutathione metabolism and Toll−like receptor signaling pathway (Fig. 6B and Supplementary Fig. S11). Notably, submodule1 contained many genes involved in immunity and inflammation, such as *BTRC*, *NOTCH1* and *RELA*. Submodule2 contained many genes involved in energy metabolism genes, such as *ATP5PD*, *ATP5MC1*, and *MRPL3*. Most genes in submodule2 and submodule3 exhibited negative correlations with both HGB and RBC levels, whereas those in submodule1 predominantly showed positive correlations with these hematological parameters (Fig. 6C).

Further, we explore the functional effects of forementioned 12 hotspots. Genetic networks through eQTL-eGene connections were constructed (Supplementary Fig. S12A). PCA analysis was carried out for each network, and the first PC was used to evaluate the correlation between hotspots and 23 differential blood and physical traits (Supplementary Fig. S12B). We found six hotspots were weakly correlated with HGB and RBC, respectively (Fig. 6D). The eGenes of those eQTLs on hotspots were also intersected with blood causal genes identified by SMR, Coloc, and TWAS. Hotspot1 (Hot1) regulated *SFMBT2*, *AUTS2*, *TPST1*, *ZNF322,* and et al. (Fig. 6E). Kyoto Encyclopedia of Genes and Genomes (KEGG) enrichment analysis revealed different functional terms for eGenes regulated by distinct eQTL hotspots (Fig. 6F). For example, genes regulated by Hot1 were enriched in oxidative phosphorylation and reactive oxygen species, whereas genes regulated by Hot7 were involved in Notch signaling pathway and NOD−like receptor signaling pathway.

## Discussion

Essentially, high-altitude adaptation is composed of organism’s response to high-altitude environment, and long-term evolutionary results of ethnic specificity. Regarding the former, existing studies have primarily focused on the acclimatization responses of lowlander populations after entering high-altitude environments. For the latter, a major focus has been on the special genetic characteristics of NHs population. In fact, both have complex genetic foundations, and formed various traits. In our study, based on fine-grained phenotypic indicators and whole-genome and transcriptome sequencing for plateau ANs-NHs populations, comprehensive eQTL and ieQTL maps are constructed to reveal the detailed characteristics at multi-omics levels. Further, causal inference, network analysis, and experimental validation are comprehensively utilized into the exploration of molecular mechanisms of HAA. Causal chains of “variant-gene-phenotype” were identified to interpret the functional roles of unknown non-coding genomic variants. To the best of our knowledge, our study provides novel insights into the underlying genetic basis and regulatory mechanisms associated with HAA.

### Mining potential target genes regulated by AGVs using an eQTL map

We constructed a high-altitude adaptation eQTL regulatory map encompassing 13,225 eGenes. Interestingly, we observed that trans-eQTLs exhibited higher heritability compared to cis-eQTLs, and majority of NHs-specific potential causal variants acting through trans-eQTLs. This suggests that AGVs in blood tissues are more likely to exert their effects through distal regulatory mechanisms. Furthermore, our study is the first to elucidate differences in blood cell composition between plateau ANs and NHs populations. NHs were found to have a higher proportion of granulocytes and T regulatory cells, which may enhance non-specific immunity and reduce inflammatory responses^35,36^. Additionally, we constructed cell-type eQTL maps, revealing molecular mechanisms underlying the differences in cellular composition, such as genes *HBD*, and *NCOA4*, which are significant for clarifying the mechanisms from genetic variation to gene expression, cellular composition, and phenotypic differences.

24 AGVs were identified to be the same as reported candidate AGVs^4–6^ (Supplementary Table S15), such as rs2437150 (Fst = 0.256) and rs1063321 (Fst = 0.177), respectively, which modulate the expression of *SPRTN* and *HLA-DQB1*, both reported by Zheng et al. These replicated signals reinforce the reliability of our dataset and provide crucial insights into the genetic architecture underlying high-altitude adaptation. Notably, the genetic variant rs3827760 (Fst = 0.29) demonstrated a significant correlation with the expression levels of three genes: *MALAT1*, *NFATC3*, and *ATG12*. *MALAT1*, recognized as a hypoxia-induced long non-coding RNA (lncRNA), has been shown to avert lung dysfunction in mice subjected to hypoxic conditions^37^. *NFATC3* is instrumental in the development of pulmonary hypertension under hypoxic stimuli^38^. *ATG12*, a component of the ATG12-ATG5-Atg16L conjugate, is known to play an important role in cellular autophagy triggered by hypoxia, engaging with the outer membrane of phagocytic vesicles to facilitate the expansion and maturation of autophagosomes^39^.

### Causal chains of “variant-gene-phenotype” elucidate the molecular mechanisms of HAA

HAA is a typical complex phenotype, with NHs often used as the most suitable subject to analyze the phenotype-forming mechanisms. NHs’ adaptive phenotypes include organism’s short-term responses to high-altitude environments and long-term evolving nature selection results. This study utilizes plateau ANs-NHs population to construct a comprehensive eQTL map and integrates Yang-GWAS to build candidate causal chains of “variant-gene-phenotype”. Our causal analysis indicates that classic high-altitude adaptation genes, such as *EGLN1* and *EPAS1*, may exert their effects through trans-eQTL mechanisms. *PURB*, identified as a trans-regulatory target gene of both *EPAS1* and *EGLN1*, plays a pivotal role in hematopoietic differentiation^40^ and may significantly influence erythropoiesis.

Excitingly, we identified 145 candidate causal chains via cis-eQTL, 11 of which were concurrently substantiated by Coloc validation. Among these findings, three chains emerged as especially noteworthy: “rs383925-*LILRB2*-HAA”, “rs930786-*TPCN2*-HAA”, and “rs11191607-*NT5C2*-HAA”. *LILRB2* expression showed a negative correlation with HAA, consistent with its elevated expression in immune responses during rat high-altitude pulmonary edema^41^. Similarly, *TPCN2* also exhibited a negative association, with implications for melanin pigmentation and protection against intense UV radiation in NHs populations^21^, as well as neuroprotection via autophagy under hypoxic conditions^42^. In contrast, *NT5C2* was positively associated with HAA, playing a pivotal role in purine metabolism, promoting the biosynthesis of urate salts^43^, alleviating hypoxia-induced hyperuricemia, and reducing the incidence of hyperuricemia. These findings underscore the utility of our causal chains in deciphering HAA mechanisms and highlight promising targets for functional validation.

### Potential mechanisms of HAA derived from causal genes

**Fig. 7:**
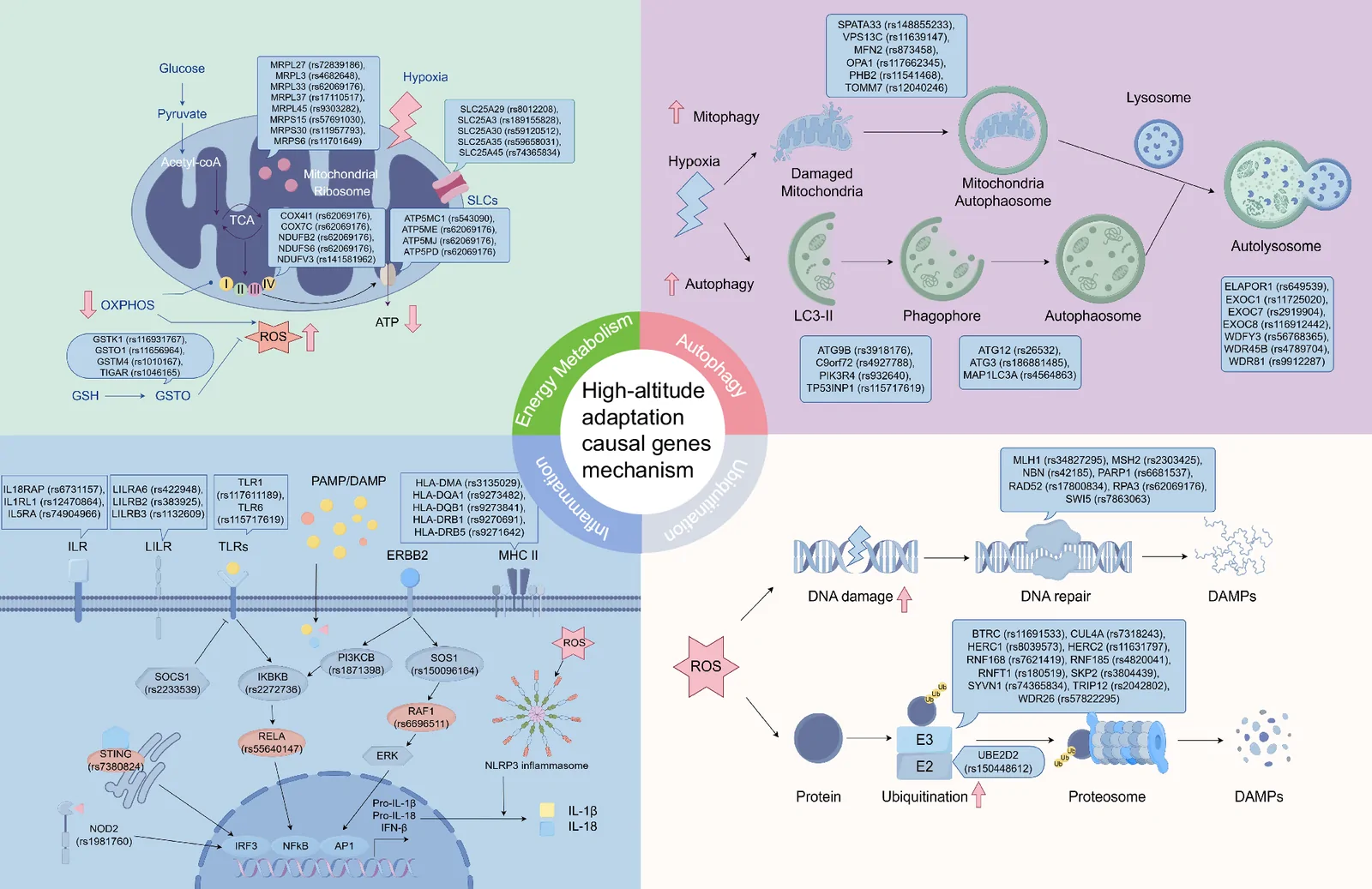
Potential mechanisms of causal genes for HAA. Four modules are enriched including energy metabolism, autophagy, ubiquitination and inflammation. OXPHOS oxidative phosphorylation, SLCs Solute Carrier Family 25, TCA tricarboxylic acid, ATP Adenosine triphosphate, ROS Reactive Oxygen Species, GSH glutathione, GSTO Glutathione transferase Omega, DAMP Damage-associated molecular patterns, PAMP Pathogen-associated molecular patterns.

Furthermore, we categorized candidate causal genes into four distinct modules: energy metabolism, autophagy, ubiquitination, and inflammation. In high-altitude environments, lowland individuals are primarily confronted with hypoxia, which poses a significant environmental challenge. Hypoxia disrupts mitochondrial energy metabolism and can inhibit oxidative phosphorylation^44,45^. This inhibition reduces the efficiency of the electron transport chain (ETC), leading to reduced ATP production and an increased reactive oxygen species (ROS) generation^46^. A hypoxic environment further induces mitochondrial and cellular autophagy, which serve to eliminate damaged organelles and maintain cellular functionality and integrity. However, excessive ROS and autophagy can cause DNA damage and ubiquitination-dependent protein degradation, leading to cell death and the release of damage-associated molecular patterns (DAMPs)^47^. DAMPs activate the STING-IRF3 pathway and, through Toll-like receptors (TLRs) and Nod-like receptors (NLRs), trigger the NF-κB pathway, resulting in the secretion of pro-inflammatory cytokines, including IL-1β and IL-18, which exacerbate tissue damage and disease^48^ (Fig. 7 and Supplementary Table S16).

After years of positive selective adaptation, NHs exhibit higher expression of genes related to mitochondrial energy metabolism compared to low-altitude individuals. These include genes involved in ETC, ATP production, mitochondrial ribosomes, and mitochondrial carrier family proteins. These highly expressed genes enable NHs to maintain efficient energy metabolism even under hypoxic conditions. Additionally, NHs exhibit increased expression of glutathione-related genes, which play a crucial role in antioxidant defense, counteracting the effects of excessive ROS. Our findings in blood align with the expression adaptations previously observed in NHs placentas.

We noted that higher expression of autophagy-related causal genes in ANs compared to NHs individuals, potentially due to the excessive autophagy response induced by high-altitude, leading to additional DNA damage and ubiquitination-dependent protein degradation. In contrast, NHs exhibits lower expression of genes associated with protein ubiquitination and DNA damage, but higher expression of DNA-repair genes, indicating better cellular homeostasis under hypoxic conditions.

Lastly, NHs show higher expression of immune-related genes and lower expression of inflammation-related genes, indicating a distinct advantage of their immune system in adapting to high-altitude environment. NHs produce fewer DAMPs, due to higher levels of TLRs, NLRs, interleukin receptors, and leukocyte immunoglobulin-like receptors. These factors enable a swifter response to DAMPs, preventing severe inflammatory responses, as evidenced by their lower NFκB levels. We conclude that the immune system of NHs populations is more sensitive in the high-altitude environments, effectively responding to hypoxia and other environmental stresses while minimizing unnecessary inflammatory reactions. This enhanced immune regulation capability likely helps reduce systemic inflammation levels, thereby decreases the risk of inflammation-related diseases in high-altitude environments. In summary, NHs have adapted to the high-altitude environment with enhanced energy metabolism, stabilized cellular states, and reduced inflammation, demonstrating stronger adaptive capabilities. We further demonstrated that module genes are correlated with the differential phenotypes associated with high-altitude adaptation (Supplementary Fig S13 and Table S17).

### Limitations of the study

This study still has several limitations. Firstly, although we utilized a highly homogeneous and valuable sample collection, the cohort size of NHs individuals is relatively small, with an imbalance between male and female samples. The smaller sample size of NHs reduces power to detect small-effect eQTLs and rare variants. Secondly, our study is subject to potential confounding from both population stratification and local LD structure. In addition to differences in high-altitude adaptation status, the AN and NH populations may have inherent differences in genetic background that are not fully captured even after PCA-based correction for population stratification. Moreover, although our causal analysis identified several loci with apparent trans-eQTL effects, the extensive LD in these regions means that we cannot exclude the possibility that the observed associations are driven by LD confounding rather than direct regulatory effects. Thirdly, while eQTL analysis elucidates the regulatory mechanisms underlying a subset of GWAS signals through gene expression effects, a substantial proportion of significant variants remain functionally uncharacterized. Comprehensive functional interpretation will require integration with additional multi-omics data. Lastly, due to research constraints, we were only able to experimentally validate the function of a single causal gene, *EXOC8*. The numerous causal genes identified in this study still require further experimental validation.

In conclusion, our study unravels the genetic foundation of HAA, particularly concerning blood-related physiological traits. It provides new insights into genetic regulation during the process of HAA and offers potential targets for the prevention and treatment of high altitude-related diseases.

## Method

### Sample collection and measurement of blood traits

In the Tibetan Plateau (4300 m), we collected 61 peripheral blood samples from native highland Tibetan Chinese (NHs) individuals with at least three generations of high-altitude ancestry and 164 samples from homogeneous acclimatized Han Chinese newcomers (ANs) matched for migration history, age, lifestyle, and over one year residence. All participants were informed about the study objectives and provided written informed consent prior to enrollment. This study was approved by the Ethics Review Board of the Chinese PLA General Hospital (S2018-298-02) and conducted in accordance with the Declaration of Helsinki. Routine blood tests were performed using the Mindray Auto Hematology Analyzer (BC-760), blood biochemical indicators were measured with the Mindray Automatic Biochemistry Analyzer (BS2000), and thyroid function was assessed using the Roche cobas 6000 Analyzer Series. All those tests were carried out on the same day of sampling.

### Library preparation and sequencing

DNA was extracted from peripheral blood using the TIANamp Blood DNA Kit, and WGS with a target coverage of 30×was performed on the DNBSEQ-T7 platform following MGI-provided protocols, with standard library preparation conducted in Shenzhen. RNA collected from samples in PAXgene tubes was isolated using the RNAprep Pure Hi-Blood Kit, TIANGEN, following the manufacturer’s protocol. rRNA was removed using the MGIEasy RNA Directional Library Preparation Kit, and a single- stranded cDNA library was prepared with the MGIEasy Circularization Kit. Whole-transcriptome sequencing was performed on the MGISEQ-2000 platform, with each sample sequenced to a depth of 20 G bases to ensure data quality. Quality control of the raw FASTQ files for both genomic and transcriptomic data was performed using SOAPnuke (v1.5.6)^49^.

### SNP identification

Variant calling was performed with Sentieon^50^. Briefly, reads were mapped to the reference genome (GRCh38) using BWA (v0.7.17)^51^. SAM files were sorted, indexed, and converted into BAM files. Duplicate reads were marked and removed. The BQSR (Base Quality Score Recalibration) algorithm was applied to calibrate base quality, and raw variants were called using the Unified Genotyper algorithm. VQSR (Variant Quality Score Recalibration) was used to filter variants according to six parameters: “QD < 2||FS > 60||MQ < 40||SOR > 3 || MQRanksum < −12.5 || ReadPosRanksum < −8.0”. For GWAS and eQTL analyses, SNPs with missing rate >2%, minimum allele count >3, minimum genotype quality > 30, minimum depth of coverage > 3, minor allele frequency (MAF) < 0.05 and Hardy-Weinberg equilibrium (HWE) value *P* < 10-4 were removed using VCFtools (v0.1.13)^52^ with the parameters (-maf 0.05, -max-missing 0.98, -mac 3, -minQ 30, -minDP 3, -hwe 0.0001). A total of 4.86 million SNPs were retained for downstream analyses.

### Expression profiling

Clean reads were mapped to the human reference genome (GRCh38) using Hisat2 (v2.2.0)^53^. Gene expression levels were quantified using featureCounts (v2.0.2)^54^ based on Gencode v37 comprehensive gene annotations. Differential gene expression was evaluated by the Wilcoxon test. Genes with a |log fold change| > 1 and *P*-values < 0.05 were considered differentially expressed. For eQTL analysis, only genes with FPKM > 1 in more than 5% of samples were considered expressed. A total of 16,213 genes were retained for downstream analyses.

### eQTL analysis

QTLtools (v1.3.1)^55^ was executed in permutation pass mode with 1000 permutations to identified the optimal nominal SNP associated with each trait. Cis-eQTLs were defined as regulatory loci within a 1-megabase (Mb) interval upstream and downstream of the gene start site regardless strand, and were filtered using the Benjamini–Hochberg FDR < 0.05 according to GTEx v8 cis-QTL mapping pipeline^56^. Trans-eQTL, defined as regulatory loci either on different chromosomes or more than 5 Mb away from the gene start site on the same chromosome, were selected with a *P*-value < 1 × 10^−6^ according to eQTLGen Consortium^16^. We included age and sex as covariates to account for their effects on gene expression, the top 5 genotyping principal components (PCs) to control for population stratification, and 5 probabilistic estimation of expression residuals (PEERs)^57^ to mitigate the impact of batch effects and other unknown technical variables. To reduce eQTL redundancy, LD pruning was performed using plink (v1.9)^58^ with an LD threshold (*R*^2^ > 0.2) in a 500 kb radius. Genes associated with at least one significant eQTL signal were designated as eGenes, and their corresponding variants were termed eSNPs.

Cell type-dependent eQTL effects in bulk RNA-seq data were identified using Decon2 framework^59^. First, Decon-cell predicted proportions of 34 immune cell subpopulations from bulk RNA-seq data using elastic net regression (*α* = 0.5, *λ* selected via 10-fold cross-validation), trained on FACS-quantified cell counts from the 500FG cohort^60^. Next, Decon-eQTL (v1.4) integrated whole blood expression profiles, genotypes, and predicted cell proportions through a multivariate linear model that simultaneously tests genotype-by-cell-type interaction effects for six major immune populations (granulocytes, CD14+ monocytes, CD4+/CD8+ T cells, B cells, NK cells). Significant ieQTLs were identified using the threshold of FDR < 0.05.

Cis- and trans-eQTLs were selected using a *P-*value < 1e-6 threshold for hotspots identification, with analyses performed using hot_scan (v2) with parameters (-m 100,000 -s 0.05)^61^. Cis- and trans-eGenes within key hotspots were visualized in Cytoscape (v3.10.2).

### eQTL consistency

We employed AC and π1^62^ to assess the concordance of eQTL effects across different population datasets, including ANs-NHs, NHs, ANs, GTEx, and eQTLGen. When performing consistency comparisons between datasets, we typically designate one dataset as the discovery set, while the remaining datasets serve as validation sets, based on which relevant consistency metrics are calculated. AC, expected to be 50% for randomly distributed eQTL effects, quantifies the proportion of significantly correlated effects that exhibit a consistent directional agreement between discovery and replication datasets. π1 estimates the true positive rate of eQTL effects in the replication cohort, independent of effect direction but potentially influenced by sample size. To calculate AC, we identified the SNP-gene pairs with the most significant p-values in the discovery dataset and compared with those in the replication cohort, considering only pairs significant in both. The percentage of pairs with the same allelic effect direction was then calculated. For π1, the most significant SNP-gene pairs from the discovery dataset were matched by *p*-value order in the replication cohort, excluding untested combinations. The proportion of true null *p*-values (π0) was estimated using the pi0est function in *R*, and π1 was derived as 1 - π0.

### Functional enrichment of eQTLs in epigenetic markers and transcription factors

To test functional enrichment of eQTLs, we annotated lead cis-eQTL SNPs using data from Annovar^63^ (for example, intron and exon), chromatin immunoprecipitation sequencing (ChIP -seq) peaks for 7 histone modifications (H3K4me1, H3K4me3, H3K9ac, H3K9me3, H3K27ac, H3K27me3 and H3K36me3), 15 chromatin states from the K562 leukemia cell line (E123)provided by the Roadmap Epigenomics Mapping Consortium (REMC)^64^, 78 consensus transcription factors^65^ and DNA-binding protein binding sites from ENCODE DNase-seq^66^. We focused on 11 binding proteins that showed differential expression in blood between ANs and NHs populations. Specifically, cis-eQTL SNPs were annotated to their respective functional intervals based on genomic positions, and the proportion within each category was calculated. To eliminate bias, an equivalent number of cis-SNPs were randomly sampled from all variation sites as “control SNPs”. This sampling procedure was repeated 1000 times, and the enrichment score for each functional interval was calculated as the ratio of the proportion of eQTL SNPs to the mean proportion of control SNPs in that interval across the 1000 replicates.

### Partitioned heritability

Partitioned heritability was measured using stratified LD score regression (S-LDSC) (v1.0.1)^14^ to identify heritability enrichment of complex traits among functional features, including cis-eQTLs, trans-eQTLs, and SNPs corresponding to SMR. To quantify the heritability explained by these SNPs, we constructed cis-eQTL, trans-eQTL, and SMR binary annotations based on all corresponding SNPs. LD scores for the annotated SNPs were calculated using a 1 cM LD window and the 1000 Genomes Project Phase 3 East Asian (EAS) population as the LD reference panel^67^. Enrichment for each annotation was calculated as the proportion of heritability explained by each annotation divided by the proportion on SNPs in the corresponding annotation category. Cis-eQTLs, trans-eQTLs, and SMR-related SNPs were tested for enrichment in genetic variants with differential traits (UA, RBC, PLT, MPV, MCV, MCHC, MCH, LYM, HT, HGB, HCT, HBA1C, EOS, CREA, BUN, BMI, BAS, ALT, ALP). The GWAS data for differential traits in the Japanese population were downloaded from https://pheweb.jp/pheno. In the case of the Yang GWAS, LDSC was applied beyond its conventional complex trait heritability framework, and the apparent eQTL heritability enrichment in fact reflected enrichment of high Fst eQTL signals.

### Mendelian randomization

Summary-data Bases Mendelian Randomization (SMR) (v1.3.1)^68^ employs a Mendelian Randomization framework to determine whether the impact of a SNP on the phenotype is mediated by gene expression. To preserve expression changes attributable to population-specific genetic effects in NHs, cis- and trans-eQTLs adjusted for age as the sole covariate, along with NHs GWAS data, were utilized as inputs to prioritize genes associated with high-altitude adaptation. However, Mendelian randomization analyses for other physiological phenotypes utilized eQTL results adjusted for all covariates. Significance was defined as SMR *P* < 0.05 and HEIDI test *P* > 0.05.

### Colocalization

Bayesian co-localization analysis was implemented using R package coloc (v.5.2.3)^69^ to estimate the posterior probability of association between Yang-GWAS variants and cis-eQTLs. Cis-eQTLs were partitioned by gene and matched with GWAS data based on corresponding SNPs. Posterior probability >0.7 was considered evidence of co-localization.

### TWAS and fine-mapping

We conducted TWAS for high-altitude adaptation using the FUSION package (v3)^70^ with custom SNP-expression weights generated from blood tissue. Age, sex, the first five genotyping principal components (PCs), and five probabilistic estimation of expression residuals (PEERs) were employed as covariates. For each gene, SNPs within 500 kb of its start or end site were selected to estimate cis-SNP heritability using the GCTA-REML algorithm. We utilized five expression prediction models (best cis-eQTL, LASSO regression, Elastic-net regression, Bayesian sparse linear mixed model (BSLMM), and best linear unbiased predictor) to calculate predictive weights for genes with significant cis-SNP heritability (nominal *P* value < 0.05), and identified the model with the highest R^2^ from five-fold cross-validation as the optimal model. The constructed predictive model was combined with Yang-GWAS for TWAS analysis, where *P* values were calculated and adjusted for FDR. Genes with a TWAS *P* value < 0.05 were considered significant.

To reduce the impact of LD and predictive weights from TWAS on the statistical analysis of gene-trait associations within TWAS risk regions, and to control for pleiotropic effects, we utilized FOCUS (v0.7)^71^ for statistical fine-mapping to prioritize candidate susceptibility genes. We leveraged a comprehensive multi-tissue, multi-eQTL reference panel weight database by integrating GTEx v7 weights from PrediXcan with weights provided in FUSION. This database includes data from several tissues, such as adipose, peripheral blood, whole blood, and brain, creating a unified and functional database for FOCUS.

### GO and KEGG enrichment

We employed ClusterProfiler (version 4.6.0)^72^ to perform functional enrichment analyses, including Gene Ontology (GO) and KEGG pathways, for eQTL hotspot genes and causal genes. The results were stringently filtered to identify significant enrichments, with threshold (FDR < 0.05), ensuring robust and statistically meaningful associations.

### Construction of GRNs

We integrated 1464 causal genes identified from SMR, Coloc, and TWAS analyses. Utilizing the STRING database^73^, we constructed a gene regulatory network (GRN) comprising 573 causal genes. The GRN was divided into three sub-modules based on gene expression connectivity using the Louvain algorithm implemented in R-igraph (version 1.4.0). The network graph was visualized using Gephi software (version 0.10.1).

### Cell lines and cell culture

The human leukemic cell line K562 cells (RRID: CVCL_0004) for erythropoiesis studies was obtained from the American Type Culture Collection (ATCC) and widely used as cell model of erythropoiesis. In this study, we used K562 cells and K562 *EXOC8* knockout cells (K562 *EXOC8* KO cells). K562 *EXOC8* KO cells were constructed using CRISPR/Cas9 technology. Briefly, an sgRNA sequence targeting the human *EXOC8* gene (5’-GCTCTCGCAGCAGTCGGATG-3’) was designed using the CHOPCHOP web tool (https://chopchop.cbu.uib.no/). An *EXOC8* knockout plasmid was constructed as a dual-component vector containing a GFP-tagged homology-directed repair (HDR) template flanked by two cut-site sequences that are analogous to sgRNA targeting for *EXOC8* gene and a U6 promoter-driven sgRNA cassette. K562 cells were co-transfected with the *EXOC8* knockout plasmid (Addgene ID: #254957) and the pCMV-Cas9 plasmid (Addgene ID: #254958) using the jetPRIME reagent (Cat. No. 101000046, Polyplus) according to the manufacturer’s instructions. The mass ratios of *EXOC8* knockout plasmid to pCMV-Cas9 plasmid for transfection were 4:1.After one week, the K562 *EXOC8* KO cells were selected as monoclonal populations for continued culture. These cells were cultured in RPMI 1640 medium (Cat. No. C11875500BT, Corning) supplemented with 10% fetal bovine serum (Cat. No. FND500, Excell) and 1% penicillin/streptomycin (Cat. No. 15140-122, Gibco) and maintained at 37 °C in a 5% CO_2_ incubator. All the above cells were tested and confirmed to be free from mycoplasma contamination. All sequences used in this study are provided in Supplementary Table S18.

### Induction of erythroid differentiation

K562 cells were induced to undergo erythroid differentiation using 40 μM hemin(cas: # 16009-13-5, Solarbio) and 0.1 ng/ml cytosine arabinoside'(CAS: #IC0630, Solarbio)^74^. K562 cells were seeded at a density of 2 × 105 cells per well in six-well plate and cultured either with or without the inducing agents mentioned above. After 72 h, the cells were gently collected and replaced with fresh erythroid differentiation medium. After 120 h of induction culture, cell pellets from each group were collected for subsequent erythroid differentiation analysis.

### Western blotting analysis

First, different groups of K562 cells were lysed in RIPA buffer, and protein concentrations were determined by the BCA method. Secondly, 20 μg of total protein from each lysate was loaded and separated by electrophoresis on 10% sodium dodecyl sulfate (SDS) polyacrylamide gels. Proteins were then transferred to polyvinylidene difluoride membranes (Bio-Rad) and blocked overnight. Furthermore, the membranes were incubated with an anti-Exo84 antibody (H-1) (Cat. No. sc-515532, Santa Cruz Biotechnology, 1:1000) and anti-GAPDH antibody (Cat. No. GB15002, Servicebio, 1:3000) overnight at 4 °C. The following day, the membrane was then washed and incubated with HRP-conjugated goat anti-mouse IgG (H + L) secondary antibody (Cat. No. GB23301, Servicebio, 1:5000). After membrane washing with Tris-buffered saline containing 0.05% Tween-20 (TBST), specific bands were developed using the enhanced chemiluminescence (ECL) method and detected with a ChemiDoc imaging system(Bio-Rad).

### Flow cytometry analysis

We used cell surface markers CD71 and CD235a to identify erythroid differentiation. K562 cells from different groups were collected by centrifugation, resuspended in PBS, and added with 2 μL of CD71-APC antibody (clone CY1G4, Cat. No. 334108, BioLegend, 1:100) and 2 μL of CD235a-PE antibody(clone HI264, Cat. No. 349106, BioLegend, 1:100). Antibodies were added at 2 μL per 1 × 10⁶ cells in 100 μL of staining buffer. Following incubation, cells were washed twice with PBS and analyzed in PBS using a flow cytometer (BD FACS Canto II). The FCS files were analyzed using FlowJo software (BD Biosciences). Gating strategies are illustrated in Supplementary Fig. S9E.

### Statistics and reproducibility

Statistical analyses were performed using R (v4.3.1). Comparisons between two independent groups were performed using an unpaired Student’s *t*-test. For comparisons involving three groups, non-parametric analyses were performed using the Kruskal-Wallis test. For cell-based experiments, each replicate was initiated from distinct cryopreserved tubes derived form different cell batches. Each group included three independently cell samples.

### Reporting summary

Further information on research design is available in the Nature Portfolio Reporting Summary linked to this article.

## Supplementary information

Transparent Peer Review file

Supplementary Fig. S1-13

Description of Additional Supplementary Files

Supplementary Data

Supplementary Table S1-18

Reporting Summary

## Data Availability

All datasets are available on request or through online repositories. The raw Genotype data and RNA-seq data have been deposited in the National Genomics Data Center (NGDC) under the accession number HRA009343, and the eQTL summary data are available at the same repository with the accession number OMIX014471. The Yang-GWAS summary statistics are available for download from https://cnsgenomics.com/data/yang_et_al_2017_pnas.html or Zenodo^75^. East Asian high-altitude adaptation phenotype GWAS summary statistics are available at BBJ (https://pheweb.jp/). The statistics of eQTLs in GTEx v8 are available at https://www.gtexportal.org/home/downloads/adult-gtex/qtl. The statistics of eQTLs in eQTLGen are available at https://www.eqtlgen.org/phase1.html. Other data sources used in this study include 15 chromatin states from REMC (https://egg2.wustl.edu/roadmap/data/byFileType/chromhmmSegmentations/ChmmModels/coreMarks/jointModel/final/); the histone modifications Chip-seq data for K562 cell line (https://egg2.wustl.edu/roadmap/data/byFileType/alignments/consolidated/); the DNA-binding protein binding sites from ENCODE DNase-seq (https://www.encodeproject.org/annotations/ENCSR695MET/); 1000genome (http://ftp.1000genomes.ebi.ac.uk/vol1/ftp/data_collections/1000_genomes_project/release/20181203_biallelic_SNV/); ihypoxia annotation (http://ihypoxia.omicsbio.info/index.php).

## Code availability

Code is available at Gitee (https://gitee.com/jinlongshi/eQTL_HAA.git) and Zenodo^75^.
