# Supplementary material for: Identifying causal genetic variants for high-altitude adaptation through blood eQTL analysis in plateau populations
Source: Commun Biol. 2026 Aug 26;9:1186. doi: 10.1038/s42003-026-10840-6 (PMC13558674; doi:10.1038/s42003-026-10840-6)
Supplement: Supplementary file 1 [file 42003_2026_10840_MOESM1_ESM.pdf]

nature portfolio

Peer Review File

Identifying Causal Genetic Variants for High-Altitude Adaptation through Blood eQTL Analysis in plateau Populations

Corresponding Author: Dr Jinlong Shi

This file contains all reviewer reports in order by version, followed by all author rebuttals in order by version.

Version 0:

Reviewer comments:

Reviewer #1

(Remarks to the Author)
This manuscript presents a comprehensive analysis of blood expression quantitative trait loci (eQTLs) in plateau Han-Tibetan populations to identify causal genetic variants underlying high-altitude adaptation (HAA). The integration of whole-genome sequencing, transcriptome profiling, and causal inference methods (e.g., SMR, colocalization, TWAS) represents a rigorous approach to dissect the "variant-gene-phenotype" chain in HAA. The identification of novel causal genes (e.g., EXOC8) and functional validation of its role in erythroid differentiation are significant strengths. However, several critical issues need to be addressed to enhance the robustness, clarity, and impact of the study, including sample size limitations, statistical methodology details, functional validation depth, and mechanistic interpretation.

I have some major concerns as well as minor comments for Authors' consideration to revise the manuscript.

Major concerns

1. Sample Size and Population Stratification
The Tibetan cohort size is relatively small (59 individuals) compared to Han (166), which may limit the power to detect Tibetan-specific eQTLs. This imbalance could introduce bias in population comparison analyses (e.g., cell type proportion differences, ieQTL identification).
The authors may like to supplement with sensitivity analyses to assess how sample size affects key results (e.g., power calculations for eQTL detection, bootstrap resampling to validate population-specific signals). Discuss limitations of the small Tibetan sample size in interpreting "Tibetan-specific" findings.

2. Statistical Methodology and Reproducibility
1) eQTL Analysis: The manuscript mentions using QTLtools with permutations but lacks details on how covariates (age, sex, PCs, PEER factors) were selected and their impact on eQTL detection. The rationale for choosing FDR < 0.05 for cis-eQTLs and P < 1e-6 for trans-eQTLs should be justified.
2) Causal Inference: The criteria for colocalization (PP4 > 0.7) and SMR (P < 0.05, HEIDI P > 0.05) are standard, but the manuscript does not address potential pleiotropy or LD confounding, especially for trans-eQTLs. For example, how were LD-pruned SNPs selected to avoid spurious associations?
3) Cell Type Deconvolution: The method for estimating immune cell proportions (Decon-cell) and identifying ieQTLs (Decon-QTL) requires more detail, including validation of cell type estimates against flow cytometry data (if available) and assessment of deconvolution accuracy.
The authors may like to expand the Methods section to include detailed parameter choices, covariate selection rationale, and validation of statistical approaches. Provide supplementary tables with full eQTL and causal inference results (e.g., all cis-eQTLs with FDR, colocalization PP values for top hits).

3. Functional Validation and Mechanistic Depth
While EXOC8 is validated, the manuscript identifies 1,254 causal genes, but only one is experimentally tested. This limits the generalizability of functional conclusions. The mechanism linking EXOC8 to erythroid differentiation is not explored (e.g., downstream pathways, interaction with known HAA genes like EPAS1/EGLN1).

The authors may like to either expand functional validation to 1-2 additional key genes (e.g., LILRB2, TPCN2) or provide in silico functional evidence (e.g., ChIP-seq binding of HIF-1α at EXOC8 locus, protein-protein interactions with erythroid differentiation factors) to strengthen mechanistic links.

4. Interpretation of "Causal" Signals

1) Trans-eQTL Hotspots: The manuscript highlights trans-eQTLs as major contributors to HAA heritability but does not address why trans-eQTLs (typically more variable across populations) show stronger enrichment than cis-eQTLs. Are trans-eQTLs more likely to be population-specific due to regulatory divergence?
2) Overlap with Known HAA Genes: Classic HAA genes (EPAS1, EGLN1) are linked to trans-eQTLs regulating CPSF6, PURB, etc., but the biological relevance of these target genes to hypoxia adaptation (e.g., CPSF6 in mRNA polyadenylation under hypoxia) is underdeveloped.
The authors may like to expand Discussion to interpret trans-eQTL enrichment in the context of HAA evolution (e.g., trans-regulatory networks as adaptive "modulators" of hypoxia responses). Link target genes of EPAS1/EGLN1 trans-eQTLs to known hypoxia pathways (e.g., HIF signaling, erythropoiesis).

5. Clarity of Results and Figures
1. Figure 2H: The heritability enrichment plot is difficult to interpret. Labels for traits (e.g., "ALP") are unclear, and the rationale for selecting these traits (vs. key HAA traits like HGB, RBC) should be explained.
2. Figure 7: The proposed mechanism model combines four modules but lacks direct links to genetic variants (e.g., which SNPs regulate genes in each module). Adding examples of causal SNPs/gene pairs (e.g., rs383925-LILRB2 in inflammation) would strengthen the model.
The authors may like to revise figures to improve readability (e.g., clearer trait labels, color-coding for modules) and add insets explaining key interactions. Supplement with a table mapping top causal SNPs to their respective modules and traits.

Minor Issues
1. Differential Expression Analysis: The manuscript mentions 27 hematological traits differing between Han and Tibetan but does not link these to differential gene expression (e.g., are genes in the energy metabolism module upregulated in Tibetans?).
2. References: Some key HAA studies (e.g., 2023 Zheng et al. on Tibetan genome sequencing) are cited but not integrated into discussions of AGV overlap.
The authors may like to add a supplementary figure correlating differential traits with module gene expression. Update discussions to contextualize findings with recent HAA literature.

Reviewer #2

(Remarks to the Author)
Zhao et al. presented a paper entitled “Identifying Causal Genetic Variants for High-Altitude Adaptation through Blood eQTL Analysis in plateau Han-Tibetan Populations”, in which the authors constructed a valuable data source of eQTLs for the Han-Tibetan Populations. A comprehensive and robust set of eQTLs derived from a large Han-Tibetan cohort is highly valuable for genetic studies on high-altitude adaptation (HAA), as it bridges genetic loci with their target genes. Based on 225 plateau Han-Tibetan subjects enrolled in this study, the authors identified a substantial number of cis- and trans-eQTLs and inferred causal eQTLs associated with HAA. Overall, this study provides an important new eQTL resource for HAA research, with solid data and thorough analysis. I only have a few minor concerns, outlined below.

The description of the discovery and replication datasets used for eQTL consistency analysis is unclear. The authors have briefly described the datasets for eQTL consistency analysis in the Methods section, but the description is simple and with little details. The authors should briefly state the basic ideas of the dataset design in the corresponding Results section and describe the details clearly in the Methods section.

Considering the association between EXOC8 and HAA is critical for this paper, some key figures in Supplementary Fig S9 should be displayed in Figure 5. Meanwhile, The authors should consider reducing the large space taken by Figure 5B.

The phenotype of 58 in Figure 1 is misleading as the corresponding numbers for Genome and Transcriptome is the numbers of subjects in the cohort but not the number of features.

Full name of chromatin states should be stated in the legend of Figure 2G.

Typos: in Figure 2D, a space is missing between from and TSS; in Figure 2E, “od” should be “of”.

All the proteins should be in standard form not italic form. Line 206: “Under hypoxic stress, Notch1, in partnership with HIF-1α, orchestrates the regulation of ...”. The Notch1 should be NOTCH1 if the protein of this gene is indicated.

Line 246: A space is missing in “TLR6and DSE”.

Reviewer #3

(Remarks to the Author)
Zhao et al. report an eQTL study of 225 plateau Han-Tibetan blood samples. Using this resource, they investigate the genetic pathways underlying high-altitude adaptation. While the signature of recent adaptation has been detected in many genomic loci across human populations previously, only a few have been understood at the level of causal chains. This manuscript has the potential to be of great interest to the field, but significant concerns (listed below) need to be addressed.

Line 27: The authors claim to have "identified several novel causal genes". Only causal gene with strong statistical or experimental evidence is EXOC8.

Line 31: They claim their study "reveals causal chain of variant-gene-phenotype for HAA". There is no direct evidence that the phenotype is the target of selection for HAA. It should be toned down as "reveals a causal chain of variant-gene-phenotype for an HAA-related trait."

Lines 73-78: Mann-Whitney test was used here to test if there exists a significant difference in the phenotypic distributions between Hans and Tibetans. First, it is not very clear what is being done here without digging into Table S2. Second, how is this analysis relevant for HAA? It does not necessarily tell that these trait differences are genetic, let alone driven by HAA.

Lines 78-86: Exactly how GSMR was used here? It is not very clear. How does this lead to the claim that these traits are "causally associated with Tibetan high-altitude adaptation"? There are no details in the Methods section about how they selected SNPs or by which criteria they chose outcome traits. More importantly, why is Tibetan (is this Yang-GWAS?) an exposure, and why are the physiological traits outcomes here? Shouldn't the test be the other way around? Isn't it more likely that larger effects for physiological traits cause stronger selection (i.e., greater allele frequency differences in Yang-GWAS)?

Lines 120-124 (Figure 2H): Yang-GWAS is not exactly a GWAS here. What is the measured phenotype, and how is the heritability defined? It is a binary phenotype, but it is hard to see how there could be an underlying liability distribution for Tibetanness, like for hypertension. In addition, authors need to ensure that the population stratification is not driving the high enrichment. Note that eQTL (especially, trans-eQTL) can be inflated by stratification, and Yang-GWAS signals are also stronger in highly divergent SNPs between populations.

Line 158: What is the corresponding FDR? If the cutoff here ("$P < 0.05$") is the nominal P value, it would be too lenient, especially for trans-QTL.

Line 176: This is not "causal inference." Neither SMR, TWAS, nor Coloc has the specificity to establish a causality. It only tests for the shared causal effects between traits, which is far from causality.

Lines 220-239: To suggest "trans-causal variants," the trans-eQTL signals should be fine-mapped down to a SNP level. Are these 181 trans-causal variants fine-mapped? Further, positive selection drives Fst up for linked SNPs in flanking regions by LD (genetic hitchhiking). This effect is more extensive if the adaptation is relatively recent. The authors presented no evidence here to support that the same causal variants drive both trans-eQTL and HAA.

Lines 337-339 ("We identified 145 novel causal chains..."): This is an overstatement.

Data availability: It appears that genome-wide summary statistics are not freely available without restriction for eQTL at https://ngdc.cncb.ac.cn.

Version 1:

Reviewer comments:

Reviewer #1

(Remarks to the Author)
All comments addressed satisfactorily.

Reviewer #2

(Remarks to the Author)
The authors have addressed all my concerns, and I have no more question.

Reviewer #3

(Remarks to the Author)
The manuscript has been greatly improved, but I believe there are remaining issues with reviewer #3 points.

Point #4: I do not agree that the current GSMR analysis indeed serves as "further causal validation." First of all, I cannot see exactly what the authors mean by "high-altitude adaptation leads to specific phenotypic outcome" in an evolutionary or statistical genetic model. Especially, in the light that the phenotypic outcomes (regression betas) are based on the modern Japanese population from BBJ. Second, MR is valid only under the assumption that the instrument variable (SNPs) must only affect the outcome through the exposure, not through any other pathway. If they insist HAA has to be the exposure, I

don't think the authors can simply claim that SNPs affect outcomes (say WT) only through HAA. HAA is essentially Fst here, and it is not a measure of the selective benefit the SNP provides for high altitude adaptation. It's the overall fitness and can be low if it leads to other deleterious consequences, on top of adaptive benefit. Third, nominal p-values will not do here. It should be Bonferroni corrected. If this is not the area of expertise, perhaps this analysis should be dropped and the claim toned down regarding the target of selection. The paper would still be interesting to the readership.

Point #5: In that case, the authors should not call it "heritability" or "heritability enrichment" because they are clearly not. To avoid misleading, I would suggest changing the term to something like "enrichment of high Fst" and clearly stating that the method (ldsc) is used outside the conventional context since Yang-GWAS is not based on a complex trait (it doesn't matter whether it's binary or quantitative).

Authors should mention the limitations of confounding due to stratification in the Discussion section.

Point #6: FDR is 1.0 for most of the signals in Table S7. Analyses based on these signals are not acceptable.

Point #7: There are still other places overstating the reliability of causality tests.

Lines 49-51: Combined with causal inference methods such as transcriptome-wide association studies (TWAS), Mendelian randomization (MR), and genetic co-localization (coloc) analyses, eQTL analysis can identify authentic causal variants and establish causal link of “variant-gene-phenotype”.

Lines 65-67: Utilizing causal inference techniques such as Mendelian randomization, colocalization, and TWAS, we identified the causal variants and regulatory genes linked to blood physiological traits associated with HAA.

Point #8: I think the authors are misunderstanding my point on LD confounding. LD pruning can't address this question. Actually, LD pruning makes false positives more likely. I was suggesting a Credible SNP analysis for eQTL data. However, note that you cannot run credible SNP analyses on Yang GWAS since this is not really a GWAS. That said, perhaps in some cases, you may be able to find some compelling cases (without LD pruning), suggesting the shared causal variant is very convincing between traits.

Point #10: I believe that the standard practice of resource paper is to release all cis-eQTL *genome-wide* regardless of their p-values at least. I understand this can be too onerous for trans-eQTLs.

Version 2:

Reviewer comments:

Reviewer #3

(Remarks to the Author)
Authors have addressed all points.

Open Access This Peer Review File is licensed under a Creative Commons Attribution 4.0 International License, which permits use, sharing, adaptation, distribution and reproduction in any medium or format, as long as you give appropriate credit to the original author(s) and the source, provide a link to the Creative Commons license, and indicate if changes were made.
In cases where reviewers are anonymous, credit should be given to 'Anonymous Referee' and the source.
The images or other third party material in this Peer Review File are included in the article's Creative Commons license, unless indicated otherwise in a credit line to the material. If material is not included in the article's Creative Commons license and your intended use is not permitted by statutory regulation or exceeds the permitted use, you will need to obtain permission directly from the copyright holder.
To view a copy of this license, visit https://creativecommons.org/licenses/by/4.0/

Dear Dr. Ani Manichaikul,

We sincerely thank you and reviewers for your thorough evaluation of our manuscript and providing constructive feedback. We greatly appreciate the time and effort invested in reviewing our work. Following the reviewers' suggestions, we have carefully revised the manuscript and made the necessary modifications to address each comment in detail.

In the revised manuscript, all changes within the manuscript are highlighted in red for clarity. Notably, to better reflect the characteristics of the study populations—namely Tibetans with long-term high-altitude adaptation and Han individuals with short-term high-altitude exposure—we have unified the terminology throughout the manuscript, referring to them as Native Highlanders (NHs) and Acclimatized Newcomers (ANs), respectively.

Below, we provide point-by-point responses to each reviewer's comment, along with supplementary results and detailed explanations of the revisions made.

COMMENTS TO THE AUTHOR

Reply to Reviewer #1

We sincerely thank you for the time and effort you devoted to reviewing our manuscript and for your encouraging comments on its merits. We also greatly appreciate your clear and detailed feedback, and we hope that the additional analyses and results provided in this revision adequately address all of your concerns.

Notably, to better capture the characteristics of the study populations—Tibetans with long-term high-altitude adaptation and Han individuals with short-term high-altitude exposure—we have standardized the terminology throughout the manuscript, referring to them as *Native Highlanders (NHs)* and *Acclimatized Newcomers (ANs)*, respectively. In the following responses, for ease of reading, we have used the notations *Tibetan (NHs)* and *Han (ANs)* consistently. Thank you.

Major concerns

1. Sample Size and Population Stratification

Reviewer point #1:

The Tibetan cohort size is relatively small (59 individuals) compared to Han (166), which may limit the power to detect Tibetan-specific eQTLs. This imbalance could introduce bias in population comparison analyses (e.g., cell type proportion differences, ieQTL identification). The authors may like to supplement with sensitivity analyses to assess how sample size affects key results (e.g., power calculations for eQTL detection, bootstrap resampling to validate

population-specific signals). Discuss limitations of the small Tibetan sample size in interpreting "Tibetan-specific" findings.

Author response #1:

We sincerely appreciate the reviewer's insightful comments regarding the sample size imbalance between Tibetan(NHs) and Han(ANs) populations. We have corrected the sample-size error of Tibetan(NHs) (n=61) and Han(ANs) (n=164) in Line 61. We fully acknowledge this limitation and have conducted additional analyses to address the concerns, as detailed below:

1. Statistical Power Assessment

We performed power calculations for eQTL detection using the PowereQTL[1]. For Tibetan(NHs) samples (n=61), our study achieves 80% power to detect eQTLs with MAF >0.0794 (Fig 1a), which covers most of the reported significant eQTLs (98.04%) in our study (Fig 1b). For comparison, the Han(ANs) cohort (n=166) achieves 80% power for MAF > 0.0751 under the same conditions (Fig 1c), which covers 95.31% significant eQTLs (Fig 1d). Although the Tibetan(NHs) cohort has a relatively small sample size, under equivalent statistical power requirements, its minor allele frequency (MAF) values show negligible differences compared to the Han(ANs) population.

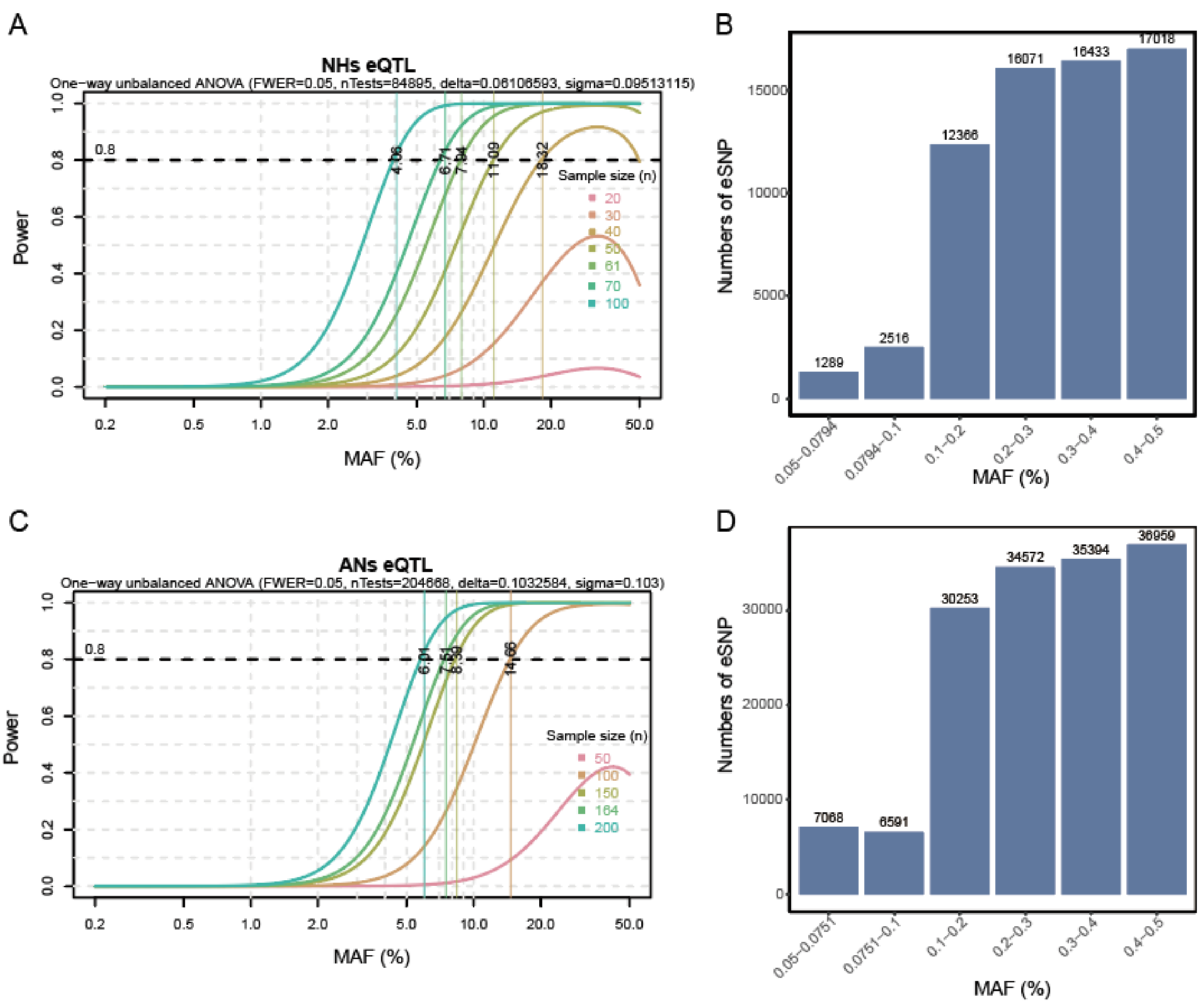

Figure 1 (A) The statistical power of NHs eQTL analysis as a function of the minor allele frequency (MAF) with different sample sizes. (B) Bar plot showing the distribution of eSNPs in NHs across different MAF bins. (C) The statistical power of ANs eQTL analysis as a function of the MAF with different sample sizes. (D) Bar plot showing the distribution of eSNPs in ANs across different MAF bins.

Ref:

1. Dong, X. et al. powerEQTL: an R package and shiny application for sample size and power calculation of bulk tissue and single-cell eQTL analysis. Bioinformatics 37, 4269-4271 (2021).

2. Bootstrap Resampling Validation

As suggested, we performed bootstrap resampling by drawing 49 individuals (80% of the 61 total samples) with replacement for 200 iterations to evaluate the stability of Tibetan(NHs)-specific eQTLs. In 91.5% of bootstrap samples, 92% eQTLs of were replicated in our reported Tibetan(NHs)-specific eQTLs, indicating robust signals despite the smaller sample size (Fig 2).

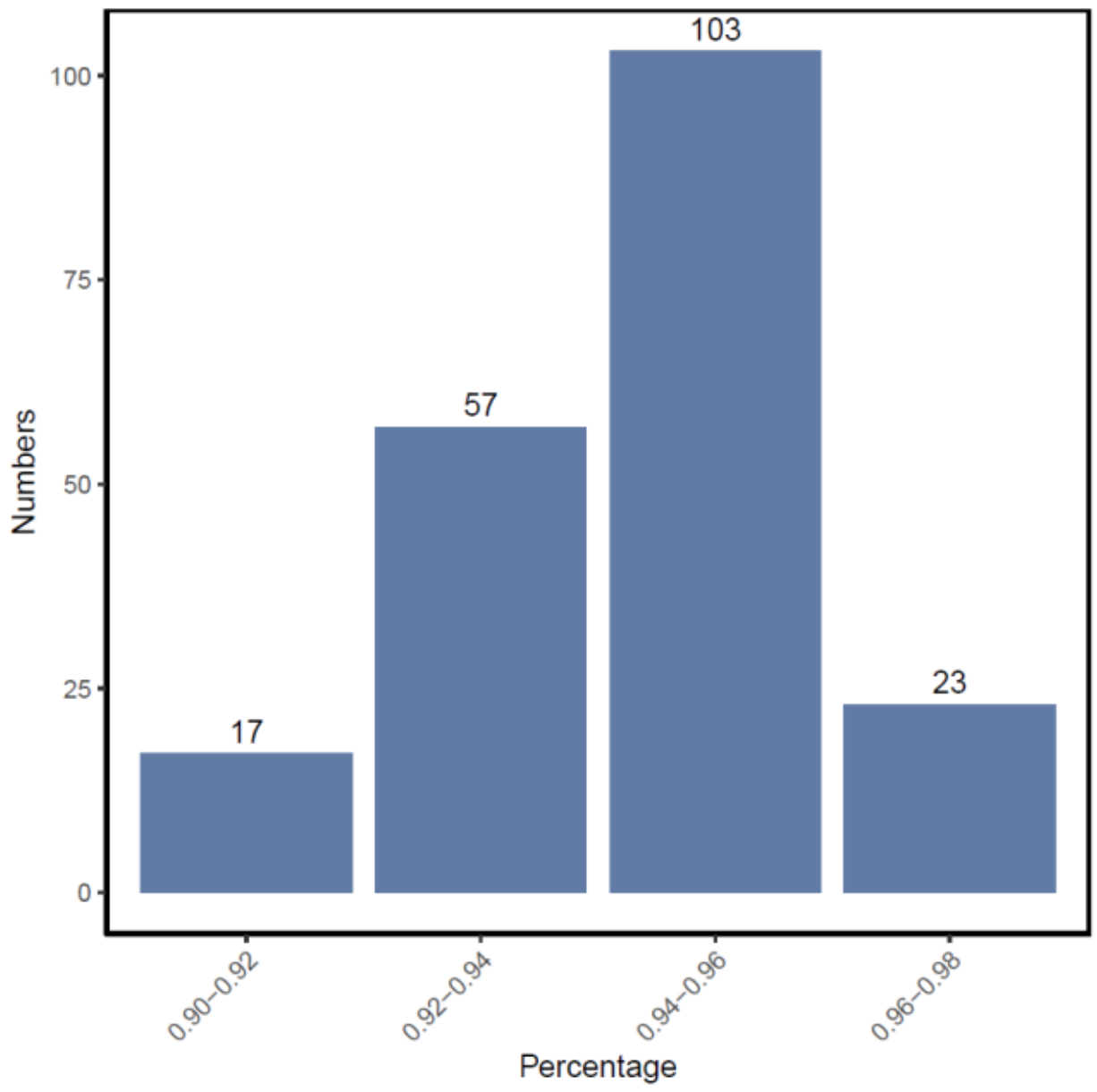

Figure 2. Bar plot showing the number of bootstrap resamplings at each overlap percentage.

3. Limitations Discussion

We added limitations discussion in Line 403-404.

Added Line 403-404: The smaller sample size of Tibetan(NHs) reduces power to detect small-effect eQTLs and rare variants.

4. Cell type proportion differences

To exclude potential bias arising from imbalanced sample sizes in the cell-type composition analysis, we first repeated the analysis with a balanced subset comprising 61 Han(ANs) and 61

Tibetan(NHs) individuals. Most results remained unchanged except for CD24+ CD38+, Transitional B cells and Monocytes (CD14+). Thus, the observed differences in cell-type proportions are minimally affected by sample-size imbalance (Fig3A, B).

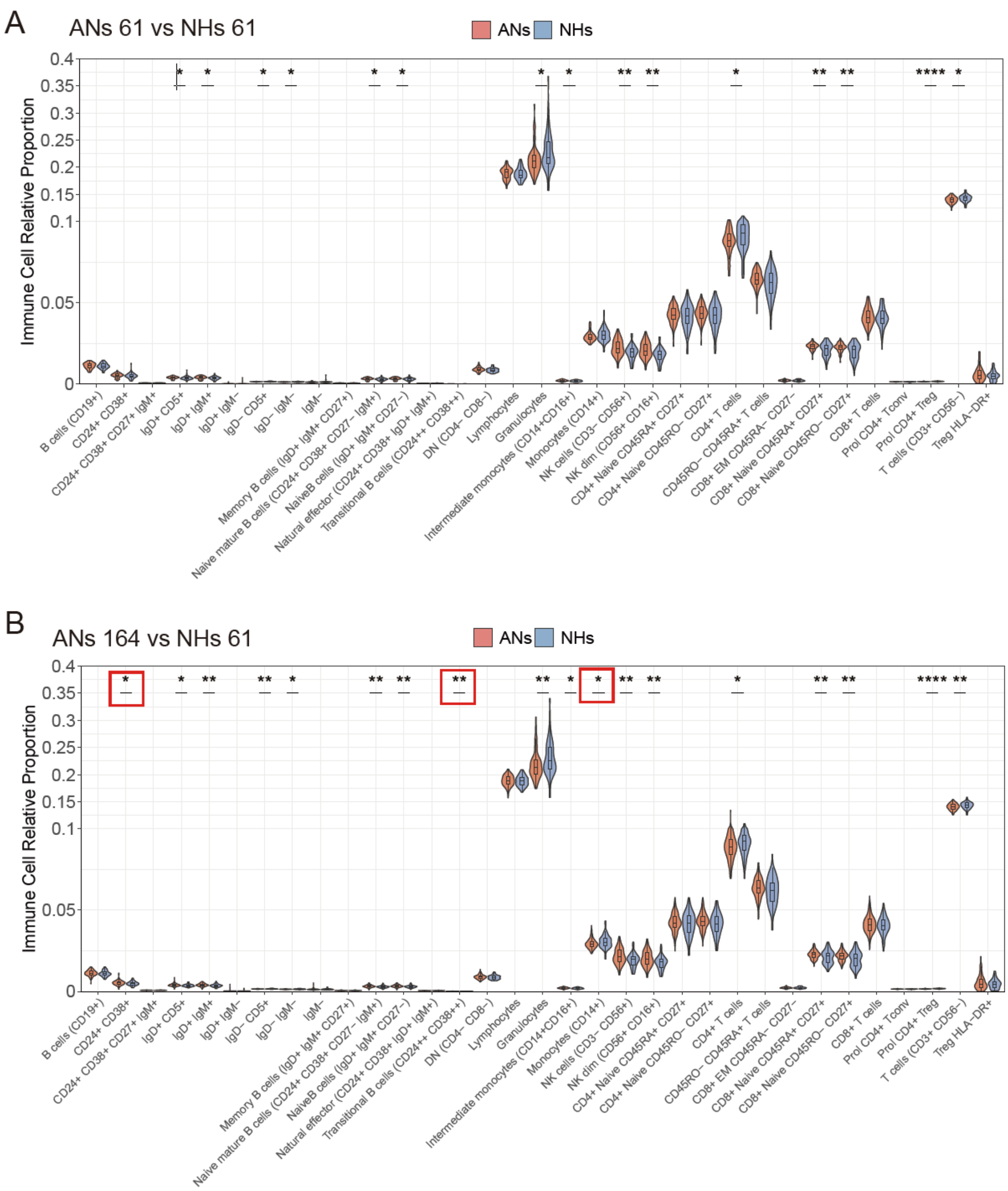

Figure 3 (A) Boxplot of immune cell proportion between 61 ANs and 61 NHs. (B) Boxplot of immune cell proportion between 164 ANs and 61 NHs.

We further validated these findings with CIBERSORTx, which confirmed significant differences in naive B cells, CD4 memory resting T cells, resting NK cells, and neutrophils—results concordant with those from Decon2 (Fig4).

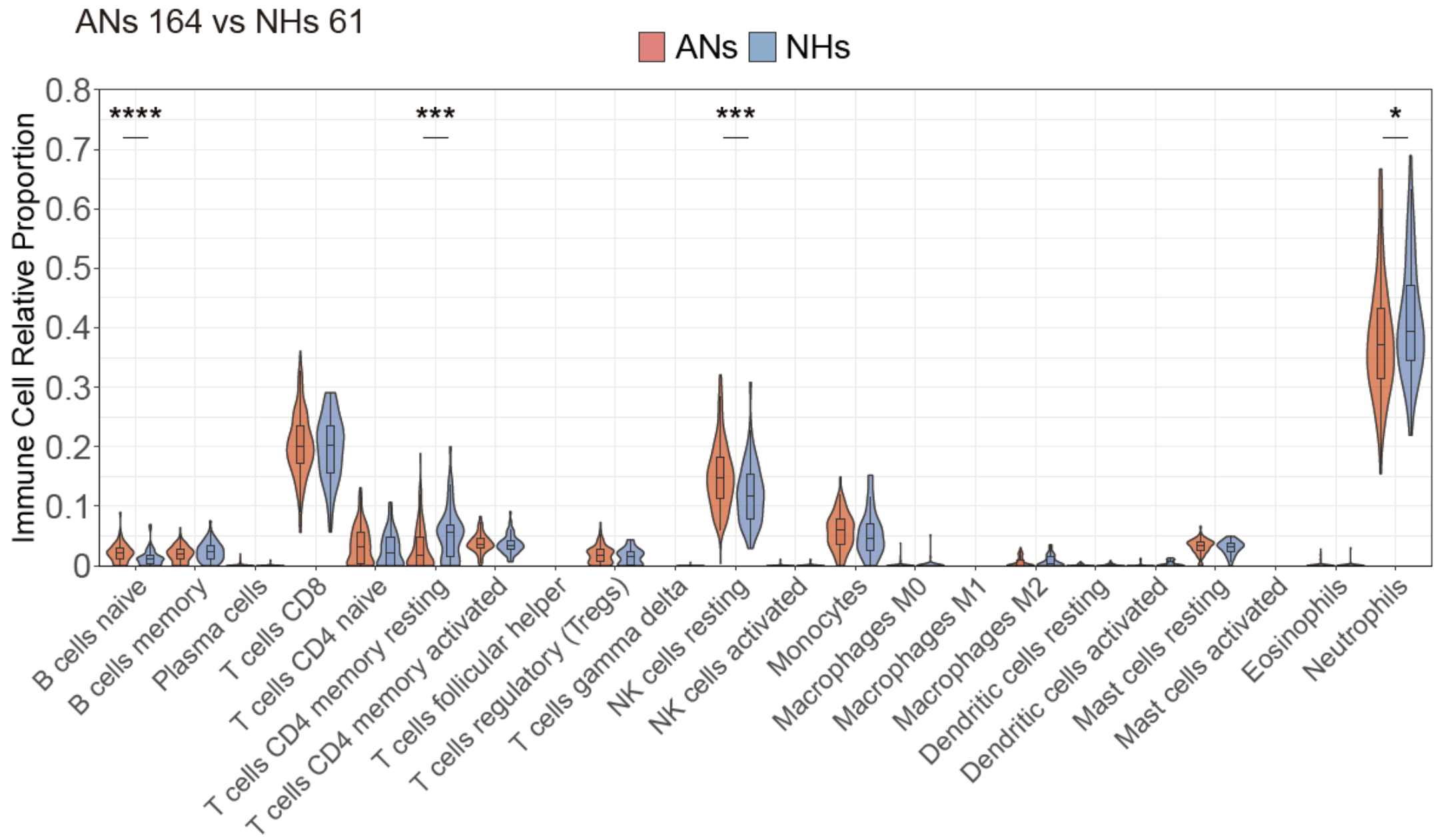

Figure 4. Boxplot of immune cell proportion between 164 ANs and 61 NHs using CIBERSORTx.

We computed the ieQTLs using the full sample set, so the results should not be influenced by the Tibetan(NHs) sample size.

2. Statistical Methodology and Reproducibility

Reviewer point #2:

eQTL Analysis: The manuscript mentions using QTLtools with permutations but lacks details on how covariates (age, sex, PCs, PEER factors) were selected and their impact on eQTL detection. The rationale for choosing FDR < 0.05 for cis-eQTLs and P < 1e-6 for trans-eQTLs should be justified.

Author response #2:

We sincerely appreciate the reviewer's insightful comments regarding our eQTL analysis approach. We have added a detailed description of covariate selection and threshold determination for the eQTL analysis in the Methods section (Line 456-464).

Regarding thresholds, cis-eQTLs were controlled at FDR < 0.05 using the Benjamini–Hochberg procedure, consistent with the GTEx v8 pipeline[2]. For trans-eQTLs, we adopted a nominal cutoff of $P < 1\times10^{-6}$ as recommended by the eQTLGen Consortium[3], balancing false discovery control with sensitivity to moderate-effect signals that may be relevant to high-altitude adaptation. We acknowledge that this threshold is more permissive than FDR correction and therefore interpret trans-eQTL findings with caution. Specifically, we included age and sex as

biological covariates, the top five genotype principal components (PCs) to adjust for population stratification, and five PEER factors to mitigate batch effects and hidden technical variation. This strategy follows widely used practices in GTEx and other large-scale eQTL studies.

Modified Line 456-464： Cis-eQTL were defined as regulatory loci within a 1-megabase (Mb) interval upstream and downstream of the gene start site regardless strand and were filtered using the Benjamini-Hochberg FDR < 0.05 according to GTEx v8 cis-QTL mapping pipeline. Trans-eQTL, defined as regulatory loci either on different chromosomes or more than 5 Mb away from the gene start site on the same chromosome, were selected with a P-value $< 1 \times 10^{-6}$ according to eQTLGen Consortium.We included age and sex as covariates to account for their effects on gene expression, the top 5 genotyping principal components (PCs) to control for population stratification, and 5 probabilistic estimation of expression residuals (PEERs) to mitigate the impact of batch effects and other unknown technical variables.

Ref:

2. Consortium, G. The GTEx Consortium atlas of genetic regulatory effects across human tissues. Science 369, 1318-1330 (2020).

3. Võsa, U. et al. Large-scale cis-and trans-eQTL analyses identify thousands of genetic loci and polygenic scores that regulate blood gene expression. Nature genetics 53, 1300-1310 (2021).

Reviewer point #3:

Causal Inference: The criteria for colocalization (PP4 > 0.7) and SMR (P < 0.05, HEIDI P > 0.05) are standard, but the manuscript does not address potential pleiotropy or LD confounding, especially for trans-eQTLs. For example, how were LD-pruned SNPs selected to avoid spurious associations?

Author response #3:

We sincerely appreciate the reviewer's insightful comments regarding potential pleiotropy and LD confounding in our causal inference analyses. First, We acknowledge a limitation of our study: while HEIDI test excludes major pleiotropic effects, subtle shared pathways may remain. Second, in order to avoid LD confounding of trans-eQTLs, we calculate the LD between 181 candidate causal trans-eQTLs. Among the 16,290 SNP pairs analyzed for LD, 16,122 pairs exhibited an R^2 value below 0.2, accounting for 98.97% of the total, which demonstrates minimal confounding by LD in our current results (Fig 5).

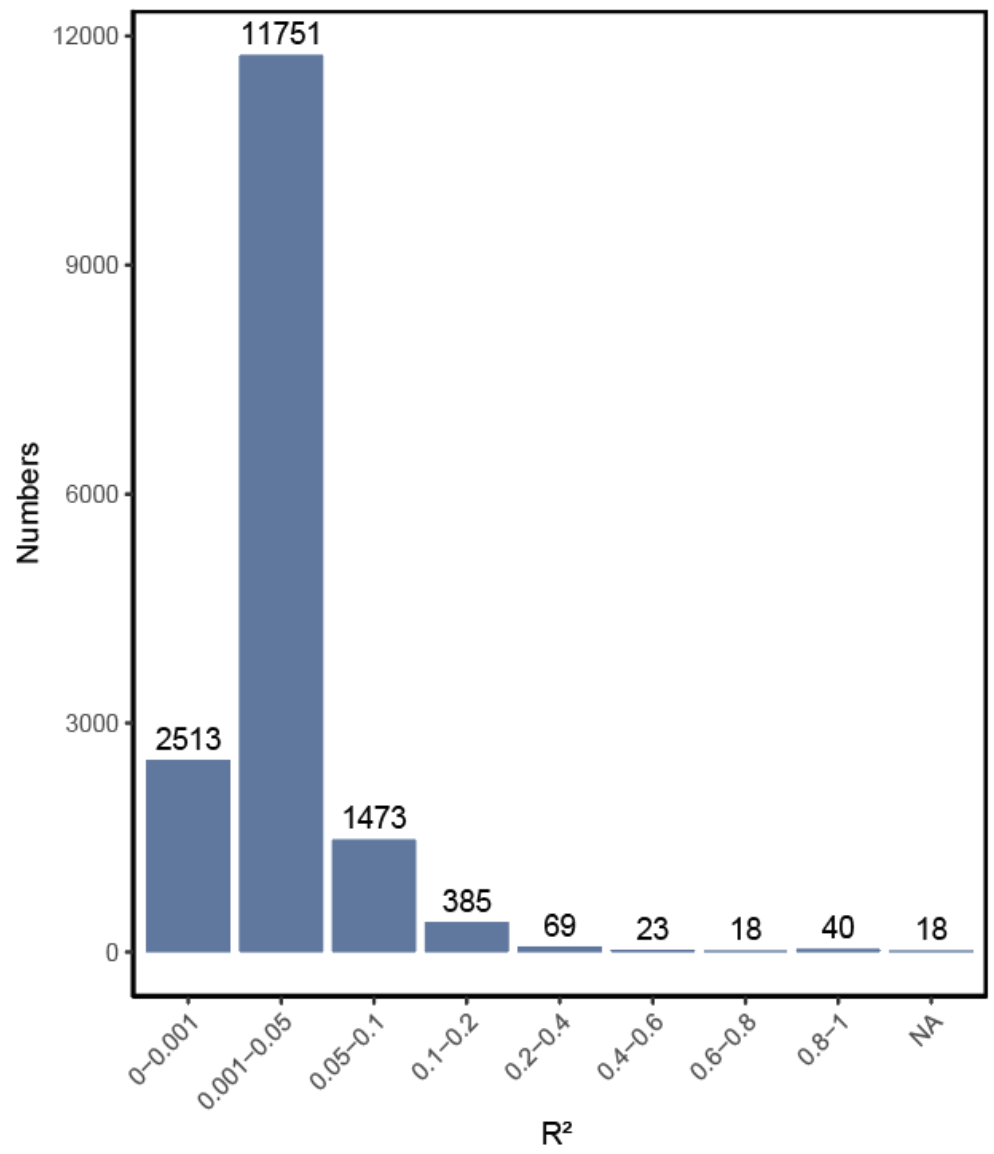

Figure 5. Bar plot showing the distribution of trans-eQTL SNP pairs across LD (R^2) bins.

Reviewer point #4:

Cell Type Deconvolution: The method for estimating immune cell proportions (Decon-cell) and identifying ieQTLs (Decon-QTL) requires more detail, including validation of cell type estimates against flow cytometry data (if available) and assessment of deconvolution accuracy.

Author response #4:

We sincerely appreciate the reviewer's insightful comments regarding our immune cell deconvolution approach. Because the blood samples have already been consumed, further validation by flow cytometry is no longer feasible. So we have implemented multiple validation strategies to ensure the robustness of our cell type estimates and ieQTL identification. We validated our findings using two independent cell type deconvolution approaches—CIBERSORTx and EPIC. CIBERSORTx revealed significant differences in naive B cells, CD4 memory resting T cells, resting NK cells, and neutrophils, whereas EPIC identified significant differences in $CD4^+$ T cells, monocytes, and neutrophils. Both sets of results are consistent with the cell type deconvolution outcomes obtained from Decon2. (Fig 6A, B).

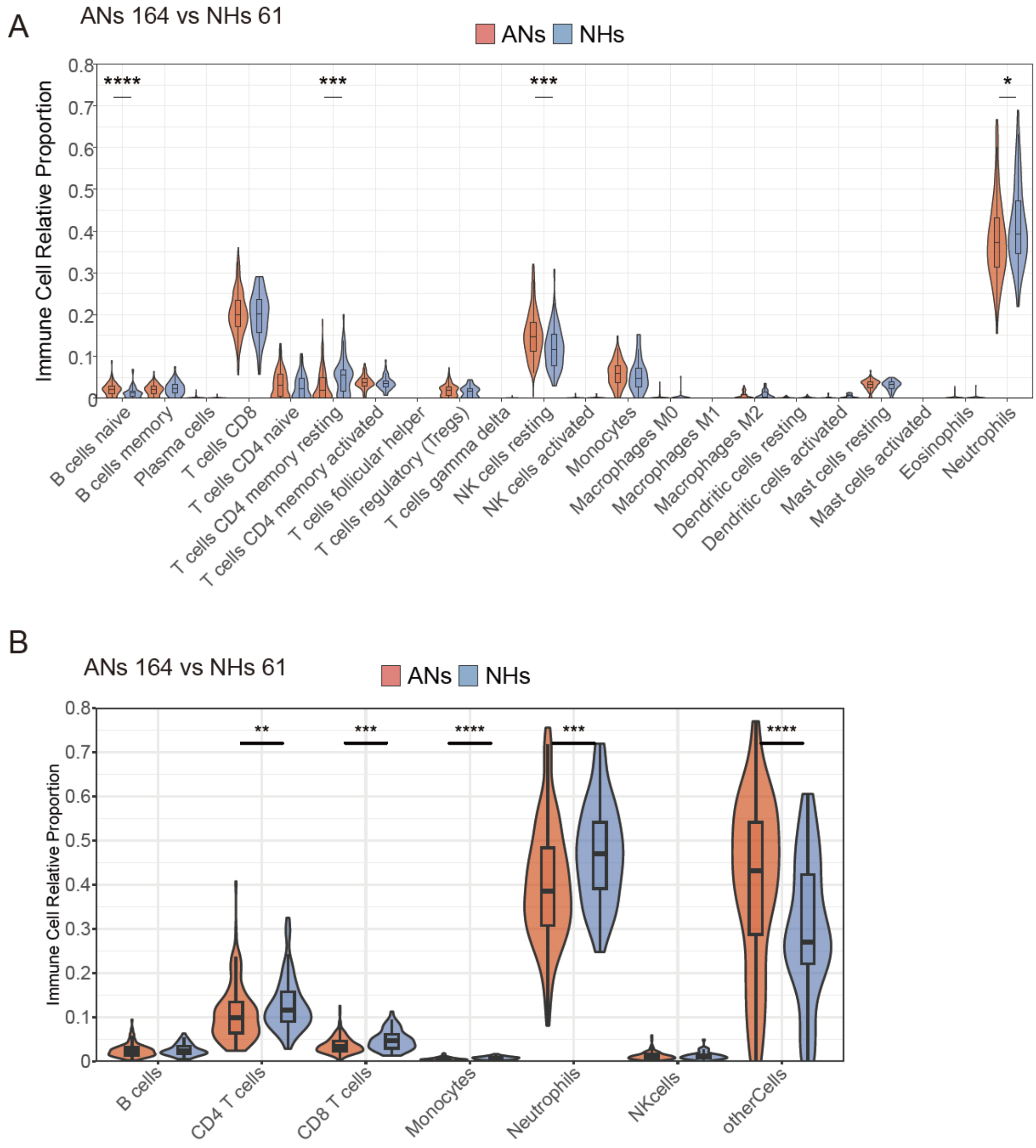

Figure 6 (A) Boxplot of immune cell proportion between 164 ANs and 61 NHs using CIBERSORTx. (B) Boxplot of immune cell proportion between 164 ANs and 61 NHs using EPIC.

We have also expanded the Methods section to include full details of the ieQTL identification pipeline (Line 467-473).

Modified Line 467-473: First, Decon-cell predicted proportions of 34 immune cell subpopulations from bulk RNA-seq data using elastic net regression (α=0.5, λ selected via 10-fold cross-validation), trained on FACS-quantified cell counts from the 500FG cohort. Next, Decon-eQTL integrated whole blood expression profiles, genotypes, and predicted cell proportions through a multivariate linear model that simultaneously tests genotype-by-cell-type interaction effects for six major immune populations (granulocytes, CD14+ monocytes,

CD4+/CD8+ T cells, B cells, NK cells).

Reviewer point #5:

The authors may like to expand the Methods section to include detailed parameter choices, covariate selection rationale, and validation of statistical approaches. Provide supplementary tables with full eQTL and causal inference results (e.g., all cis-eQTLs with FDR, colocalization PP values for top hits).

Author response #5:

Thanks for your valuable advice, we have expanded the Methods section to include more details of eQTL analysis (Line 456-473). We have uploaded full eQTL and causal inference results on Gitee (https://gitee.com/jinlongshi/eQTL_han_tibetan.git).

3. Functional Validation and Mechanistic Depth

Reviewer point #6:

While EXOC8 is validated, the manuscript identifies 1,254 causal genes, but only one is experimentally tested. This limits the generalizability of functional conclusions. The mechanism linking EXOC8 to erythroid differentiation is not explored (e.g., downstream pathways, interaction with known HAA genes like EPAS1/EGLN1).

The authors may like to either expand functional validation to 1-2 additional key genes (e.g., LILRB2, TPCN2) or provide in silico functional evidence (e.g., ChIP-seq binding of HIF-1α at EXOC8 locus, protein-protein interactions with erythroid differentiation factors) to strengthen mechanistic links.

Author response #6:

We sincerely appreciate the reviewer's valuable suggestions regarding the functional validation and mechanistic exploration of our candidate causal genes. We did more analysis to validate the EXOC8 functions in silico. We searched the GEO database for HIF1A and HIF2A ChIP-seq datasets (GSE106305, GSE200205, and GSE292665) and found no evidence of HIF1A or HIF2A binding within the EXOC8 locus. We then constructed a PPI network linking EXOC8 with HIF-pathway and erythropoiesis-related genes and identified its core subnetwork (Fig 7).

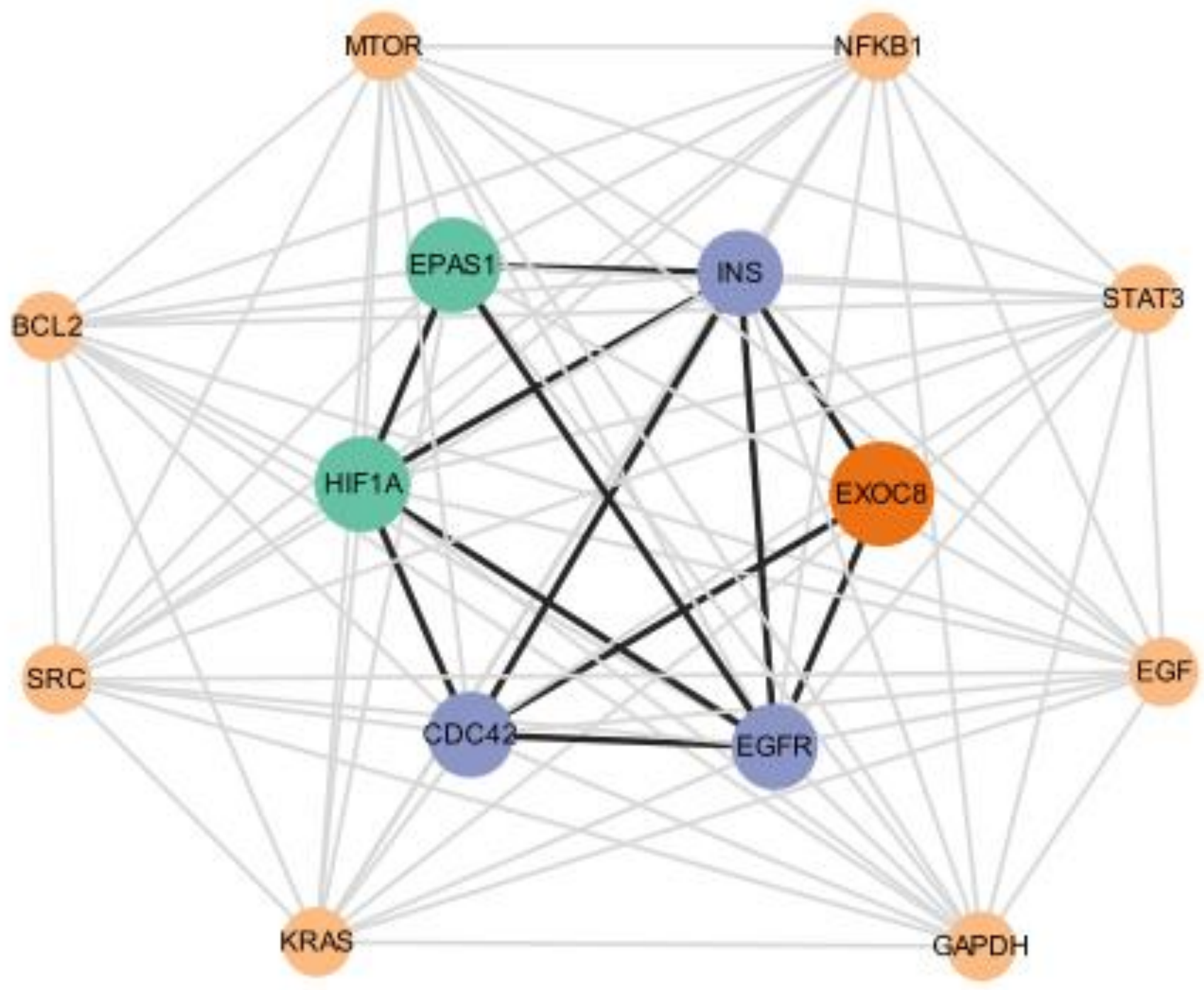

Figure 7 PPI network of EXOC8

Our analytical results reveal that EXOC8 engages in protein-protein interactions (PPIs) with key signaling molecules, including CDC42, INS, and EGFR. Notably, these three genes have been previously established as functional interactors of both HIF1A and EPAS1—core transcription factors mediating hypoxic responses. Furthermore, CDC42 and INS are known to positively regulate erythropoiesis[4,5], suggesting a plausible mechanistic pathway through which EXOC8 may influence erythropoiesis, potentially via modulation of CDC42 or INS activity. We have added these findings to the manuscript at Lines 253–257 and in Supplementary Figure S9C.

Ref:

3. Jin, J.C. et al. ROBO1 deficiency impairs HSPC homeostasis and erythropoiesis via CDC42 and predicts poor prognosis in MDS. Sci Adv 9, eadi7375 (2023).

4. Ratajczak, J. et al. The role of insulin (INS) and insulin-like growth factor-I (IGF-I) in regulating human erythropoiesis. Studies in vitro under serum-free conditions--comparison to other cytokines and growth factors. Leukemia 12, 371-81 (1998).

4. Interpretation of "Causal" Signals

Reviewer point #7:

Trans-eQTL Hotspots: The manuscript highlights trans-eQTLs as major contributors to HAA

heritability but does not address why trans-eQTLs (typically more variable across populations) show stronger enrichment than cis-eQTLs. Are trans-eQTLs more likely to be population-specific due to regulatory divergence?

Author response #7:

We sincerely appreciate the reviewer's insightful question regarding the population-specific patterns of trans-eQTLs in high-altitude adaptation (HAA). Compared with cis-eQTLs, trans-eQTL loci don't exhibit enrichment within regulatory regions (Fig 2G from manuscript); however, our analysis of Fst distribution patterns revealed that trans-eQTLs exhibit substantially greater population-specific than cis-eQTLs. Specifically, the number of trans-eQTLs with Fst > 0.1 was 14.6-fold higher than comparable cis-eQTLs (Fig 8).

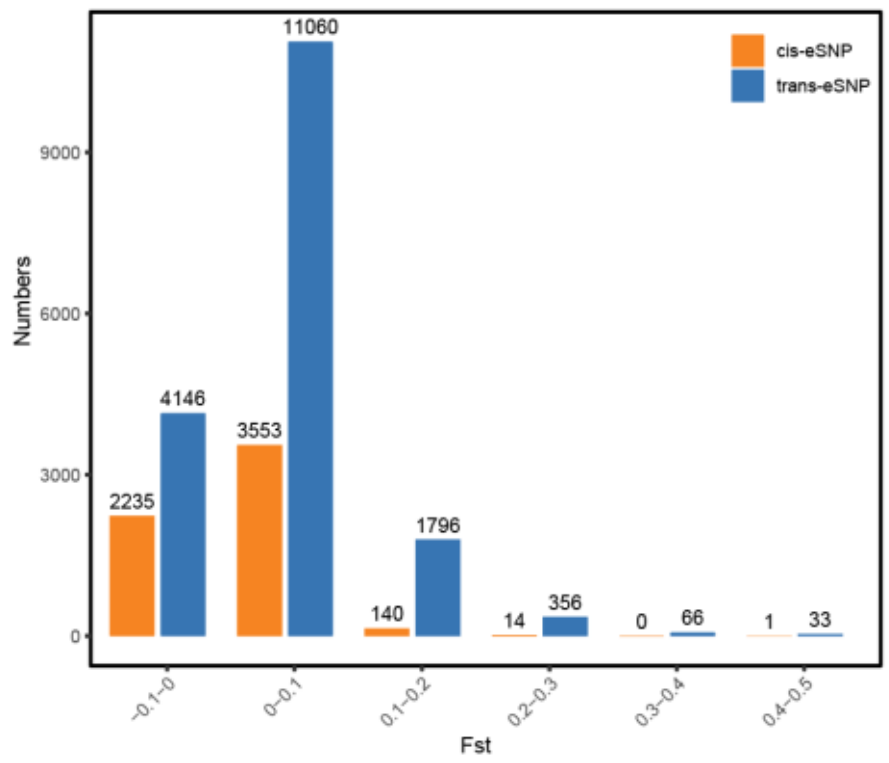

Figure 8. Bar plot showing the distribution of cis-eQTL and trans-eQTL number across Fst bins.

Reviewer point #8:

Overlap with Known HAA Genes: Classic HAA genes (EPAS1, EGLN1) are linked to trans-eQTLs regulating CPSF6, PURB, etc., but the biological relevance of these target genes to hypoxia adaptation (e.g., CPSF6 in mRNA polyadenylation under hypoxia) is underdeveloped. The authors may like to expand Discussion to interpret trans-eQTL enrichment in the context of HAA evolution (e.g., trans-regulatory networks as adaptive "modulators" of hypoxia responses). Link target genes of EPAS1/EGLN1 trans-eQTLs to known hypoxia pathways (e.g., HIF signaling, erythropoiesis).

Author response #8:

We sincerely appreciate the reviewer's insightful suggestions regarding the biological interpretation of trans-eQTL target genes in high-altitude adaptation (HAA). We have added discussion of the relationships between EPAS1/EGLN1 trans-eQTL target genes and known pathways involved in high-altitude adaptation (Line 347-350).

Added Line 347-350: PURB, identified as a trans-regulatory target gene of both EPAS1 and EGLN1, plays a pivotal role in hematopoietic differentiation and may significantly influence

erythropoiesis. Meanwhile, CPSF6—a distally regulated target of EPAS1—not only modulates angiogenesis but also serves essential functions in the temperature compensation mechanism of the circadian clock.

5. Clarity of Results and Figures

Reviewer point #9:

Figure 2H: The heritability enrichment plot is difficult to interpret. Labels for traits (e.g., "ALP") are unclear, and the rationale for selecting these traits (vs. key HAA traits like HGB, RBC) should be explained.

Author response #9:

Thanks for your valuable suggestions. We have added the full names of the hematological-phenotype abbreviations in the legend of Figure 2H and explained, in the Results section, the rationale for selecting these traits (Line 120-121).

Modified legend of Figure 2H: LD score regression partitioned heritability enrichments for cis-eQTL and trans-eQTL annotations across multiple HAA GWAS. Enrichments are colored by -log10(P) and error bars represent standard error (SE). UA, urine acid; RBC, Red blood Cell; PLT, Platelet; MPV, Mean Platelet Volume; MCV, Mean Corpuscular Volume; MCH, Mean Corpuscular Hemoglobin; MCHC, Mean Corpuscular Hemoglobin Concentration; Lym, Lymphocyte; HT, Height; HGB: Hemoglobin; HCT: Hematocrit; HBA1C, glycated hemoglobin; Eos, Eosinophil; CREA, creatinine; BUN, blood urea nitrogen; BMI, body mass index; Bas: Basophil; ALT: Alanine aminotransferase; ALP: alkaline phosphatase.

Modified Line 120-121: Here, we explored the specific patterns of genetic variants influencing HAA phenotypes which were different between Tibetan(NHs) and Han(ANs) populations.

Reviewer point #10:

Figure 7: The proposed mechanism model combines four modules but lacks direct links to genetic variants (e.g., which SNPs regulate genes in each module). Adding examples of causal SNPs/gene pairs (e.g., rs383925-LILRB2 in inflammation) would strengthen the model. The authors may like to revise figures to improve readability (e.g., clearer trait labels, color-coding for modules) and add insets explaining key interactions. Supplement with a table mapping top causal SNPs to their respective modules and traits.

Author response #10:

Thanks for your valuable advice. We have now annotated the causal SNPs in Figure 7 and provided the corresponding supplementary tables (S17).

Modified Figure 7:

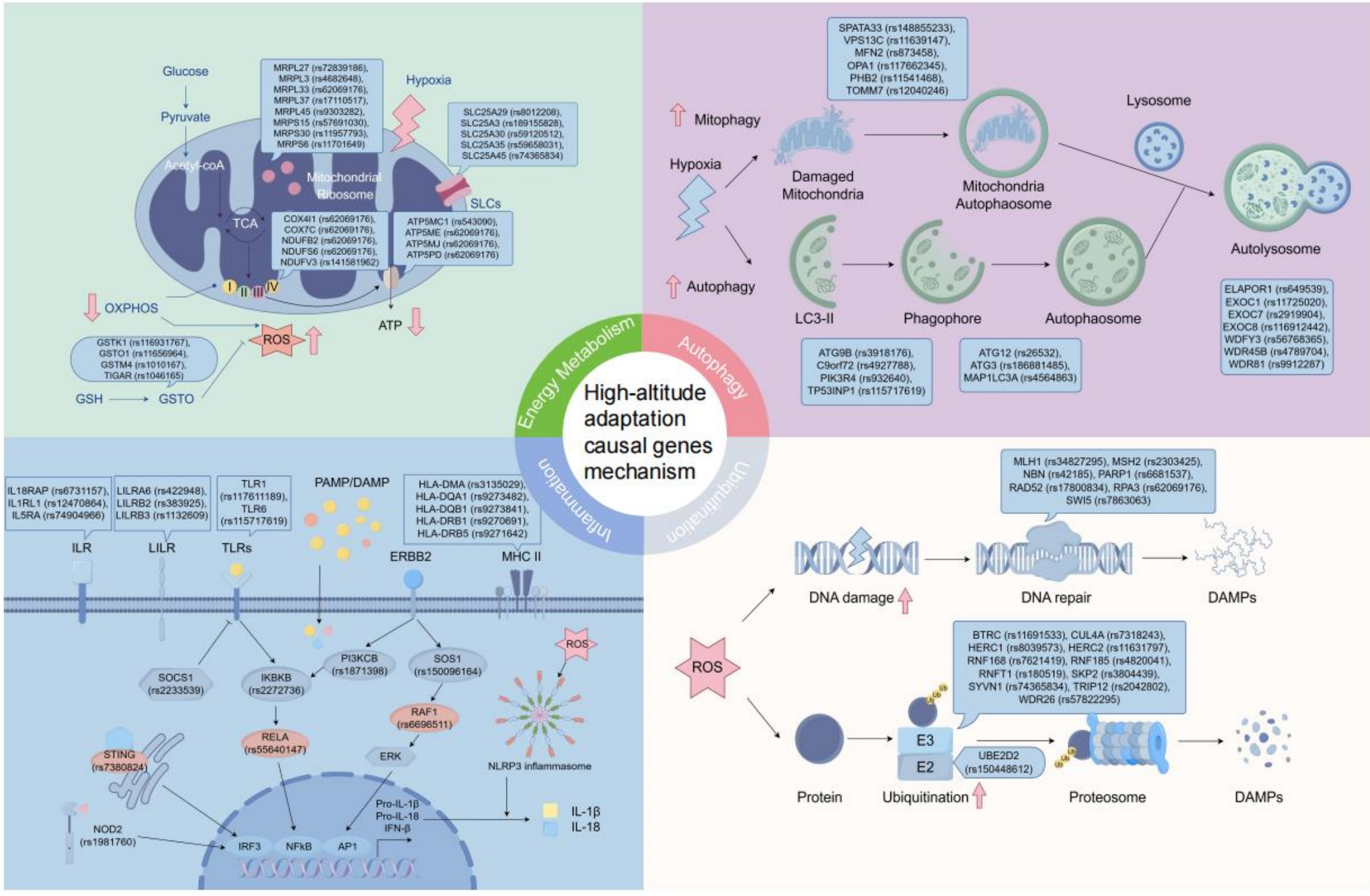

Minor Issues

Reviewer point #11:

Differential Expression Analysis: The manuscript mentions 27 hematological traits differing between Han and Tibetan but does not link these to differential gene expression (e.g., are genes in the energy metabolism module upregulated in Tibetans?).

Author response #11:

We sincerely appreciate the reviewer's insightful suggestion to better integrate our hematological trait findings with gene expression patterns. We have annotated the differential expression status of relevant genes in the multi-phenotype causal gene supplementary table to establish the relationship between differentially expressed genes and differential phenotypes (Supplementary table S8, 9, 10, 13). Furthermore, we analyzed the relationship between differential expression and causal gene modules, and calculated the correlation between core differentially expressed genes and phenotypes (Fig 9A, B). Most causal genes in the energy-metabolism module (46 of 76) were up-regulated in NHs (Supplementary table S17).

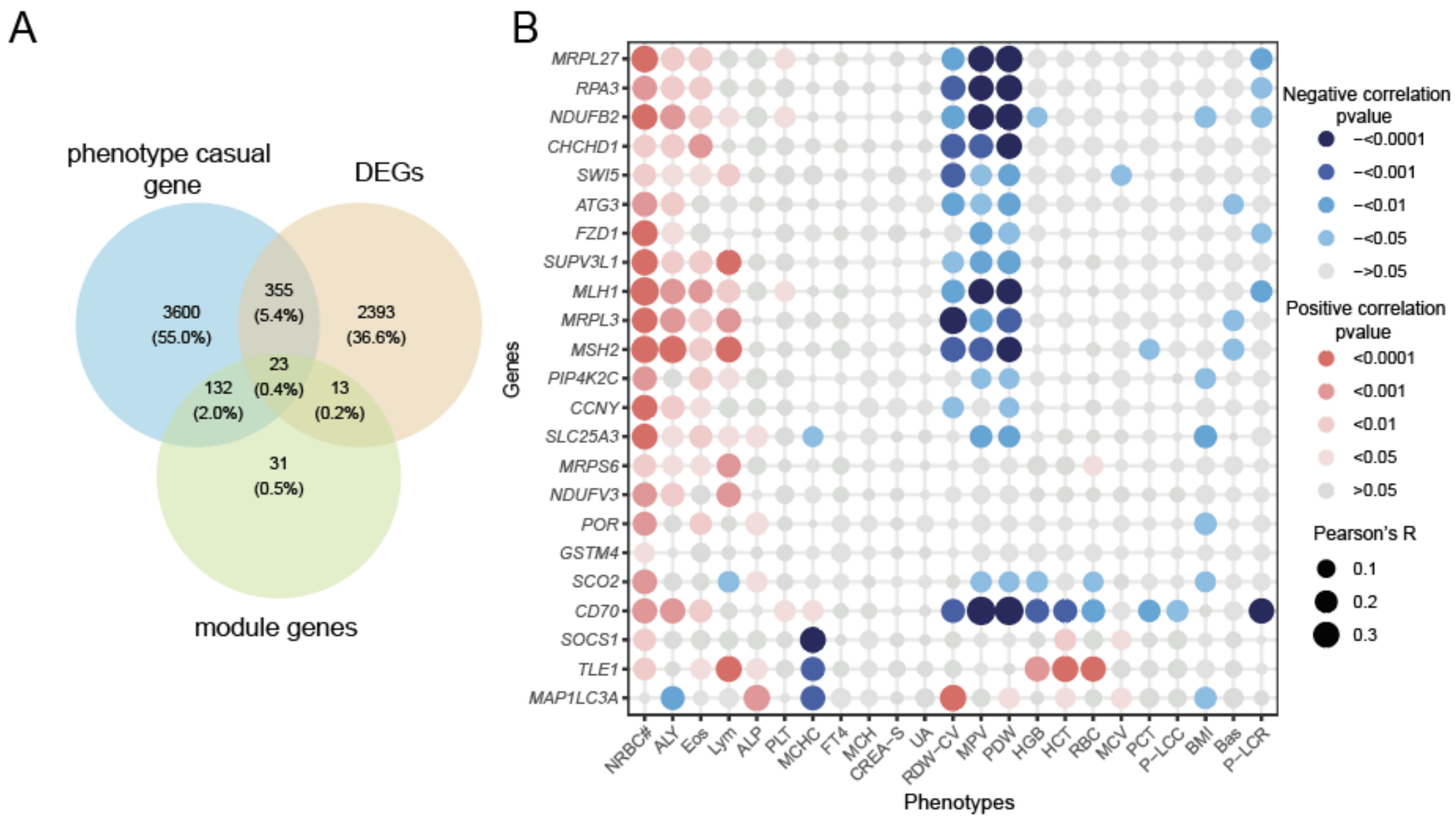

Figure 9 (A) Venn diagram of module genes, phenotype causal genes and DEGs. (B) Correlation heatmap between DEGs and differential phenotypes.

Reviewer point #12:

Some key HAA studies (e.g., 2023 Zheng et al. on Tibetan genome sequencing) are cited but not integrated into discussions of AGV overlap. The authors may like to add a supplementary figure correlating differential traits with module gene expression. Update discussions to contextualize findings with recent HAA literature.

Author response #12:

We sincerely appreciate the reviewer's constructive suggestions regarding the integration of recent high-altitude adaptation (HAA) studies and enhanced data visualization. In the Discussion, we have incorporated an in-depth review of the existing literature (Line 330-333); in addition, we have added a correlation heat map linking gene modules to phenotypes (Fig10). We have added these findings to the manuscript at Lines 397–399.

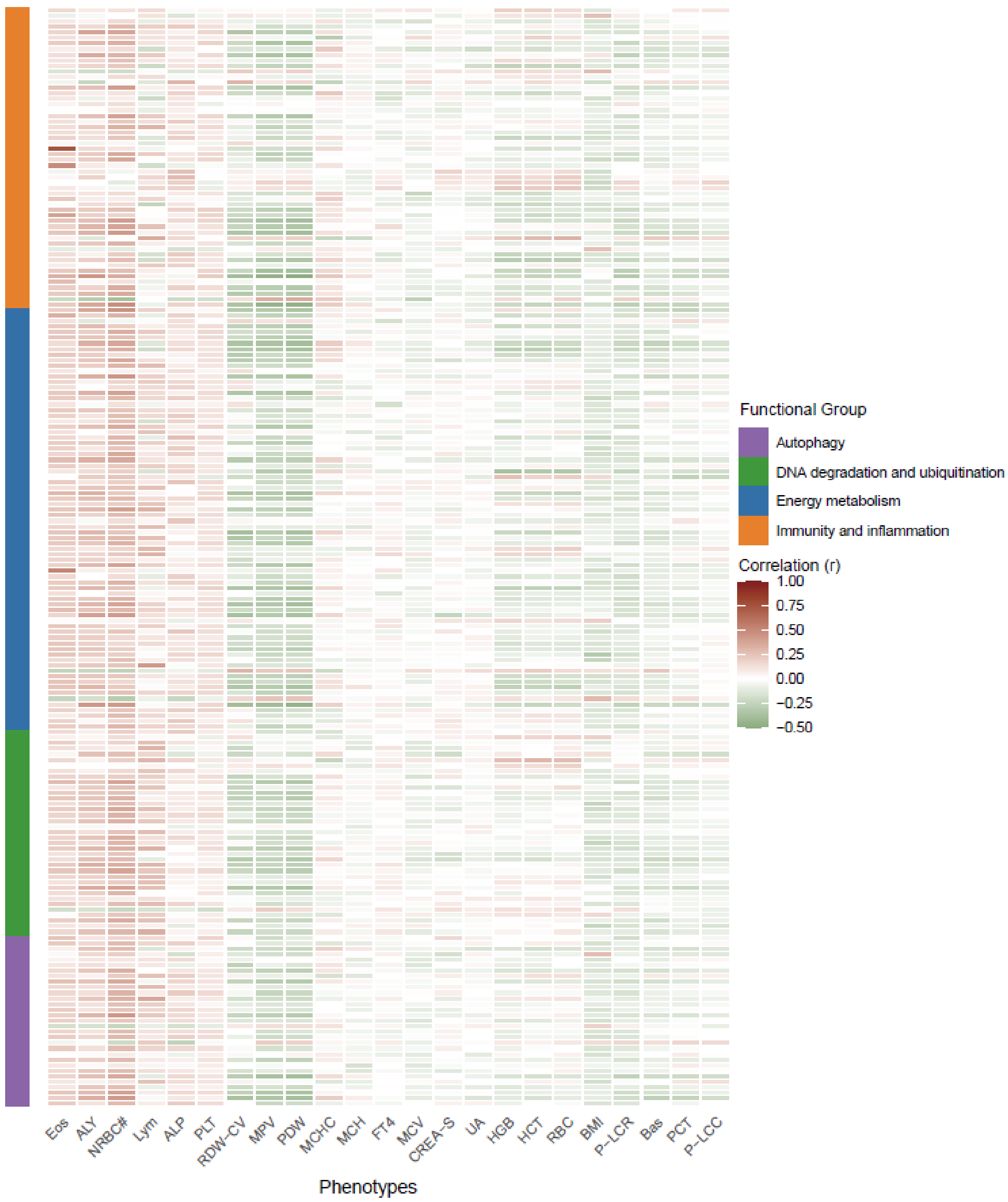

Figure 10 Correlation heatmap between module genes and differential phenotypes

Added Line 330-333: 24 AGVs were identified to be the same as reported candidate AGVs (Supplementary Table S15), such as rs2437150 (FST = 0.256) and rs1063321 (FST = 0.177) modulate the expression of SPRTN and HLA-DQB1, respectively were reported by Zheng et al. These replicated signals reinforce the reliability of our dataset and provide crucial insights into the genetic architecture underlying HAA.

Added Line 397-399: We further demonstrated that module genes are correlated with the differential phenotypes associated with high-altitude adaptation (Supplementary Fig S14, Table S18).

Reply to Reviewer #2

We are immensely grateful for the time and effort you have dedicated to reviewing our manuscript, as well as for the encouraging remarks on its strengths. We highly value your feedback and trust that our responses have satisfactorily resolved all the concerns you raised.

Notably, to better capture the characteristics of the study populations—Tibetans with long-term high-altitude adaptation and Han individuals with short-term high-altitude exposure—we have standardized the terminology throughout the manuscript, referring to them as *Native Highlanders (NHs)* and *Acclimatized Newcomers (ANs)*, respectively.

In the following responses, for ease of reading, we have used the notations *Tibetan (NHs)* and *Han (ANs)* consistently. Thank you.

Reviewer point #1:

The description of the discovery and replication datasets used for eQTL consistency analysis is unclear. The authors have briefly described the datasets for eQTL consistency analysis in the Methods section, but the description is simple and with little details. The authors should briefly state the basic ideas of the dataset design in the corresponding Results section and describe the details clearly in the Methods section.

Author response #1:

Thanks for your valuable suggestions. We have added a detailed description of the discovery and replication datasets used for eQTL consistency analysis in the Methods section (Line 479-481).

Modified Line 478-480: We employed AC and π1 to assess the concordance of eQTL effects across different population datasets including Han(ANs)-Tibetan(NHs), Tibetan(NHs), Han(ANs), GTEx and eQTLGen. When performing consistency comparisons between datasets, we typically designate one dataset as the discovery set, while the remaining datasets serve as validation sets, based on which relevant consistency metrics are calculated.

Reviewer point #2:

The phenotype of 58 in Figure 1 is misleading as the corresponding numbers for Genome and Transcriptome is the numbers of subjects in the cohort but not the number of features.

Author response #2:

Thanks for your valuable advice. We have revised Figure 1 to ensure that the number of phenotypes shown is unambiguous.

Modified Figure 1:

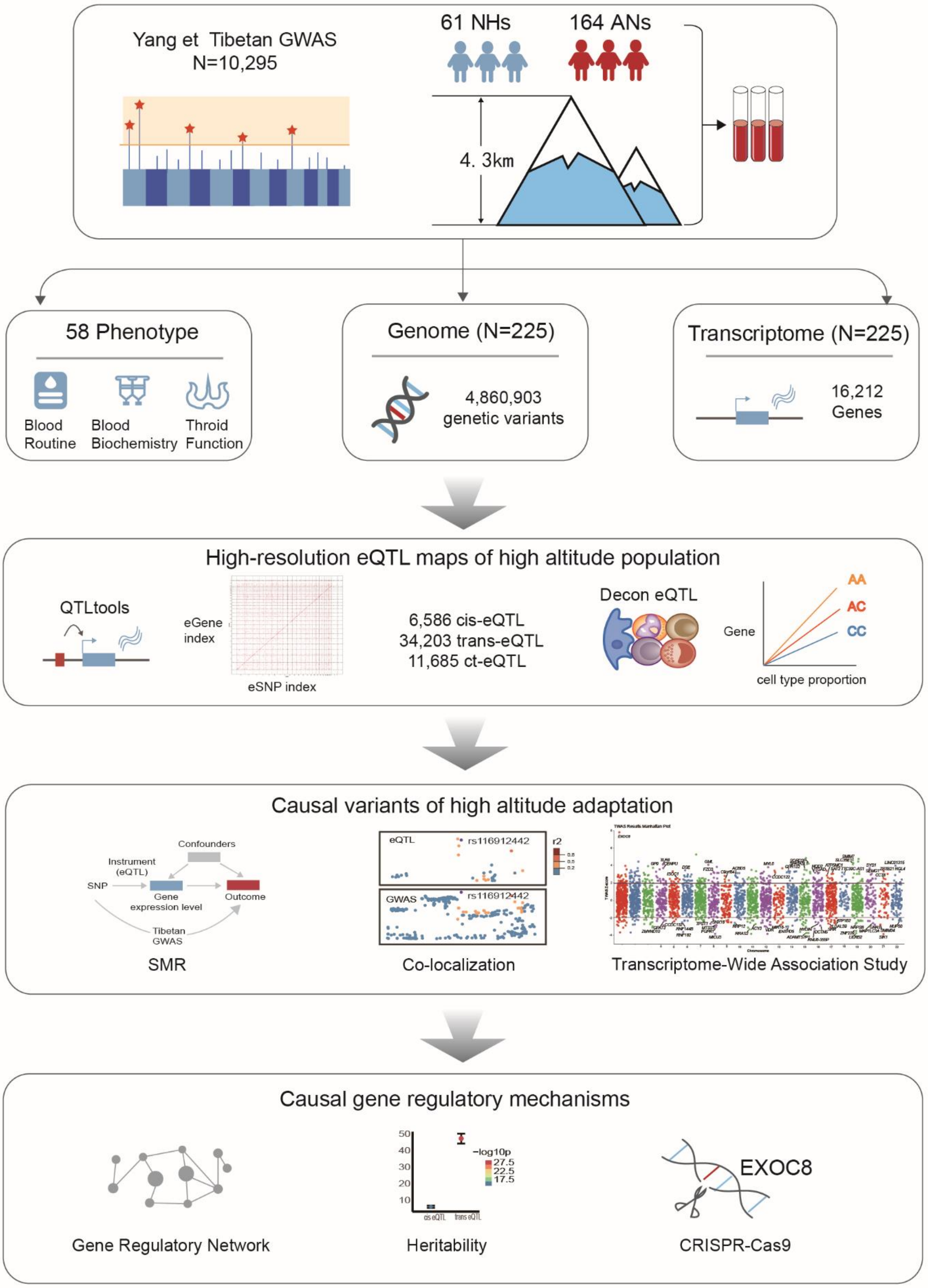

Reviewer point #3:

Full name of chromatin states should be stated in the legend of Figure 2G.

Author response #3:

Thanks for your valuable advice. We have added full name of chromatin states in the legend of Figure 2G.

Modified legend of Figure 2G: Enrichment of eQTLs in chromatin state. TssA (Active TSS),

TssAFlnk (Flanking active TSS), TxFlnk (Transcr. at gene 5' and 3'), Tx (Strong transcription), TxWk (Weak transcription), EhnG (Genic enhancers), Ehn (Enhancers), Znf/Rpts (ZNF genes + repeats), Het (Heterochromatin), TssBiv (Bivalent/poised Tss), BivFlnk (Flanking bivalent Tss/Enh), EnhBiv (Bivalent enhancer), ReprPC (Repressed Polycomb), ReprPCWk (Weak repressed Polycomb), Quies (Quiescent/low).

Reviewer point #4:

Typos: in Figure 2D, a space is missing between from and TSS; in Figure 2E, "od" should be "of".

Author response #4:

Thanks for your valuable advice. We have revised the typos in Figure 2D and Figure 2E.

Modified Figure 2D and 2E:

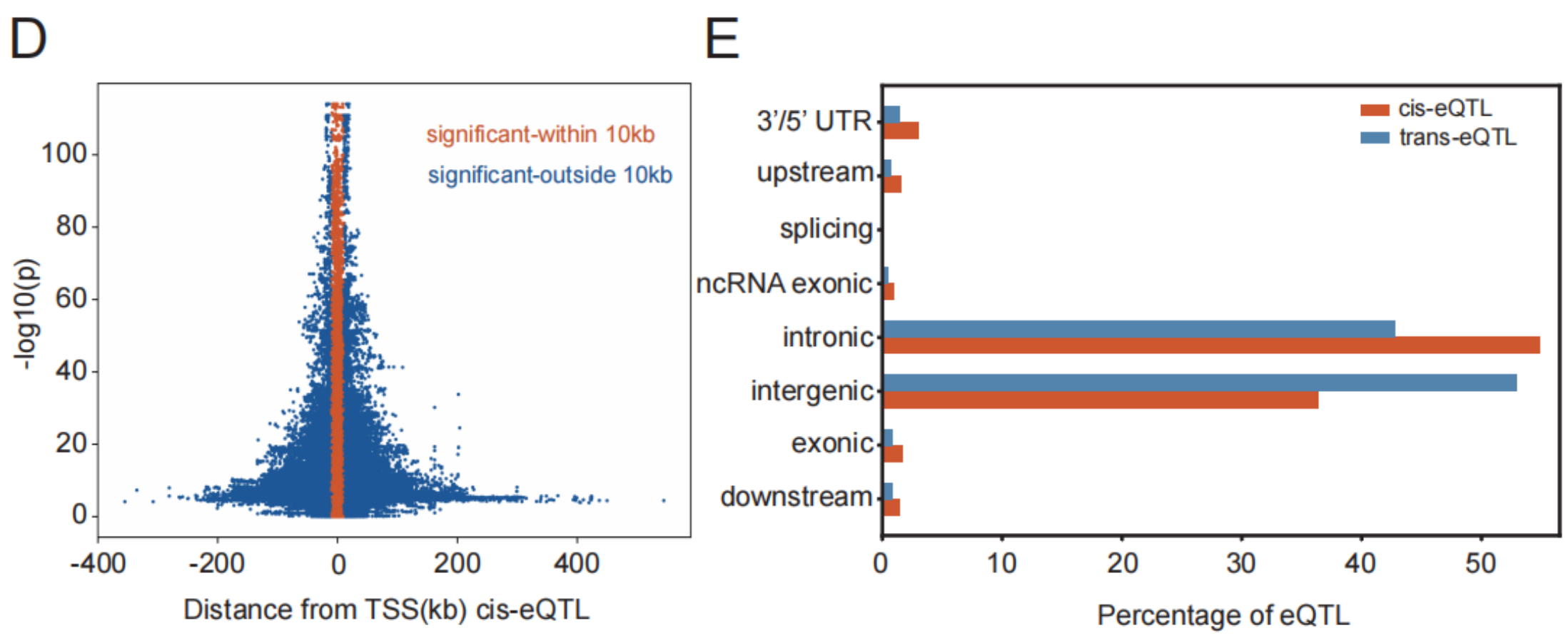

Reviewer point #5:

All the proteins should be in standard form not italic form. Line 206: "Under hypoxic stress, Notch1, in partnership with HIF-1α, orchestrates the regulation of ...". The Notch1 should be NOTCH1 if the protein of this gene is indicated.

Author response #5:

Thank you for pointing out the formatting errors in the protein spellings, we have made the corresponding corrections.

Modified Line 209: Under hypoxic stress, Notch1, in partnership with HIF-1α, orchestrates the regulation of hypoxia-responsive genes.

Reviewer point #6:

Line 246: A space is missing in "TLR6and DSE".

Author response #6:

Thank you for pointing out the formatting errors, we have added the space in Line 249.

Modified Line 249: Among these, five novel HAA-related genes were discovered: EXOC8, CREB5, MYL6, TLR6 and DSE.

Reply to Reviewer #3

We sincerely thank you for the insightful comments and constructive suggestions, which have helped us to significantly improve the clarity, accuracy, and overall quality of our manuscript. We have carefully addressed every point raised and have revised the manuscript accordingly. Below, we provide a detailed response to each comment.

To better capture the characteristics of the study populations—Tibetans with long-term high-altitude adaptation and Han individuals with short-term high-altitude exposure—we have standardized the terminology throughout the manuscript, referring to them as *Native Highlanders (NHs)* and *Acclimatized Newcomers (ANs)*, respectively.

In the following responses, for ease of reading, we have used the notations *Tibetan (NHs)* and *Han (ANs)* consistently. Thank you.

Reviewer point #1:

Line 27: The authors claim to have "identified several novel causal genes". Only causal gene with strong statistical or experimental evidence is EXOC8.

Author response #1:

We sincerely appreciate the reviewer's careful evaluation of our causal gene claims. We have revised the overstatement in the abstract.

Modified Line 28: identified several novel candidate causal genes, including *EXOC8*.

Reviewer point #2:

Line 31: They claim their study "reveals causal chain of variant-gene-phenotype for HAA". There is no direct evidence that the phenotype is the target of selection for HAA. It should be toned down as "reveals a causal chain of variant-gene-phenotype for an HAA-related trait."

Author response #2:

We have revised the statement in Line 31 according to your advice.

Modified Line 32: offers a comprehensive eQTL map and reveals causal chain of "variant-gene-phenotype" for an HAA-related trait.

Reviewer point #3:

Lines 73-78: Mann-Whitney test was used here to test if there exists a significant difference in the phenotypic distributions between Hans and Tibetans. First, it is not very clear what is being done here without digging into Table S2. Second, how is this analysis relevant for HAA? It does not necessarily tell that these trait differences are genetic, let alone driven by HAA.

Author response #3:

We sincerely appreciate the reviewer's insightful comments regarding our phenotypic comparison analysis between Han(ANs) and Tibetan(NHs) populations. We fully agree that simply identifying differences in phenotypic distributions does not directly prove that these traits are genetically driven or necessarily related to high-altitude adaptation (HAA). Below we provide a detailed explanation addressing this question.

The purpose of this analysis was to first establish which physiological traits show consistent and significant divergence between Han(ANs) and Tibetan(NHs) populations, since these traits have been repeatedly implicated as hallmarks of HAA in previous studies. We systematically compared 58 phenotypic traits using the Mann-Whitney U test and identified 28 significant differences ($P < 0.05$), including body mass index (BMI) and 27 hematological and biochemical traits such as hemoglobin (HGB), red blood cell count (RBC), hematocrit (HCT), eosinophils (Eos), creatinine (CREA), uric acid (UA), and free thyroxine (FT4). These traits overlap substantially with previously reported Tibetan(NHs)-specific physiological features associated with oxygen transport and metabolic adaptation at high altitude[6].

Importantly, our study does not stop at phenotypic description. In subsequent sections of the manuscript, we further investigated the genetic basis of these Tibetan(NHs)-specific phenotypes through genome-wide association and eQTL analyses. This integrative framework allowed us to move beyond descriptive comparisons and directly test whether the observed phenotypic divergences are underlain by genetic variation relevant to HAA.

Also, we have added a statement in lines 74–80 of the main text to clarify the purpose and methodology of this analysis.

Modified Line 74-80 : To systematically investigate the physiological adaptations in Tibetan(NHs) associated with chronic high-altitude exposure, we conducted a comprehensive comparative analysis of 58 phenotypic traits between Han(ANs) and Tibetan(NHs) populations using the Mann-Whitney U test. We we identified 28 distinct traits (P-value < 0.05) including body mass index (BMI), and 27 hematological traits such as hemoglobin (HGB), red blood cell count (RBC), hematocrit (HCT), eosinophils (Eos), creatinine (CREA), uric acid (UA), and free thyroxine (FT4), through routine blood tests, serum biochemistry, and thyroid function

assessments.

Ref:

5. He, Y. et al. Deep phenotyping of 11,880 highlanders reveals novel adaptive traits in native Tibetans. iScience 26, 107677 (2023).

Reviewer point #4:

Lines 78-86: Exactly how GSMR was used here? It is not very clear. How does this lead to the claim that these traits are "causally associated with Tibetan high-altitude adaptation"? There are no details in the Methods section about how they selected SNPs or by which criteria they chose outcome traits. More importantly, why is Tibetan (is this Yang-GWAS?) an exposure, and why are the physiological traits outcomes here? Shouldn't the test be the other way around? Isn't it more likely that larger effects for physiological traits cause stronger selection (i.e., greater allele frequency differences in Yang-GWAS)?

Author response #4:

We sincerely appreciate the reviewer's insightful questions regarding our GSMR analysis. We agree that the rational and methodological details in the initial submission were not sufficiently clear.

In fact, the GSMR analysis serves as further causal validation of Tibetan(NHs) high-altitude adaptive phenotypes. Since Tibetan(NHs) are a long-term high-altitude dwelling population, their physiological characteristics have been regarded to represent high-altitude adaptation[7]. Therefore, we hypothesized that Yang-GWAS reflects high-altitude adaptation, and subsequently sought to verify the causal relationship between high-altitude adaptation and related physiological phenotypes. The study by Jiang et al. employed the same analytical approach[8]. We actually performed bidirectional association analyses (Table 1), but given that the primary objective of this study is to explain how high-altitude adaptation leads to specific phenotypic outcomes, we treated it as the exposure and other physiological traits as outcomes in our presentation.

We have supplemented the methodological details regarding GSMR instrumental variable selection in the Methods section, including genome-wide significance thresholds, LD pruning, and the HEIDI-outlier filtering to reduce pleiotropic bias (Line 506).

Table 1. Causal relationships between HAA and physiological traits

Exposure	Outcome	beta	se	pvalue	nsnp	multi_snp_based_heidi_outlier
HAA	WT	-1.30664	0.255349	3.10E-07	9	0.307384
PLT	HAA	-0.008655	0.0019237	6.82E-06	257	0.253429

ALT	HAA	0.0193843	0.0049121	7.94E-05	34	0.105527
HAA	HGB	-1.17113	0.304476	1.20E-04	9	0.160872
HAA	HT	-0.797746	0.209228	1.37E-04	8	0.348417
HAA	HCT	-1.16894	0.308988	1.55E-04	9	0.171115
HAA	RBC	-1.14387	0.307612	2.00E-04	9	0.0394788
WC	HAA	0.0226869	0.0063881	3.83E-04	8	0.267634
HAA	PLT	-0.84154	0.308203	0.0063244	9	0.396875
HAA	HBA1C	-1.26586	0.471231	0.0072252	12	0.309384
CREA	HAA	0.0170964	0.0065949	0.0095319	66	0.363175
HAA	BMI	-0.770597	0.305329	0.0116084	9	0.302613
HAA	CREA	0.506167	0.209899	0.0158881	9	0.231123
UA	HAA	-0.003927	0.0016947	0.0204894	150	0.0441572
HAA	ALT	-0.717007	0.320846	0.0254344	9	0.415017
HAA	BUN	-0.742238	0.336718	0.027501	9	0.208552

Added Line 525-528: GSMR uses SNPs associated with the risk factor (those with $P < 5e\text{-}8$) as the instruments variables, and excludes variants in linkage disequilibrium (LD, $R^2 > 0.05$). It uses the HEIDI-outlier filtering approach to remove SNPs that have effects on both the exposure and the outcome because of pleiotropy.

Ref:

6. Gilbert-Kawai, E.T., Milledge, J.S., Grocott, M.P. & Martin, D.S. King of the mountains: Tibetan and Sherpa physiological adaptations for life at high altitude. Physiology 29, 388-402 (2014).

7. Jiang, Y. et al. Associations between high-altitude adaptation and risk of cardiovascular diseases: a bidirectional Mendelian randomization study. Mol Genet Genomics 298, 1007-1021 (2023).

Reviewer point #5:

Lines 120-124 (Figure 2H): Yang-GWAS is not exactly a GWAS here. What is the measured phenotype, and how is the heritability defined? It is a binary phenotype, but it is hard to see how there could be an underlying liability distribution for Tibetanness, like for hypertension. In addition, authors need to ensure that the population stratification is not driving the high enrichment. Note that eQTL (especially, trans-eQTL) can be inflated by stratification, and Yang-GWAS signals are also stronger in highly divergent SNPs between populations.

Author response #5:

We sincerely appreciate the reviewer's insightful questions regarding the interpretation of Yang-

GWAS signals and potential stratification effects. We agree that Yang-GWAS does not represent a conventional GWAS of a continuous or disease trait, but rather a binary classification ("Tibetan(NHs) VS Han(ANs)") that captures population divergence. In current research on high-altitude adaptation, howerver, this dataset is universally adopted as the definitive phenotypic representation of high-altitude adaptation, because long-term Tibetan residency at high altitude embodies the composite outcome of adaptive processes[8,9].

Since we consider Tibetan(NHs) high-altitude adaptation as an integrated phenotype, the population stratification signals inherently represent the biological signals of interest to our study. We intentionally preserved these signals to establish the association between eQTLs and high-altitude adaptation.

Ref:

7. Jiang, Y. et al. Associations between high-altitude adaptation and risk of cardiovascular diseases: a bidirectional Mendelian randomization study. Mol Genet Genomics 298, 1007-1021 (2023).

8. Cheng, F. et al. Distinct methylomic signatures of high-altitude acclimatization and adaptation in the Tibetan Plateau. Cell Discov 11, 45 (2025).

Reviewer point #6:

Line 158: What is the corresponding FDR? If the cutoff here ("P < 0.05") is the nominal P value, it would be too lenient, especially for trans-QTL.

Author response #6:

Thanks for your valuable advice. We agree with the reviewer that relying solely on a nominal $P < 0.05$ threshold can be overly permissive, especially for trans-QTLs. To address this, we have now provided the corresponding FDR values in Supplementary Table S7.

As noted, applying FDR < 0.05 retained only a very limited number of signals, which may underestimate the polygenic nature of trans-regulation. Therefore, in the main text we chose to report results at the nominal $P < 0.05$ threshold to maximize sensitivity and capture a broader set of candidate trans-QTLs.

Reviewer point #7:

Line 176: This is not "causal inference." Neither SMR, TWAS, nor Coloc has the specificity to establish a causality. It only tests for the shared causal effects between traits, which is far from causality.

Author response #7:

Thank you for this valuable suggestion. We fully agree with the reviewer that methods such as SMR, TWAS, and Coloc cannot establish strict causality, but rather test whether two traits share the same underlying genetic signal. We have revised Line 178 accordingly, replacing "causal inference" with "shared genetic architecture of cis-eQTLs and HAA traits."

We believe this revised terminology more accurately reflects the analytical framework: these approaches identify loci where genetic variants influence both gene expression and HAA-associated traits, thereby highlighting putative functional links without making strong causal claims. This clarification ensures that our interpretation is precise and avoids overstating the methodological specificity.

Modified Line 179: Shared genetic architecture of cis-eQTLs and HAA Traits

Reviewer point #8:

Lines 220-239: To suggest "trans-causal variants," the trans-eQTL signals should be fine-mapped down to a SNP level. Are these 181 trans-causal variants fine-mapped? Further, positive selection drives Fst up for linked SNPs in flanking regions by LD (genetic hitchhiking). This effect is more extensive if the adaptation is relatively recent. The authors presented no evidence here to support that the same causal variants drive both trans-eQTL and HAA.

Author response #8:

We sincerely appreciate the reviewer's insightful suggestions regarding trans-eQTL fine-mapping and selection signal interpretation.

We identified 181 trans-eQTL loci through SMR analysis, applying LD pruning to exclude variants in strong linkage disequilibrium. To substantiate the reliability of these loci, we conducted comprehensive functional genomic annotation covering chromatin states, and transcription factor motif binding (Supplementary Table S11).

Regarding signals of positive selection, we acknowledge the reviewer's point that elevated Fst values can also arise from linked hitchhiking effects, especially in cases of recent adaptation. These trans-eQTL loci indeed exhibit signatures of positive selection, as evidenced by elevated Fst (Fig 11A), yet they remain mutually independent with respect to linkage disequilibrium (Fig 11B). Supporting evidence that the same causal variants jointly drive both trans-eQTL associations and HAA is provided in Supplementary Table 10.

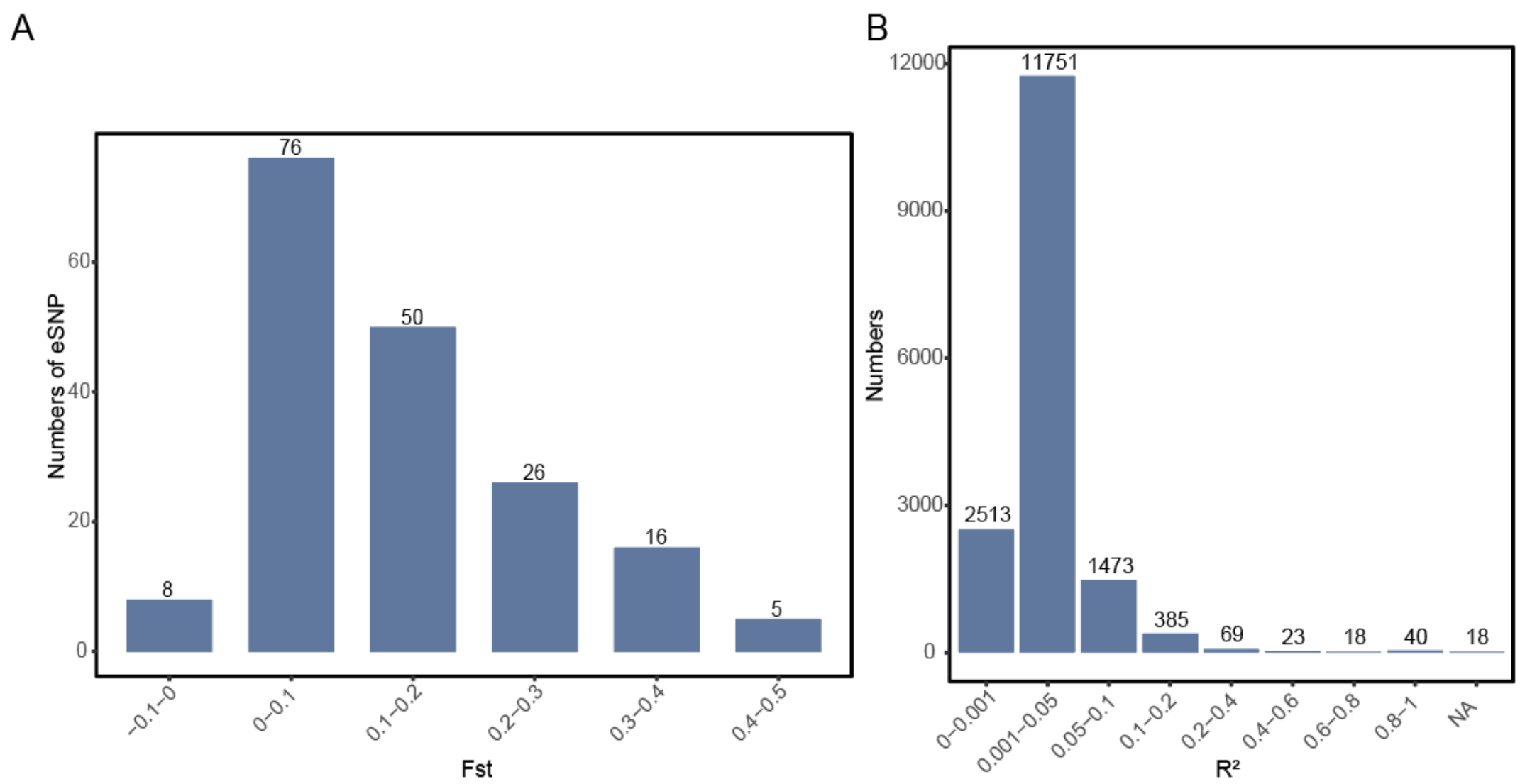

Figure 11 (A) Bar plot showing the distribution of trans-eQTL number across Fst bins. (B) Bar plot showing the distribution of trans-eQTL SNP pairs across LD (R^2) bins.

Reviewer point #9:

Lines 337-339 ("We identified 145 novel causal chains..."): This is an overstatement.

Author response #9:

We have revised the statement in Line 352 according to your advice.

Modified Line 351: we identified 145 candidate causal chains via cis-eQTL

Reviewer point #10:

Data availability: It appears that genome-wide summary statistics are not freely available without restriction for eQTL at https://ngdc.cncb.ac.cn.

Author response #10:

We sincerely appreciate the reviewer's attention to data accessibility. To ensure full transparency and facilitate research reproducibility, all eQTL statistics are deposited in a publicly accessible repository on Gitee (https://gitee.com/jinlongshi/eQTL_han_tibetan.git). GWAS summary are available on https://cnsgenomics.com/data/yang_et_al_2017_pnas.html.

Dear Dr. Ani Manichaikul,

We sincerely thank you and reviewers for your thorough evaluation of our manuscript and providing constructive feedback. We greatly appreciate the time and effort invested in reviewing our work. Following the reviewers' suggestions, we have carefully revised the manuscript and made the necessary modifications to address each comment in detail.

In the revised manuscript, all changes within the manuscript are highlighted in red for clarity. Below, we provide point-by-point responses to each reviewer's comment, along with supplementary results and detailed explanations of the revisions made.

COMMENTS TO THE AUTHOR

Reply to Reviewer #3

We sincerely thank the reviewer for their insightful comments and valuable suggestions, which have greatly improved the quality and rigor of our manuscript. We have carefully addressed each comment, and the detailed responses are as follows:

Reviewer point #4:

I do not agree that the current GSMR analysis indeed serves as "further causal validation." First of all, I cannot see exactly what the authors mean by "high-altitude adaptation leads to specific phenotypic outcome" in an evolutionary or statistical genetic model. Especially, in the light that the phenotypic outcomes (regression betas) are based on the modern Japanese population from BBJ. Second, MR is valid only under the assumption that the instrument variable (SNPs) must only affect the outcome through the exposure, not through any other pathway. If they insist HAA has to be the exposure, I don't think the authors can simply claim that SNPs affect outcomes (say WT) only through HAA. HAA is essentially Fst here, and it is not a measure of the selective benefit the SNP provides for high altitude adaptation. It's the overall fitness and can be low if it leads to other deleterious consequences, on top of adaptive benefit. Third, nominal p-values will not do here. It should be Bonferroni corrected. If this is not the area of expertise, perhaps this analysis should be dropped and the claim toned down regarding the target of selection. The paper would still be interesting to the readership.

Author response #4:

We thank the reviewer for the critical assessment of our GSMR analysis. After careful consideration of the methodological concerns raised, we have decided to remove the GSMR analysis and all related results from the manuscript for the following reasons:

1. Methodological Limitations: As correctly pointed out by the reviewer, the GSMR approach requires strict assumptions about instrument validity that may not be fully satisfied in our study design, particularly regarding the pleiotropic pathways through which genetic variants might influence high-altitude adaptation traits.

2. Statistical Rigor: We acknowledge that the nominal p-values used in our initial analysis were insufficient for establishing robust causal claims in this context.

Specific Changes Made:

We removed all GSMR results and interpretations from the Results section (formerly in the "Unraveling the complex physiological adaptations to high-altitude environments" subsection, Line 82-87), and deleted corresponding GSMR-related data (Supplementary Tables S3). Finally, we revised the Methods section to exclude the GSMR methodology description (Line 519-525).

Reviewer point #5:

In that case, the authors should not call it "heritability" or "heritability enrichment" because they are clearly not. To avoid misleading, I would suggest changing the term to something like "enrichment of high Fst" and clearly stating that the method (ldsc) is used outside the conventional context since Yang-GWAS is not based on a complex trait (it doesn't matter whether it's binary or quantitative).

Authors should mention the limitations of confounding due to stratification in the Discussion section.

Author response #5:

We thank the reviewer for this important comment regarding our use of the term “heritability enrichment.” In the revised manuscript, we have made the following clarifications and changes:

1. For the Yang-GWAS phenotype that reflects allele-frequency differentiation between Han and Tibetan populations, we now explicitly acknowledge that our LDSC-based enrichment analysis does not estimate heritability in the conventional sense. Instead, it effectively quantifies the enrichment of high Fst signals across genomic annotations. Accordingly, throughout the text we have replaced “heritability enrichment” with more precise terminology such as “enrichment of high Fst signals” for this phenotype, and we state that LDSC is here used outside its standard complex-trait heritability framework.

2. For the other high-altitude adaptation–related blood traits (e.g., hematological quantitative traits), the underlying GWAS are conventional complex-trait studies. In these cases, the LDSC analyses indeed estimate SNP-based heritability partitioned across eQTL annotations.

Therefore, for these blood traits we retain the term “heritability enrichment”, and we clarify this distinction in the Methods and Results sections.

3. We have systematically revised the description in the Results, Methods, and figure legends to (i) use “enrichment of high Fst” rather than “heritability enrichment” in the Yang-GWAS phenotype results, and (ii) reserve “heritability enrichment” exclusively for the LDSC analyses of standard blood-trait GWAS.

Modified Line 120-124: We found traits controlled by HAA-related loci (such as HCT, HGB, RBC) undergone significant heritability enrichment of eQTLs. Notably, these eQTLs show significant enrichment of high Fst variants, indicating strong population-specific selection in NHs. Furthermore, compared with cis-eQTLs, trans-eQTLs demonstrate a substantially greater enrichment of high Fst variants linked to NHs HAA phenotypes, with an enrichment fold (Pr(H2g)/Pr(SNPs)) of 48.28.

Modified Legend of Figure 2H: LD score regression results showing heritability partitioned enrichments of cis-eQTL and trans-eQTL annotations across multiple high-altitude adaptation (HAA)-related trait GWAS. For the HAA phenotype, this analysis captures enrichment of high Fst signals rather than conventional heritability.

Added Line 505-507: In the case of the Yang GWAS, LDSC was applied beyond its conventional complex trait heritability framework, and the apparent eQTL heritability enrichment in fact reflected enrichment of high Fst eQTL signals.

4. We have added the limitations of confounding due to stratification in the Discussion section.

Added Line 390-393: Secondly, our study is subject to potential confounding from both population stratification and local LD structure. In addition to differences in high-altitude adaptation status, the AN and NH populations may have inherent differences in genetic background that are not fully captured even after PCA-based correction for population stratification.

Reviewer point #6:

FDR is 1.0 for most of the signals in Table S7. Analyses based on these signals are not acceptable.

Author response #6:

We thank the reviewer for carefully examining Table S7. In the revised manuscript, we re-checked all the results in Table S7 and applied a stringent threshold of FDR < 0.05. All signals with FDR ≥ 0.05 (including those with FDR = 1.0) have been removed and are no longer used in any subsequent analyses, figures, or interpretations (Supplementary Fig S6).

Modified Line 158-168: Utilizing Decon-QTL, we identified 130 cis-ieQTLs (FDR < 0.05). Within these cell type ieQTLs, 12 ieGenes were recognized. The most substantial subset of interactions was observed with IgD+ IgM- B cells, comprising 89 ieQTLs (68.5%), which also represented the most divergent cell fraction (Supplementary Table S6). These ieQTLs provide valuable insights into cell type-specific genetic factors associated with HAA. For instance, the *HBD* gene, which encode essential structural subunits of hemoglobin, were transcriptionally upregulated by the rs117543228 variant in HLA-DR+ regulatory T cells (FDR = 5.23e-12), suggesting a potential mechanism of high-altitude adaptation through enhanced hemoglobin synthesis and consequently improved systemic oxygen transport under chronic hypoxia. We further identified that rs201299572 upregulates *NCOA4* expression in HLA-DR+ regulatory T cells (FDR = 3.59e-7), which may support efficient erythropoiesis through modulating erythroid differentiation, thereby potentially contributing to high-altitude adaptation.

Modified Line 465: Significant ieQTLs were identified using the threshold of FDR < 0.05.

Reviewer point #7:

There are still other places overstating the reliability of causality tests.

Lines 49-51: Combined with causal inference methods such as transcriptome-wide association studies (TWAS), Mendelian randomization (MR), and genetic co-localization (coloc) analyses, eQTL analysis can identify authentic causal variants and establish causal link of “variant-gene-phenotype”.

Lines 65-67: Utilizing causal inference techniques such as Mendelian randomization, colocalization, and TWAS, we identified the causal variants and regulatory genes linked to blood physiological traits associated with HAA.

Author response #7:

We acknowledge the overstatement of causal claims and have comprehensively revised the manuscript to weaken absolute causal expressions.

Modified Lines 49-51: Combined with causal inference methods such as transcriptome-wide association studies (TWAS), Mendelian randomization (MR), and genetic co-localization (coloc) analyses, eQTL analysis can identify potential causal variants and infer the regulatory link of “variant-gene-phenotype”.

Modified Lines 65-68: Utilizing causal inference techniques such as Mendelian randomization, colocalization, and TWAS, we prioritized potential causal variants and candidate regulatory genes linked to blood physiological traits associated with HAA.

Reviewer point #8:

I think the authors are misunderstanding my point on LD confounding. LD pruning can't address this question. Actually, LD pruning makes false positives more likely. I was suggesting a Credible SNP analysis for eQTL data. However, note that you cannot run credible SNP analyses on Yang GWAS since this is not really a GWAS. That said, perhaps in some cases, you may be able to find some compelling cases (without LD pruning), suggesting the shared causal variant is very convincing between traits.

Author response #8:

We appreciate the reviewer's guidance on LD confounding and Credible SNP analysis. Given that this comment initially targeted trans-eQTLs in the first round, we performed SuSiE-RSS fine-mapping of trans-eQTL loci in the two canonical high-altitude adaptation genes, *EPAS1* and *EGLN1*, to identify credible SNPs (Table1).

For *EGLN1*, we observed that credible SNPs (rs2808611, rs2491416) overlapped with variants associated with hemoglobin (Hb), hematocrit (HCT), and red blood cell (RBC) traits; however, these credible SNPs did not show detectable trans-regulatory effects on gene expression. In contrast, the trans-eQTL variants (rs12097901) that did exhibit regulatory effects in our data had posterior inclusion probabilities (PIPs) of 0 in the fine-mapping results. For *EPAS1*, the credible SNPs did not overlap with credible SNPs associated with high-altitude related blood phenotypes. The trans-eQTL variants (rs72797404) with regulatory effects at the *EPAS1* locus also had PIP = 0, although additional credible regulatory SNPs (rs4952819, rs7582701, rs11125071, rs13420272, rs4953346) were present within a 200kb window, suggesting that these signals may be influenced by linkage disequilibrium (LD) confounding.

Taken together, these findings indicate that we cannot fully exclude the impact of LD confounding on our trans-eQTL results. Accordingly, in the interpretation of our results we treat the inferred causal trans-eQTL effects as tentative candidates rather than definitive causal variants, and we have explicitly added this limitation regarding potential LD confounding to the Discussion section.

Modified Line 212-221: Among these 181 potential trans-causal variants, 47 are adaptive genetic variants specific to NHs population (Fst > 0.2), 11 of which are situated in genomic regions that are well recognized as NHs-specific (Table 1), 94 of which are situated in regulatory regions (Supplementary Table 10). Notably, eSNPs within *EPAS1* locus showed a significant correlation with the expression levels of *PURB*, which implicated in the regulation of hemopoiesis (Supplementary Fig 6B). Additionally, eSNPs at *EGLN1* locus were found to be in association with the expression of *PDE8A*, a gene pivotal for cardiac function (Supplementary Fig S6C). Furthermore, eSNPs within *CRIPT* locus demonstrated a robust

association with genes implicated in the Toll-like receptor signaling pathway (*TLR1*), pulmonary hypertension regulation (*MAGED1*), and the regulation of cAMP-dependent protein kinase activity (*PKIG*) (Supplementary Fig 6D).

Added Line 391-393: Moreover, although our causal analysis identified several loci with apparent trans-eQTL effects, the extensive LD in these regions means that we cannot exclude the possibility that the observed associations are driven by LD confounding rather than direct regulatory effects.

Table 1 Credible SNPs of EPAS1 and EGLN1

snp_id	CHR	BP	PIP	is_trans_eQTL	Phenotype	Gene
rs3754569	2	46125314	1	No	Hb	EPAS1
rs4953318	2	46127912	1	No	Hb	EPAS1
rs10168349	2	46133768	1	No	Hb	EPAS1
rs13410378	2	46136668	1	No	Hb	EPAS1
rs3754568	2	46144075	1	No	Hb	EPAS1
rs3754569	2	46125314	1	No	RBC	EPAS1
rs4953318	2	46127912	1	No	RBC	EPAS1
rs10168349	2	46133768	1	No	RBC	EPAS1
rs13410378	2	46136668	1	No	RBC	EPAS1
rs3754568	2	46144075	1	No	RBC	EPAS1
rs3754569	2	46125314	1	No	HCT	EPAS1
rs4953318	2	46127912	1	No	HCT	EPAS1
rs10168349	2	46133768	1	No	HCT	EPAS1
rs13410378	2	46136668	1	No	HCT	EPAS1
rs3754568	2	46144075	1	No	HCT	EPAS1
rs2034327	2	46321901	1	No	trans_rs72797404	EPAS1
rs4952819	2	46323579	1	Yes	trans_rs72797404	EPAS1
rs7582701	2	46323630	1	Yes	trans_rs72797404	EPAS1
rs4952820	2	46326827	1	No	trans_rs72797404	EPAS1
rs11125071	2	46327394	1	Yes	trans_rs72797404	EPAS1
rs13420272	2	46329684	1	Yes	trans_rs72797404	EPAS1
rs13382833	2	46329690	1	No	trans_rs72797404	EPAS1
rs11900910	2	46330461	1	No	trans_rs72797404	EPAS1
rs4953346	2	46331069	1	Yes	trans_rs72797404	EPAS1
rs72797404	2	46351259	0	Yes	trans_rs72797404	EPAS1
rs2066140	1	231368565	1	No	Hb	EGLN1

rs2808611	1	231412734	1	No	Hb	EGLN1
rs186996510	1	231421877	0.9999	No	Hb	EGLN1
rs2491416	1	231411589	1	No	RBC	EGLN1
rs2808611	1	231412734	1	No	RBC	EGLN1
rs186996510	1	231421877	0.9999	No	RBC	EGLN1
rs2066140	1	231368565	1	No	HCT	EGLN1
rs2790877	1	231399681	0.9961	No	HCT	EGLN1
rs2808608	1	231407429	0.9162	No	HCT	EGLN1
rs2790867	1	231412313	0.9866	No	HCT	EGLN1
rs2808611	1	231412734	1	No	HCT	EGLN1
rs2248646	1	231392211	0.9957	No	trans_rs12097901	EGLN1
rs2790870	1	231408077	1	No	trans_rs12097901	EGLN1
rs2491416	1	231411589	1	No	trans_rs12097901	EGLN1
rs2808611	1	231412734	1	No	trans_rs12097901	EGLN1
rs2808616	1	231419247	0.9949	No	trans_rs12097901	EGLN1
rs12097901	1	231421509	0	Yes	trans_rs12097901	EGLN1

Reviewer point #10:

I believe that the standard practice of resource paper is to release all cis-eQTL *genome-wide* regardless of their p-values at least. I understand this can be too onerous for trans-eQTLs.

Author response #10:

Thanks for your valuable advice. We have now deposited genome-wide summary statistics for all cis-eQTLs in a publicly accessible repository on National Genomics Data Center (NGDC). The data are openly available in OMIX at NGDC under accession number OMIX014471. Open review link is https://ngdc.cncb.ac.cn/omix/preview/gfFhv2EW.

Modified Lines 583-586: The raw Genotype data and RNA-seq data have been deposited in the National Genomics Data Center (NGDC) under the accession number HRA009343, and the eQTL summary data are available at the same repository with the accession number OMIX014471 (https://ngdc.cncb.ac.cn/).
