# Supplementary material for: Identifying causal genetic variants for high-altitude adaptation through blood eQTL analysis in plateau populations
Source: Commun Biol. 2026 Aug 26;9:1186. doi: 10.1038/s42003-026-10840-6 (PMC13558674; doi:10.1038/s42003-026-10840-6)
Supplement: Supplementary file 2 [file 42003_2026_10840_MOESM2_ESM.pdf]

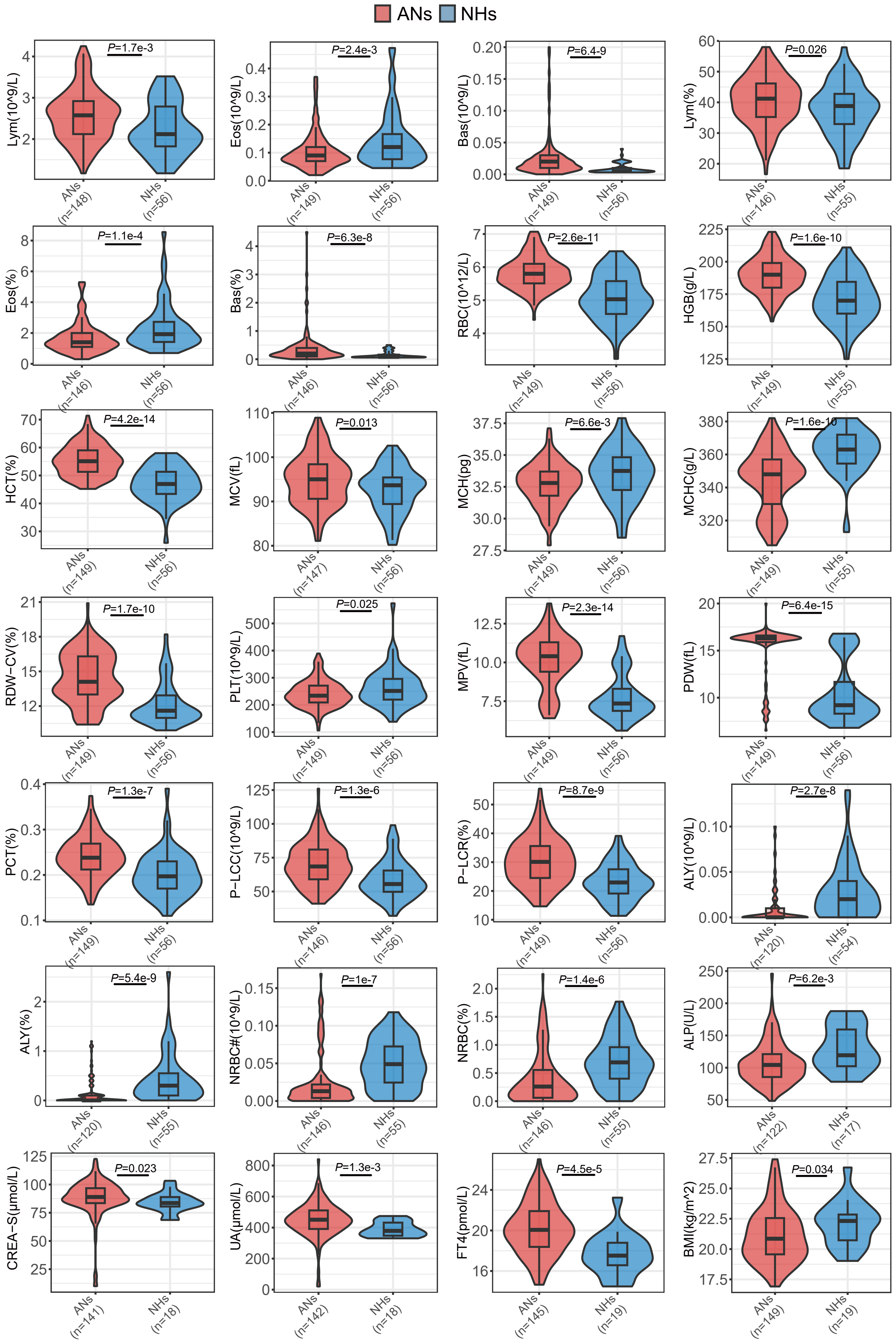

Supplementary Fig. S1 Difference of blood phenotypes between NHs and ANs in high altitude.

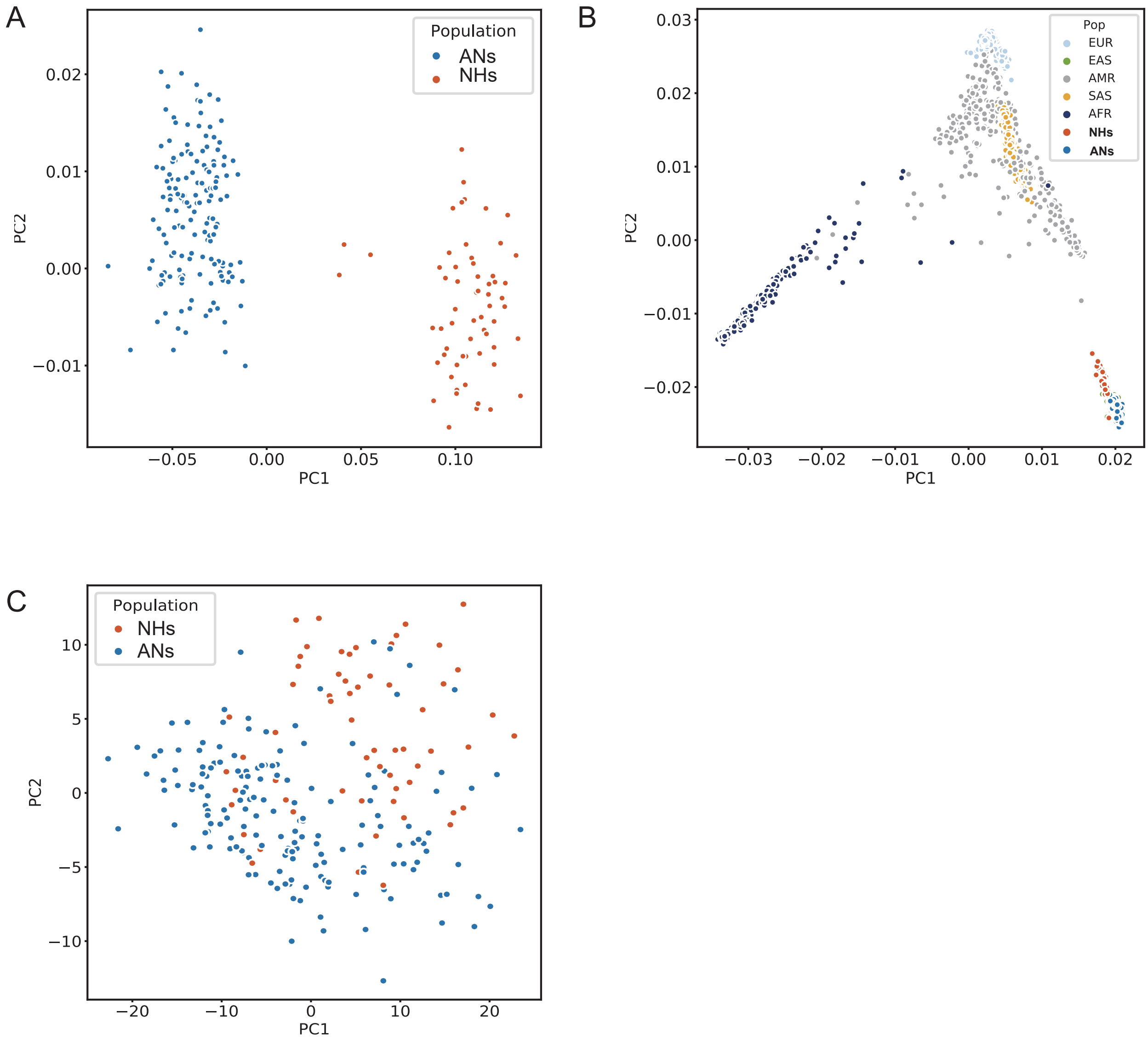

Supplementary Fig. S2 (A) PCA of genomics in NHs and ANs. (B) PCA of genomics in 1000 genomes. (C) PCA of differential expression genes in NHs and ANs.

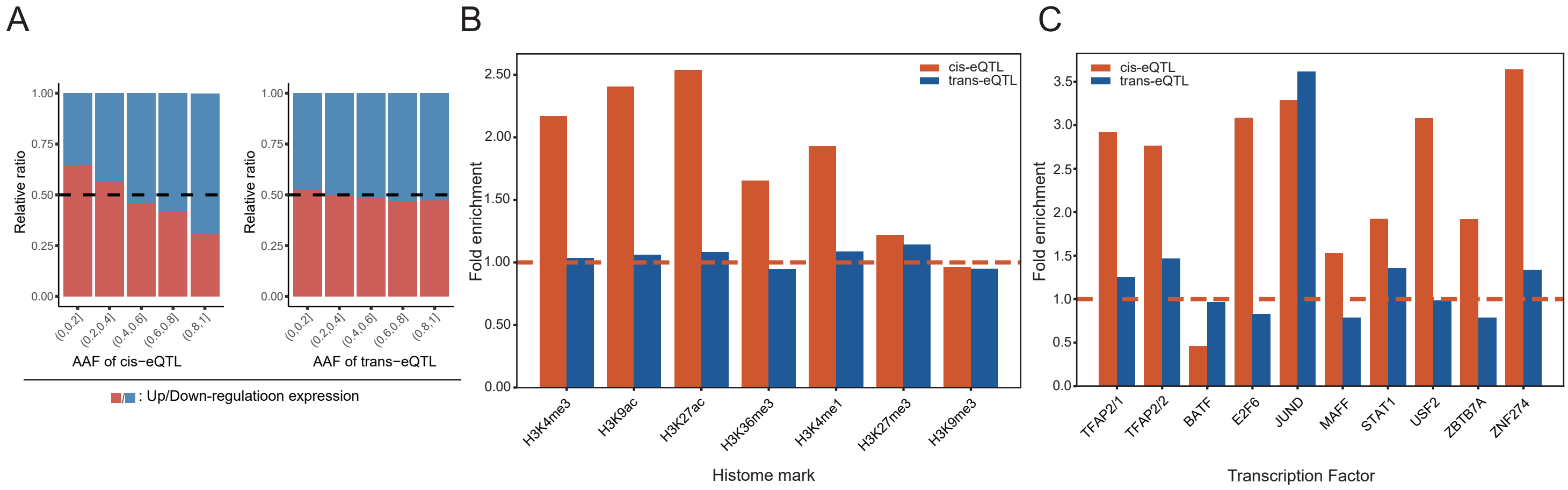

Supplementary Fig. S3 (A) The bar plot represents the proportion of cis-eQTLs and trans-eQTLs in each AAF interval. (B) Enrichment of eQTLs in Histone annotation data from REMC. (C) Enrichment of eQTLs in transcript factors.

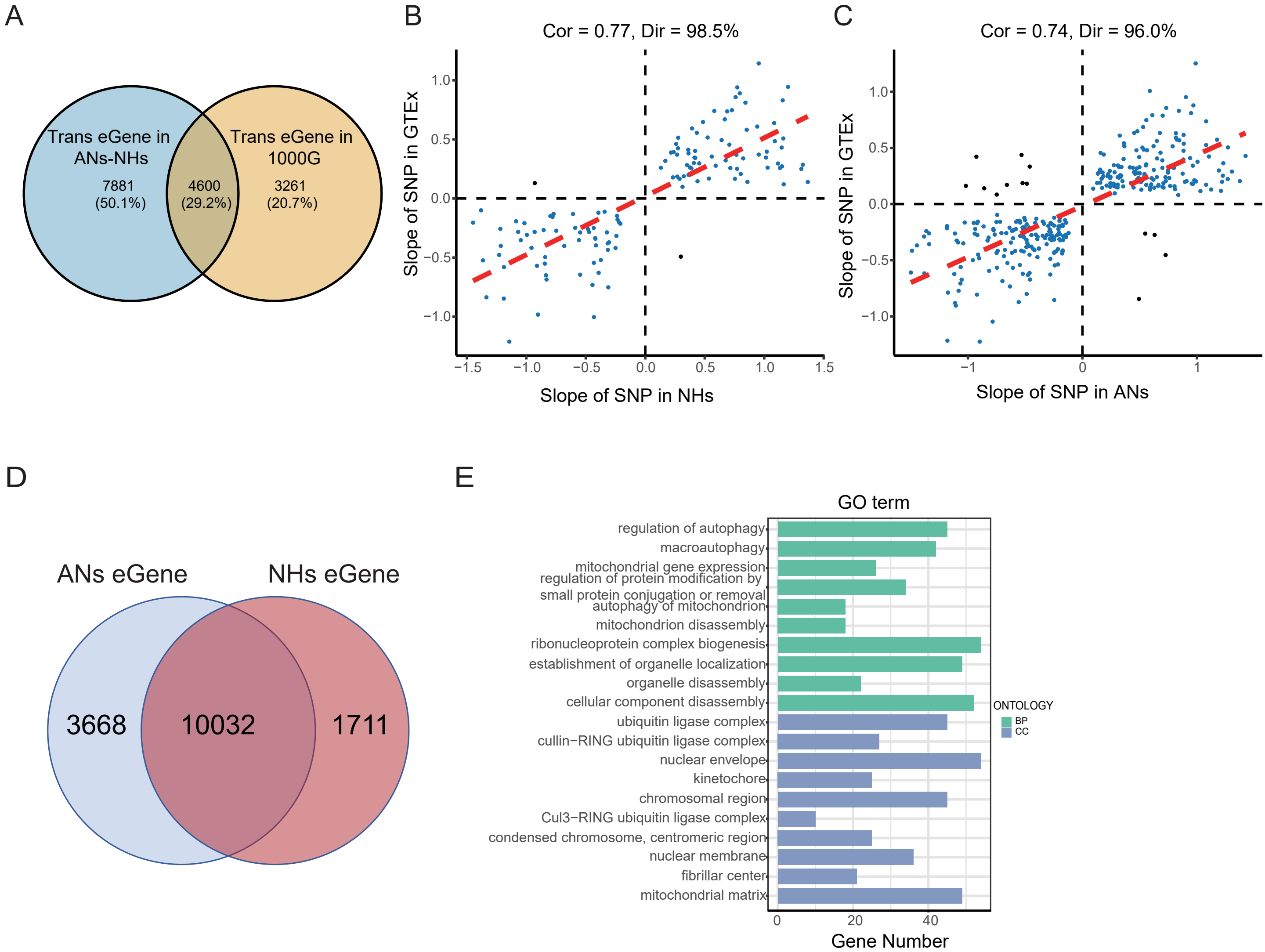

Supplementary Fig. S4 (A) Venn diagram of trans eGens between ANs-NHs and eQTLGen. (B, C) Comparison of effect sizes from significant cis-eQTLs in NHs (B), ANs (C) and GTEx whole blood tissues (FDR < 0.05). (D) Venn diagram of cis-, trans-eGenes between ANs and NHs. (E) NHs specific eGenes GO Enrichments.

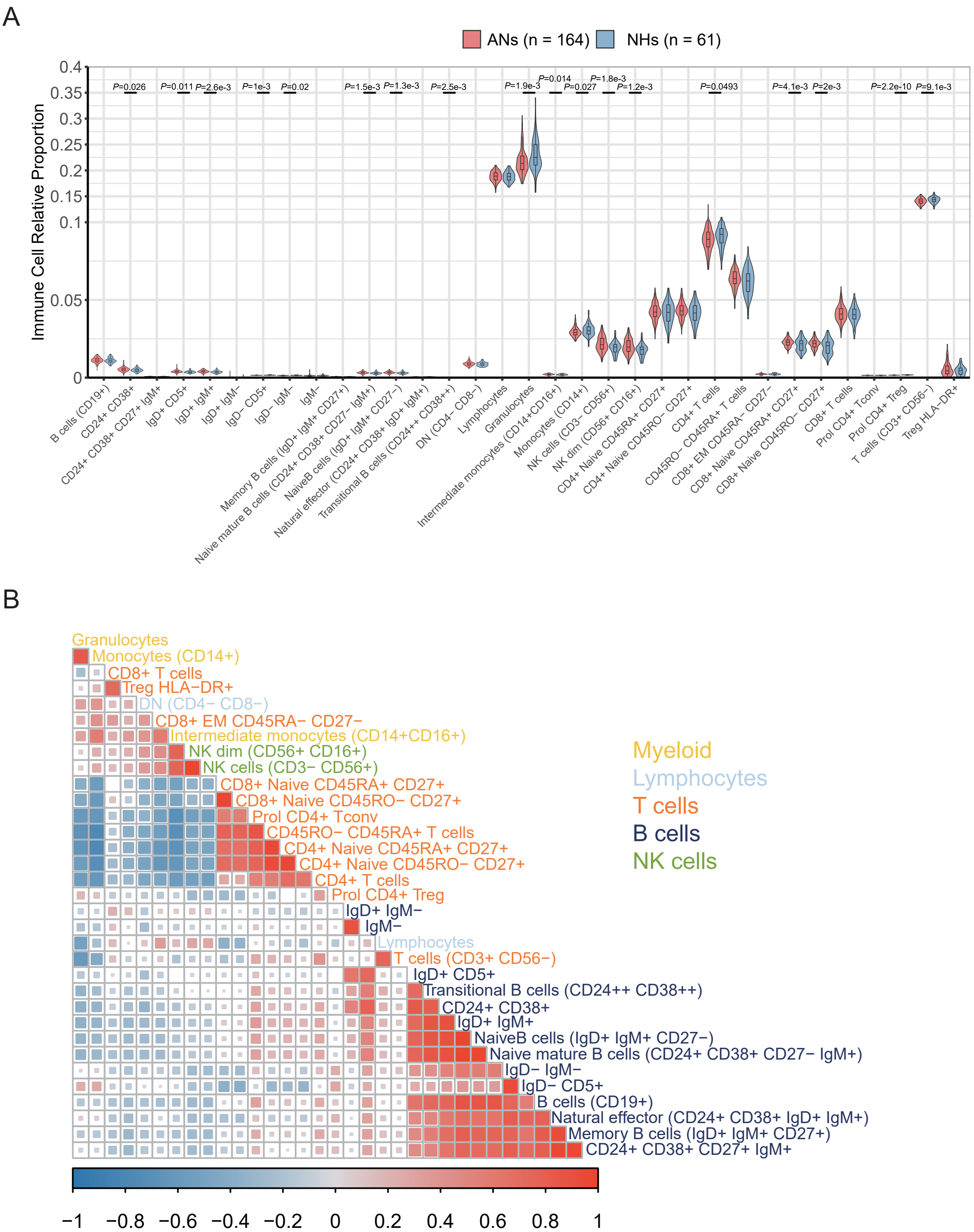

Supplementary Fig. S5 (A) Cell proportion difference between ANs and NHs. (B) Correlation analysis between diferent cell types.

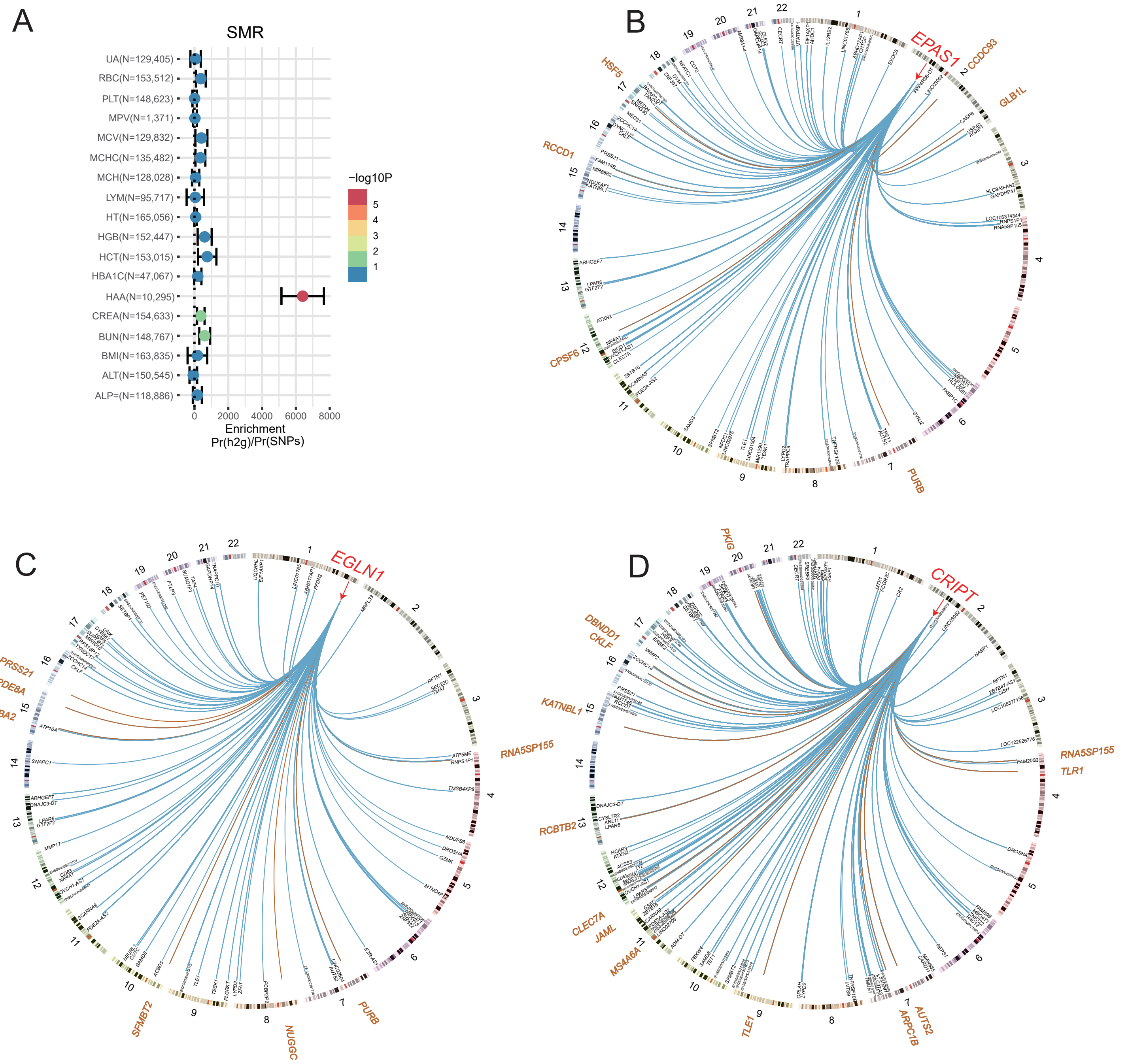

Supplementary Fig. S6 (A) LD score regression partitioned heritability enrichments for SMR eQTLs annotations across multiple HAA GWAS. Enrichments are colored by -log10(P) and error bars represent SE. (B-D) Circos plot of autosomes indicating the regulated genes by the *EPAS1* (B), *EGLN1* (C), and *CRIPT* (D) locus.

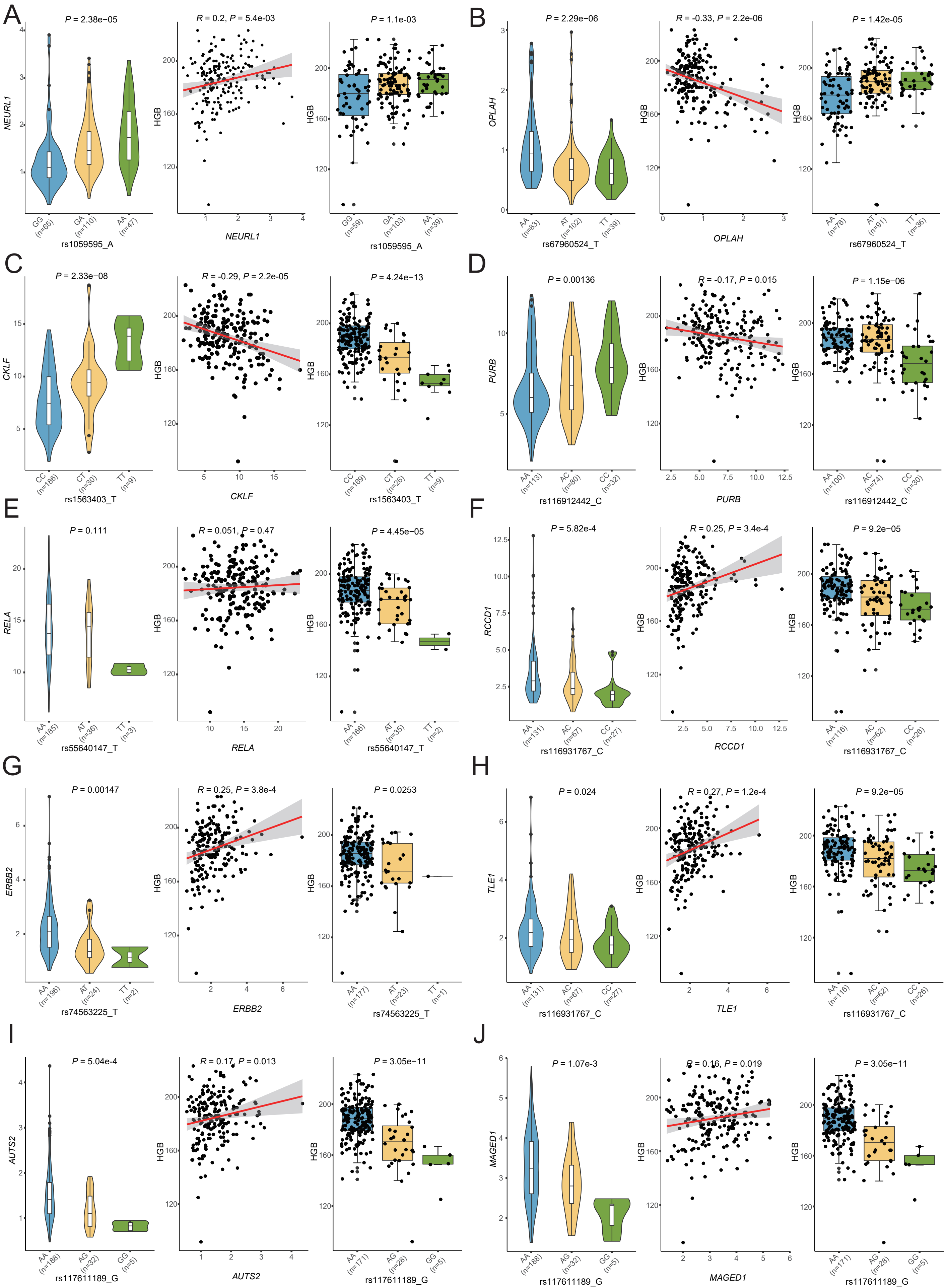
A
P = 2.38e−05
NEURL1
GG (n=65)
GA (n=110)
AA (n=47)
rs1059595_A
R = 0.2, P = 5.4e-03
HGB
NEURL1
P = 1.1e-03
HGB
GG (n=59)
GA (n=103)
AA (n=39)
rs1059595_A
B
P = 2.29e−06
OPLAH
AA (n=83)
AT (n=102)
TT (n=39)
rs67960524_T
R = -0.33, P = 2.2e-06
HGB
OPLAH
P = 1.42e-05
HGB
AA (n=76)
AT (n=91)
TT (n=36)
rs67960524_T
C
P = 2.33e−08
CKLF
CC (n=186)
CT (n=30)
TT (n=9)
rs1563403_T
R = -0.29, P = 2.2e-05
HGB
CKLF
P = 4.24e-13
HGB
CC (n=169)
CT (n=26)
TT (n=9)
rs1563403_T
D
P = 0.00136
PURB
AA (n=113)
AC (n=80)
CC (n=32)
rs116912442_C
R = -0.17, P = 0.015
HGB
PURB
P = 1.15e-06
HGB
AA (n=100)
AC (n=74)
CC (n=30)
rs116912442_C
E
P = 0.111
RELA
AA (n=185)
AT (n=36)
TT (n=3)
rs55640147_T
R = 0.051, P = 0.47
HGB
RELA
P = 4.45e-05
HGB
AA (n=166)
AT (n=35)
TT (n=2)
rs55640147_T
F
P = 5.82e-4
RCCD1
AA (n=131)
AC (n=67)
CC (n=27)
rs116931767_C
R = 0.25, P = 3.4e-4
HGB
RCCD1
P = 9.2e-05
HGB
AA (n=116)
AC (n=62)
CC (n=26)
rs116931767_C
G
P = 0.00147
ERBB2
AA (n=196)
AT (n=24)
TT (n=2)
rs74563225_T
R = 0.25, P = 3.8e-4
HGB
ERBB2
P = 0.0253
HGB
AA (n=177)
AT (n=23)
TT (n=1)
rs74563225_T
H
P = 0.024
TLE1
AA (n=131)
AC (n=67)
CC (n=27)
rs116931767_C
R = 0.27, P = 1.2e-4
HGB
TLE1
P = 9.2e-05
HGB
AA (n=116)
AC (n=62)
CC (n=26)
rs116931767_C
I
P = 5.04e-4
AUTS2
AA (n=188)
AG (n=32)
GG (n=5)
rs117611189_G
R = 0.17, P = 0.013
HGB
AUTS2
P = 3.05e-11
HGB
AA (n=171)
AG (n=28)
GG (n=5)
rs117611189_G
J
P = 1.07e-3
MAGED1
AA (n=188)
AG (n=32)
GG (n=5)
rs117611189_G
R = 0.16, P = 0.019
HGB
MAGED1
P = 3.05e-11
HGB
AA (n=171)
AG (n=28)
GG (n=5)
rs117611189_G

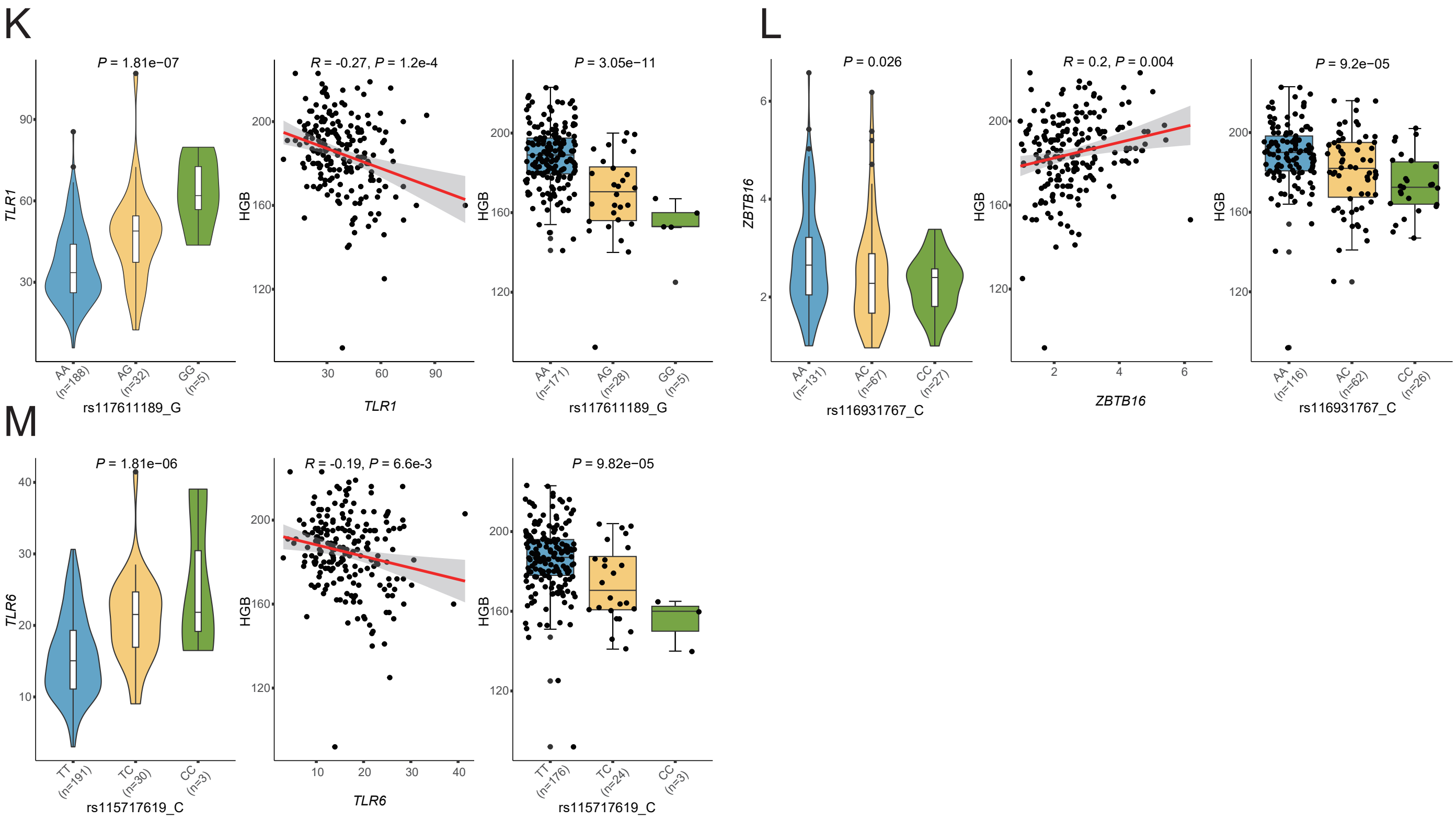

Supplementary Fig. S7 (A-M) *NEURL1, OPLAH*, *CKLF, PURB, RELA, RCCD1, ERBB2, TLE1, AUTS2, MAGED1, TLR1, ZBTB16, TLR6, related to HGB.*

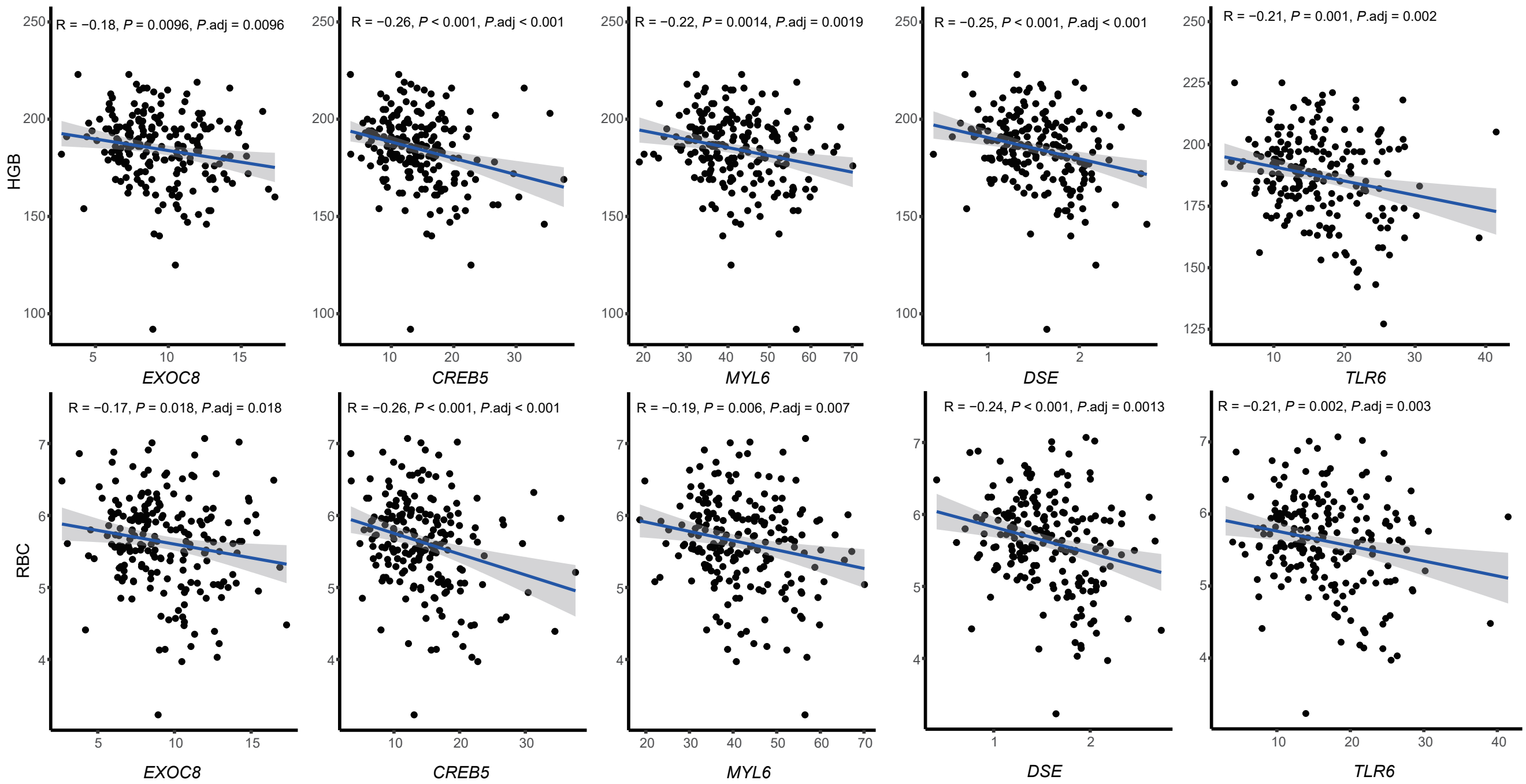

Supplementary Fig. S8 Scatter plots showing the associations between HGB, RBC and five TWAS significant genes. The grey shade area represents 95% confidence interval. *P*-adjust represents the BH-adjusted *P* values of Pearson's correlation test.

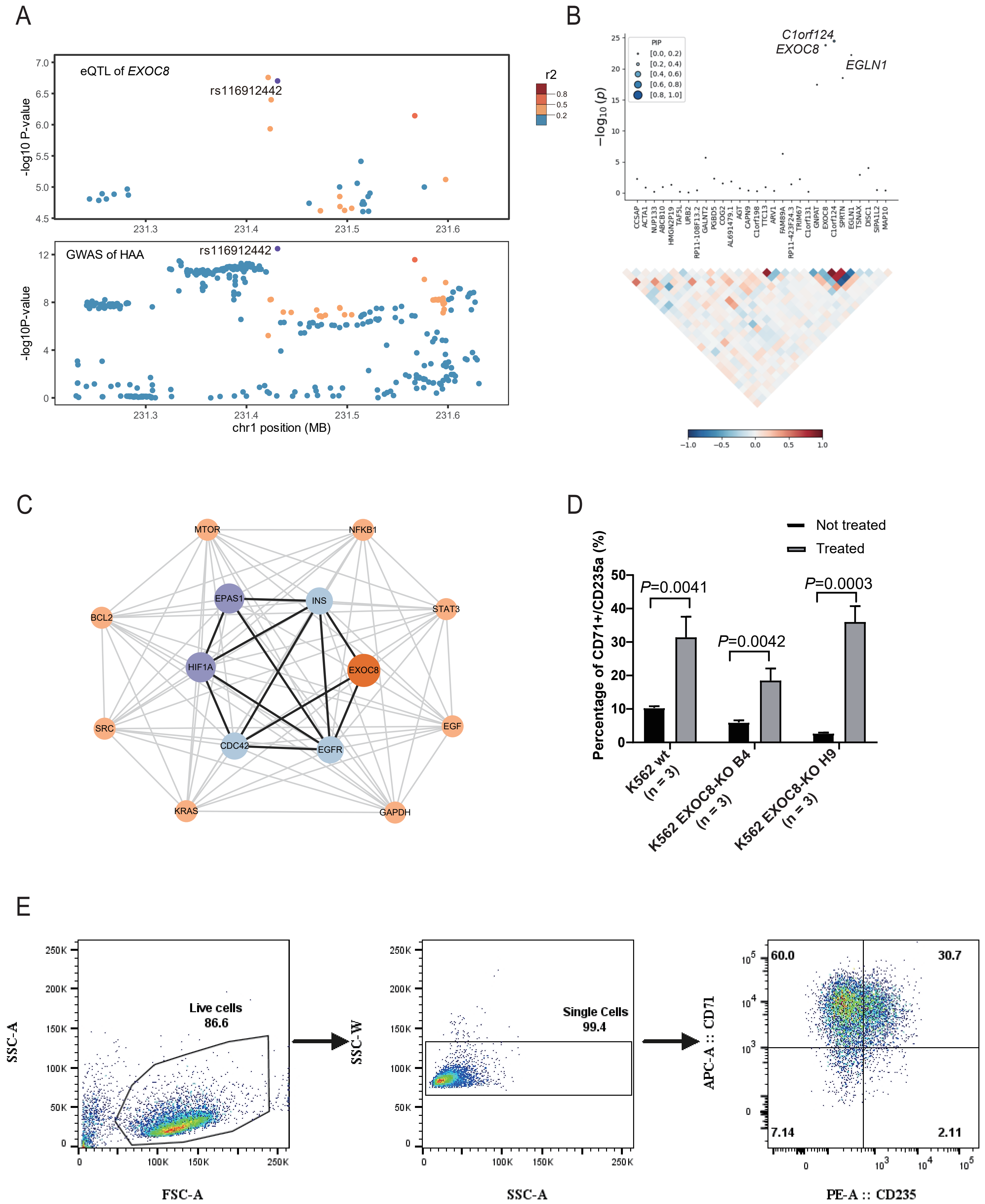

A
eQTL of EXOC8
rs116912442
-log10 P-value
GWAS of HAA
-log10P-value
chr1 position (MB)
r2
B
C1orf124
EXOC8
EGLN1
PIP
C
MTOR
NFKB1
EPAS1
INS
BCL2
STAT3
HIF1A
EXOC8
SRC
EGF
CDC42
EGFR
KRAS
GAPDH
D
Not treated
Treated
Percentage of CD71+/CD235a (%)
P=0.0041
P=0.0042
P=0.0003
K562 wt (n = 3)
K562 EXOC8-KO B4 (n = 3)
K562 EXOC8-KO H9 (n = 3)
E
Live cells 86.6
SSC-A
FSC-A
Single Cells 99.4
SSC-W
SSC-A
APC-A :: CD71
PE-A :: CD235
60.0
30.7
7.14
2.11

F

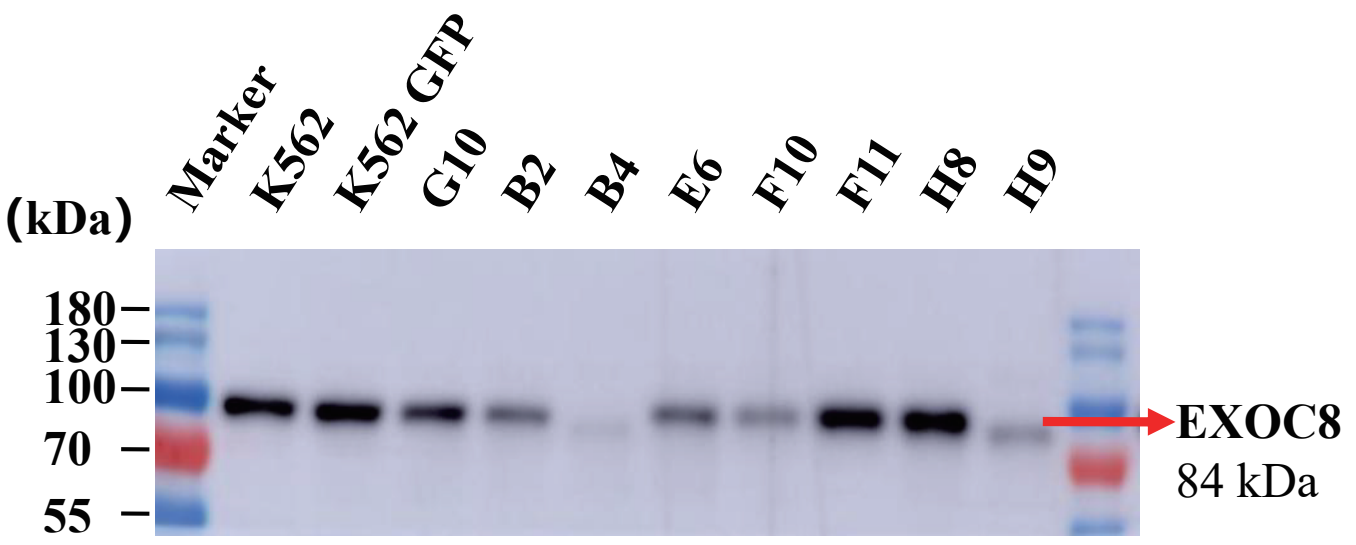

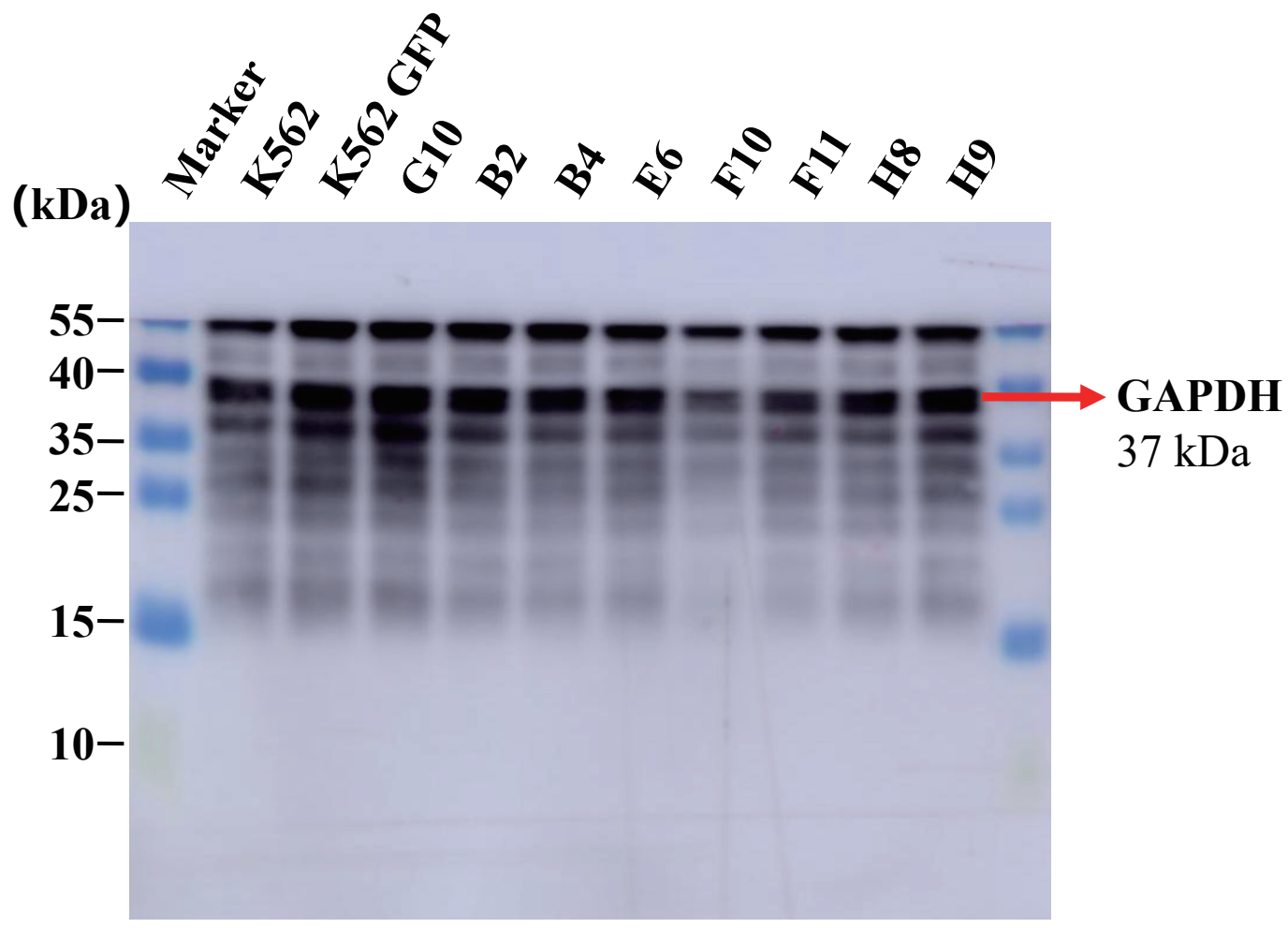

Supplementary Fig. S9 (A) Regional scatter plots of eQTLs for *EXOC8* (top) and GWAS for HAA (bottom) in a 300-kb region centered on rs116912442. (B) Fine mapping TWAS results for HAA. (C) Core PPI network of EXOC8. (D) Histogram analysis of erythroid differentiation in different K562 cell groups. The percentage of the CD235a+/CD71+ populations with or without heminand Ara-C treatment is indicated in vertical axis. Results represent the mean±SD from three independent experiments ($P < 0.05$). (E) The gating strategy of flow cytometry for the analysis of erythroid differentiation in K562 cells. The gating strategy was as follows: Live cells were first gated on FSC/SSC.Then the single cells were gated using SSC-A/SSC-H. The cell populations were then analysed using the CD235a+/CD71+ gate clustering method for the assessment of the erythroid differentiation.
(F) Uncropped and unedited western blot images for EXOC8 knockout validation.

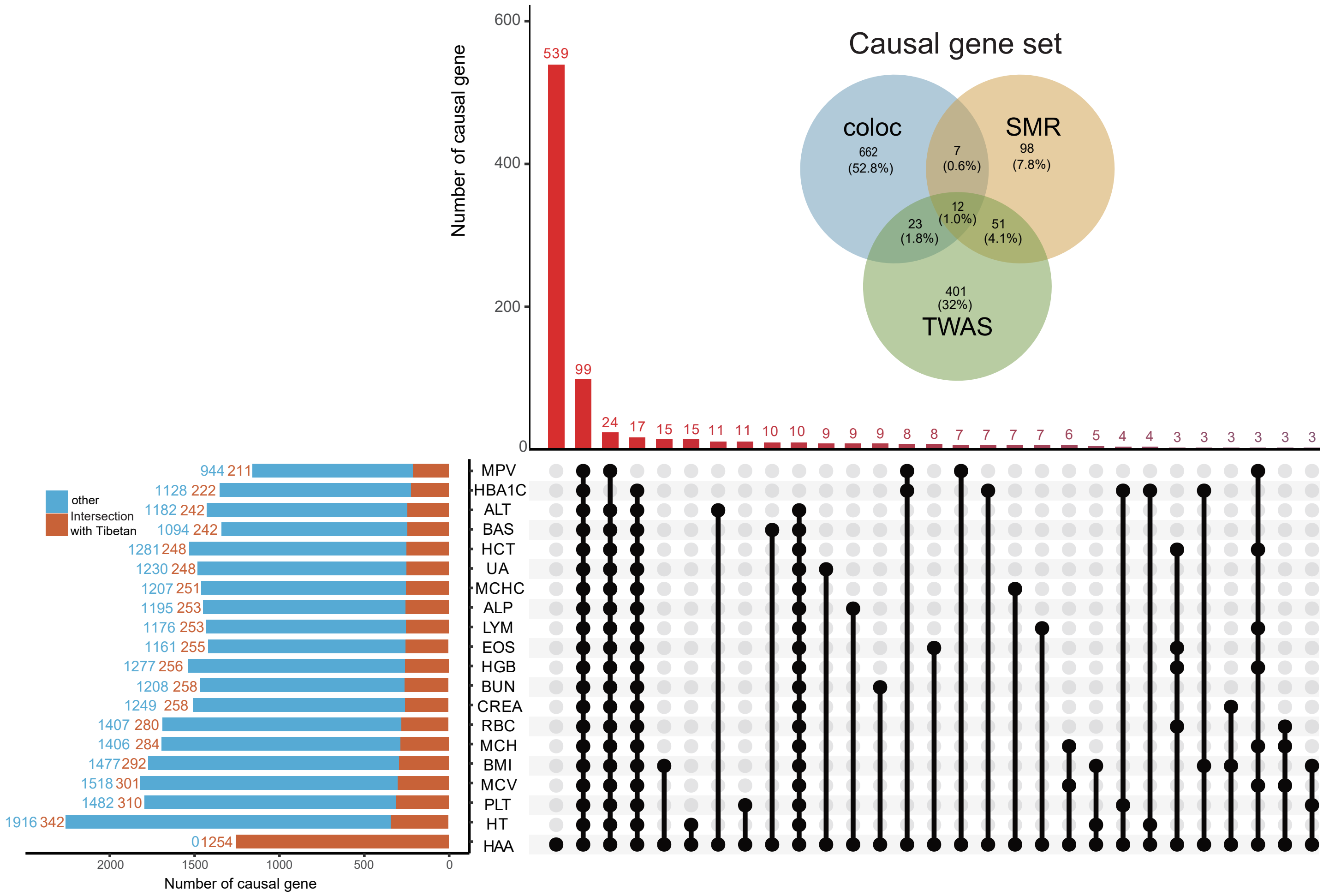

Supplementary Fig. S10 Intersection of causal gene sets from 19 related physiological phenotypes with NHs. The causal gene sets are identified by SMR, coloc, and TWAS.

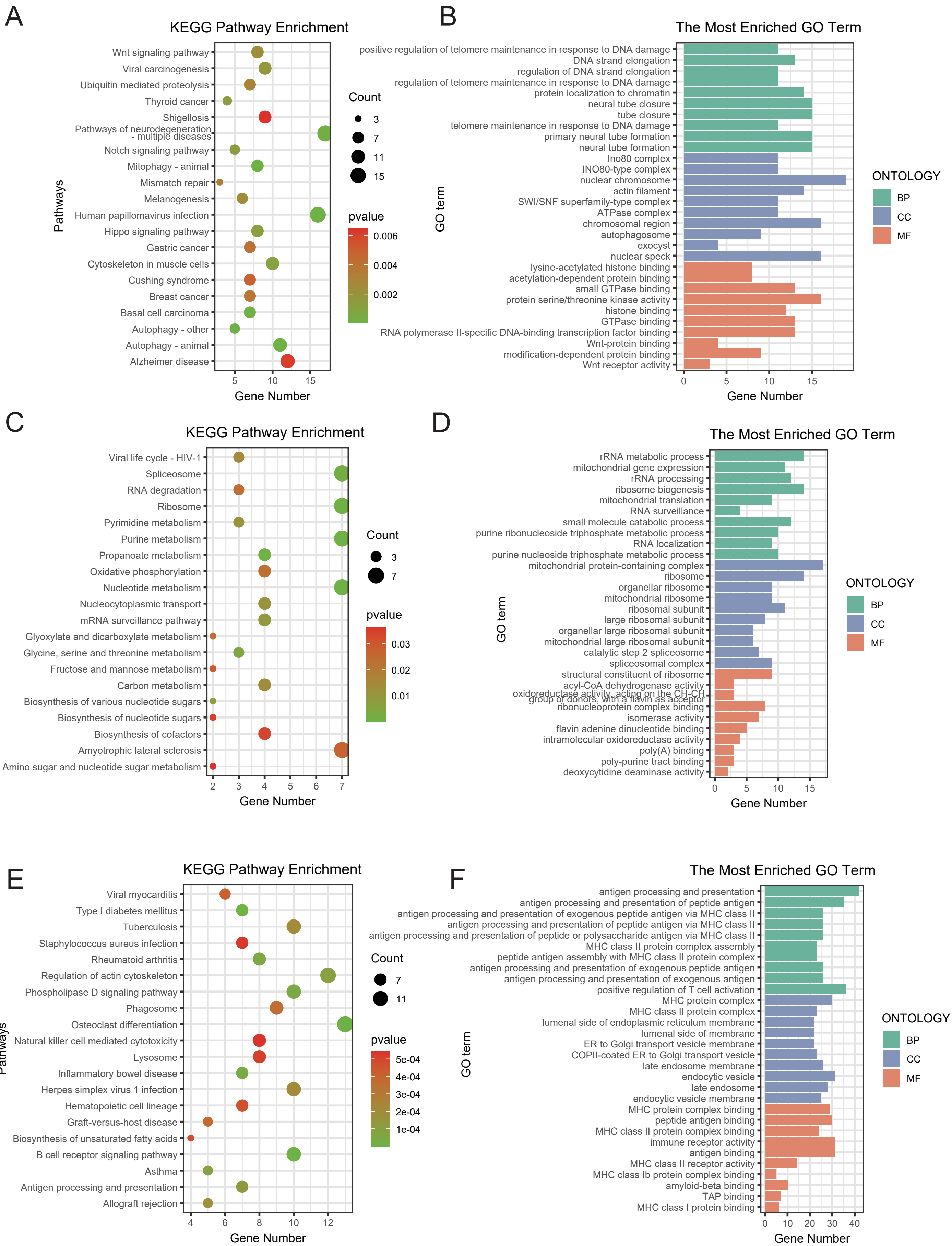

Supplementary Fig. S11 (A, B) KEGG and GO Enrichments of submodule1. (C, D) KEGG and GO Enrichments of submodule2. (E, F) KEGG and GO Enrichments of submodule3.

A

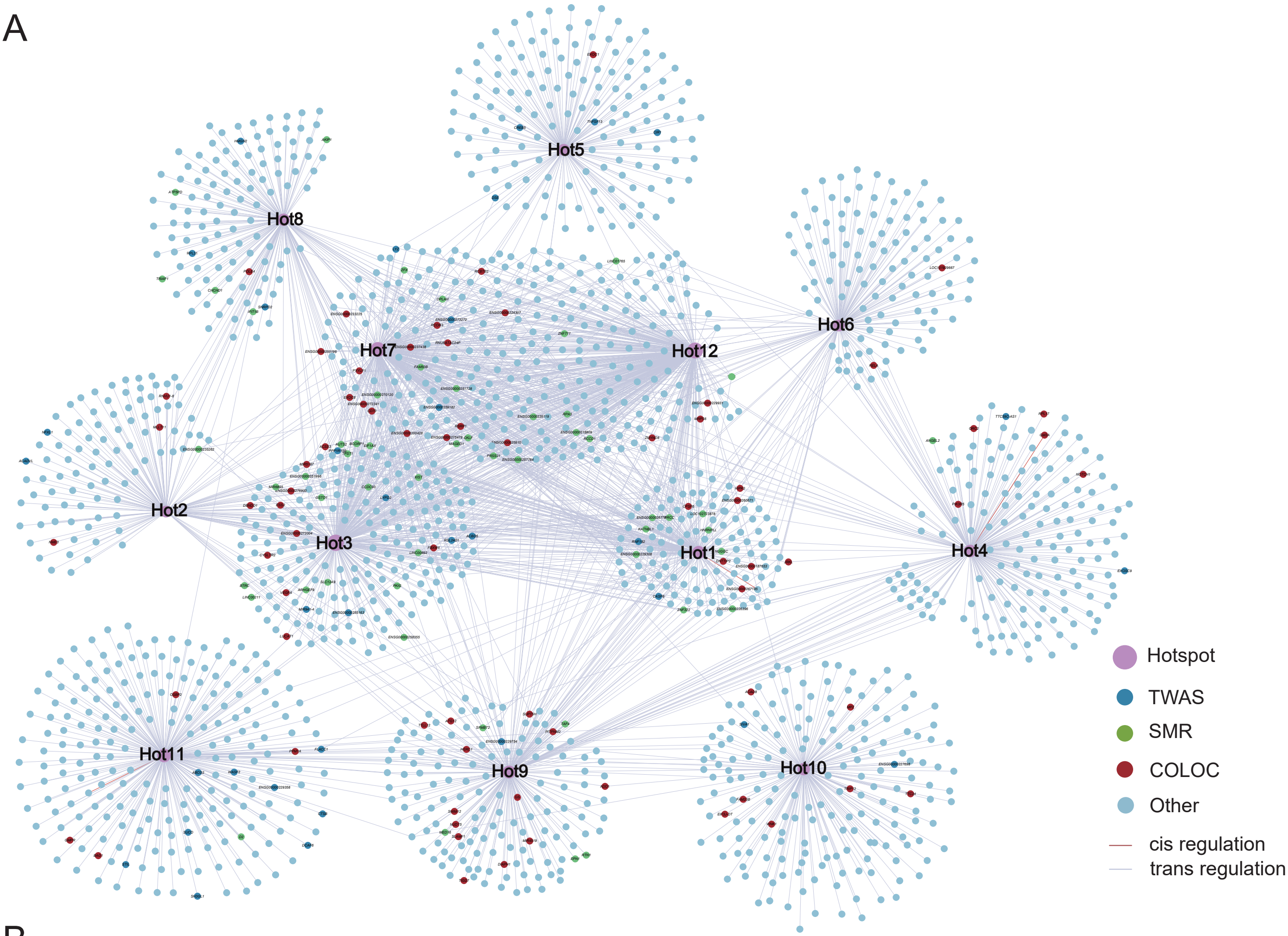

B

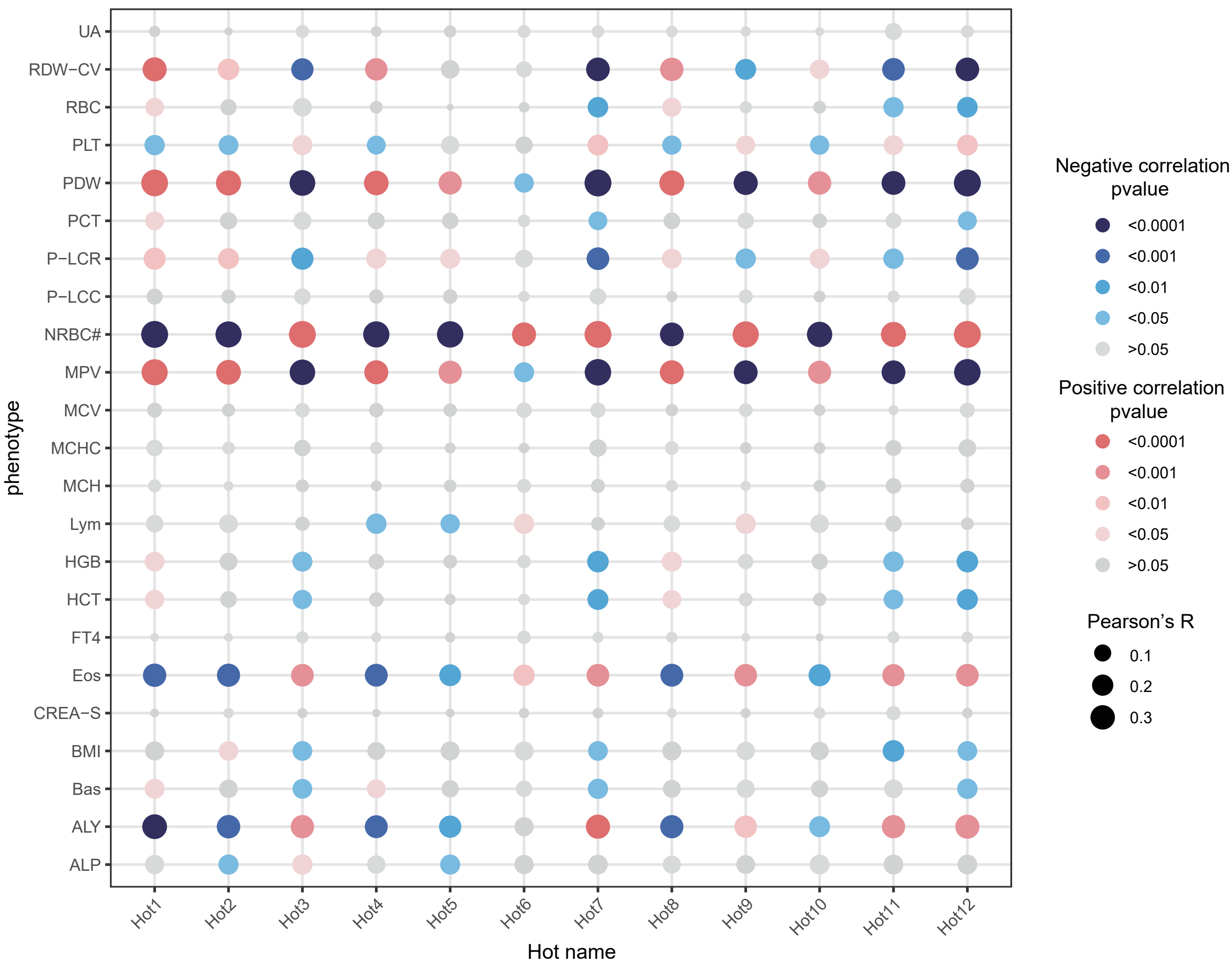

Supplementary Fig. S12 (A) eQTL Hotspot network diagram. (B) Heatmap of hotspot-phenotype correlations

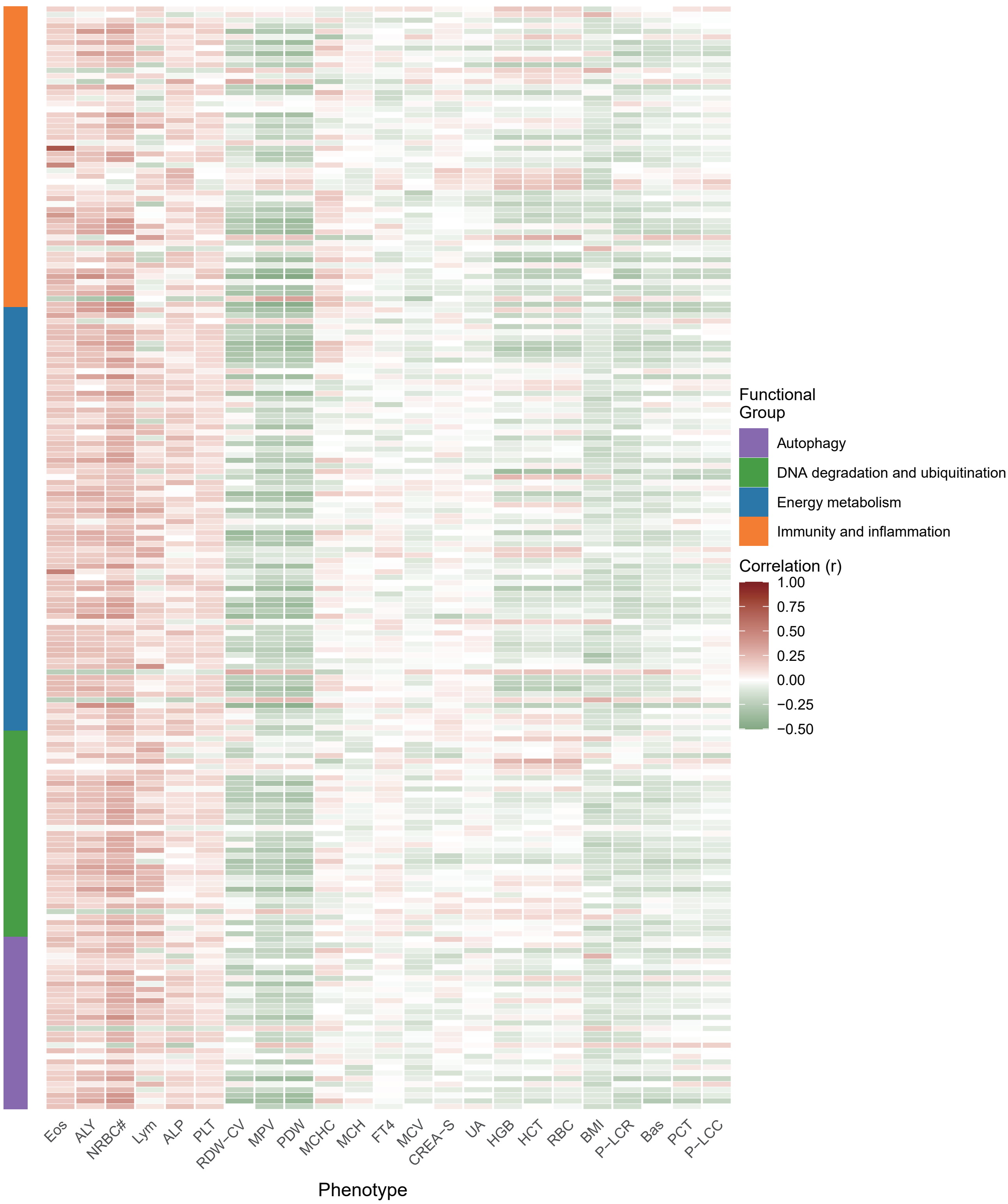

Supplementary Fig. S13 Correlation heatmap between 23 differential phenotypes and genes from 4 modules.
