# Supplementary material for: Identifying causal genetic variants for high-altitude adaptation through blood eQTL analysis in plateau populations
Source: Commun Biol. 2026 Aug 26;9:1186. doi: 10.1038/s42003-026-10840-6 (PMC13558674; doi:10.1038/s42003-026-10840-6)
Supplement: Supplementary file 3 [file 42003_2026_10840_MOESM3_ESM.pdf]

Description of Additional Supplementary Files

File name: Supplementary Data 1

Description: We have provided the numerical source data underlying the graphs and charts in the main figures as Supplementary Data files in Excel format. These files have been uploaded with the revised manuscript.

File name: Supplementary table 1-18

Description: All the supplementary table attached in one single file.
