# Supplementary material for: Identifying causal genetic variants for high-altitude adaptation through blood eQTL analysis in plateau populations
Source: Commun Biol. 2026 Aug 26;9:1186. doi: 10.1038/s42003-026-10840-6 (PMC13558674; doi:10.1038/s42003-026-10840-6)
Supplement: Supplementary file 4 [file 42003_2026_10840_MOESM6_ESM.pdf]

nature portfolio

Corresponding author(s): Jinlong Shi
Last updated by author(s): Feb 6, 2026

Reporting Summary

Nature Portfolio wishes to improve the reproducibility of the work that we publish. This form provides structure for consistency and transparency in reporting. For further information on Nature Portfolio policies, see our Editorial Policies and the Editorial Policy Checklist.

Statistics

For all statistical analyses, confirm that the following items are present in the figure legend, table legend, main text, or Methods section.

n/a	Confirmed	
☐	☒	The exact sample size (*n*) for each experimental group/condition, given as a discrete number and unit of measurement
☐	☒	A statement on whether measurements were taken from distinct samples or whether the same sample was measured repeatedly
☐	☒	The statistical test(s) used AND whether they are one- or two-sided<br>*Only common tests should be described solely by name; describe more complex techniques in the Methods section.*
☐	☒	A description of all covariates tested
☒	☐	A description of any assumptions or corrections, such as tests of normality and adjustment for multiple comparisons
☐	☒	A full description of the statistical parameters including central tendency (e.g. means) or other basic estimates (e.g. regression coefficient) AND variation (e.g. standard deviation) or associated estimates of uncertainty (e.g. confidence intervals)
☐	☒	For null hypothesis testing, the test statistic (e.g. *F*, *t*, *r*) with confidence intervals, effect sizes, degrees of freedom and *P* value noted<br>*Give P values as exact values whenever suitable.*
☒	☐	For Bayesian analysis, information on the choice of priors and Markov chain Monte Carlo settings
☒	☐	For hierarchical and complex designs, identification of the appropriate level for tests and full reporting of outcomes
☒	☐	Estimates of effect sizes (e.g. Cohen's *d*, Pearson's *r*), indicating how they were calculated

Our web collection on statistics for biologists contains articles on many of the points above.

Software and code

Policy information about availability of computer code

Data collection

Description of data collection and software used is available in Methods section. In this study, 225 individuals were recruited at the General Hospital.

Blood trait data: Routine blood tests were performed using the Mindray Auto Hematology Analyzer (BC-760), blood biochemical indicators were measured with the Mindray Automatic Biochemistry Analyzer (BS2000), and thyroid function was assessed using the Roche cobas 6000 Analyzer Series. All those tests were carried out on the same day of sampling.

Whole Genome Sequencing: DNA was extracted from peripheral blood using the TIANamp Blood DNA Kit, and WGS with a target coverage of 30× was performed on the DNBSEQ-T7 platform following MGI-provided protocols, with standard library preparation conducted in Shenzhen.

Transcriptome sequencing: RNA collected from samples in PAXgene tubes was isolated using the RNAprep Pure Hi-Blood Kit, TIANGEN, following the manufacturer's protocol. rRNA was removed using the MGIEasy RNA Directional Library Preparation Kit and a single- stranded cDNA library was prepared with the MGIEasy Circularization Kit. Whole-transcriptome sequencing was performed on the MGISEQ-2000 platform, with each sample sequenced to a depth of 20G bases to ensure data quality. Quality control of the raw Fastq files for both genomic and transcriptomic data was performed using SOAPnuke (v1.5.6).

Data analysis

Description of data analysis is available in Methods section. The code is available at https://gitee.com/jinlongshi/eQTL_han_tibetan.git.

Software	Version	LINK
BWA	0.7.17	https://github.com/lh3/bwa
VCFtools	0.1.13	https://vcftools.github.io/

GCTA	1.94.1	https://yanglab.westlake.edu.cn/software/gcta/#Overview
Hisat2	2.2.0	http://daehwankimlab.github.io/hisat2/
featureCounts	2.0.2	https://subread.sourceforge.net/featureCounts.html
QTLtools	1.3.1	https://qtltools.github.io/qtltools/
Decon-eQTL	1.4	https://github.com/molgenis/systemsgenetics/tree/master/Decon2
plink	1.9	https://www.cog-genomics.org/plink2/
Cytoscape	3.10.2	https://cytoscape.org/
S-LDSC	1.0.1	https://github.com/bulik/ldsc
SMR	1.3.1	https://yanglab.westlake.edu.cn/software/smr/
coloc	5.2.3	https://github.com/chr1swallace/coloc
FUSION	3	http://gusevlab.org/projects/fusion/
FOCUS	0.7	https://github.com/bogdanlab/focus
clusterProfiler	4.6.0	https://github.com/YuLab-SMU/clusterProfiler

For manuscripts utilizing custom algorithms or software that are central to the research but not yet described in published literature, software must be made available to editors and reviewers. We strongly encourage code deposition in a community repository (e.g. GitHub). See the Nature Portfolio guidelines for submitting code & software for further information.

Data

Policy information about availability of data

All manuscripts must include a data availability statement. This statement should provide the following information, where applicable:

- Accession codes, unique identifiers, or web links for publicly available datasets
- A description of any restrictions on data availability
- For clinical datasets or third party data, please ensure that the statement adheres to our policy

All datasets are available on request or through online repositories. The raw Genotype data and RNA-seq data can be requested at https://ngdc.cncb.ac.cn/ (accession number: HRA009343). The Yang-GWAS summary statistics is available for downloaded from cnsgenomics.com/data/yang_et_al_2017_pnas.html. East Asian high-altitude adaptation phenotype GWAS summary statistics is available at BBJ (https://pheweb.jp/). The statistics of eQTLs in GTEx v8 is available at https://www.gtexportal.org/home/downloads/adult-gtex/qtl. The statistics of eQTLs in eQTLGen is available at https://www.eqtlgen.org/phase1.html. Other data sources used in this study include 15 chromatin states from REMC (https://egg2.wustl.edu/roadmap/data/byFileType/chromhmmSegmentations/ChmmModels/coreMarks/joint Model/final/); the histone modifications Chip-seq data for K562 cell line (https://egg2.wustl.edu/roadmap/data/byFileType/alignments/consolidated/); the DNA-binding protein binding sites from ENCODE DNase-seq (https://www.encodeproject.org/annotations/ENCSR695MET/); 1000genome (http://ftp.1000genomes.ebi.ac.uk/vol1/ftp/data_collections/1000_genomes_project/release/20181203_biallelic_SNV/); ihypoxia annotation (http://ihypoxia.omicsbio.info/index.php).

Research involving human participants, their data, or biological material

Policy information about studies with human participants or human data. See also policy information about sex, gender (identity/presentation), and sexual orientation and race, ethnicity and racism.

Reporting on sex and gender	There are 26 females and 199 males in our cohort.
Reporting on race, ethnicity, or other socially relevant groupings	All individuals in our study were East Asian.
Population characteristics	A total of 225 healthy individuals who did not report any treatment history or not currently diagnosed as chronic diseases or cancer were enrolled in this study (164 Han and 61 Tibetan, 26 are females and 199 are males) with age varying from 17 to 72 years old.
Recruitment	225 individuals were recruited at the Qinghai-Tibet Plateau by our research group. 164 Han and 61 Tibetan were included in this study.
Ethics oversight	This study was approved by the Ethics Review Board of the General Hospital (S2016-057-02)

Note that full information on the approval of the study protocol must also be provided in the manuscript.

Field-specific reporting

Please select the one below that is the best fit for your research. If you are not sure, read the appropriate sections before making your selection.

☒ Life sciences ☐ Behavioural & social sciences ☐ Ecological, evolutionary & environmental sciences

For a reference copy of the document with all sections, see nature.com/documents/nr-reporting-summary-flat.pdf

Life sciences study design

All studies must disclose on these points even when the disclosure is negative.

Sample size	The sample size used in this study were not predeterminded. The number of individuals is sufficient to detect high-confidence eQTLs for Han and Tibetan.
Data exclusions	No data were excluded.
Replication	The raw data had been deposited in public repositories. The codes of data analysis are publicly available at https://gitee.com/jinlongshi/eQTL_han_tibetan.git. All these ensure the reproducibility of the experimental findings.
Randomization	All the samples were selected randomly.
Blinding	Blinding is not relevant to this study.

Behavioural & social sciences study design

All studies must disclose on these points even when the disclosure is negative.

Study description	
Research sample	
Sampling strategy	
Data collection	
Timing	
Data exclusions	
Non-participation	
Randomization	

Ecological, evolutionary & environmental sciences study design

All studies must disclose on these points even when the disclosure is negative.

Study description	
Research sample	
Sampling strategy	
Data collection	
Timing and spatial scale	
Data exclusions	
Reproducibility	
Randomization	
Blinding	

Did the study involve field work? ☐ Yes ☐ No

Field work, collection and transport

Field conditions	

Field conditions	
Location	
Access & import/export	
Disturbance	

Reporting for specific materials, systems and methods

We require information from authors about some types of materials, experimental systems and methods used in many studies. Here, indicate whether each material, system or method listed is relevant to your study. If you are not sure if a list item applies to your research, read the appropriate section before selecting a response.

Materials & experimental systems

n/a	Involved in the study
☐	☒ Antibodies
☐	☒ Eukaryotic cell lines
☒	☐ Palaeontology and archaeology
☒	☐ Animals and other organisms
☒	☐ Clinical data
☒	☐ Dual use research of concern
☒	☐ Plants

Methods

n/a	Involved in the study
☒	☐ ChIP-seq
☐	☒ Flow cytometry
☒	☐ MRI-based neuroimaging

Antibodies

Antibodies used	anti-Exo84 antibody (H-1) (sc-515532, Santa Cruz); APC anti-human CD71 Antibody (334180, Biolegend); PE anti-human CD235a (Glycophorin) Antibody (349106, Biolegend).
Validation	Validation detail was showed in https://www.scbt.com/p/exo84-antibody-h-1. APC anti-human CD71 and PE anti-human CD235a (Glycophorin) antibodies were validated by Biolegend.

Eukaryotic cell lines

Policy information about cell lines and Sex and Gender in Research

Cell line source(s)	ATCC
Authentication	The human leukemic cell line K562 cells
Mycoplasma contamination	All cell lines used were negative for mycoplasma contamination.
Commonly misidentified lines (See ICLAC register)	K562

Palaeontology and Archaeology

Specimen provenance	
Specimen deposition	
Dating methods	

☐ Tick this box to confirm that the raw and calibrated dates are available in the paper or in Supplementary Information.

Ethics oversight	

Note that full information on the approval of the study protocol must also be provided in the manuscript.

Animals and other research organisms

Policy information about studies involving animals; ARRIVE guidelines recommended for reporting animal research, and Sex and Gender in Research

Laboratory animals

Wild animals

Reporting on sex

Field-collected samples

Ethics oversight

Note that full information on the approval of the study protocol must also be provided in the manuscript.

Clinical data

Policy information about clinical studies

All manuscripts should comply with the ICMJE guidelines for publication of clinical research and a completed CONSORT checklist must be included with all submissions.

Clinical trial registration

Study protocol

Data collection

Outcomes

Dual use research of concern

Policy information about dual use research of concern

Hazards

Could the accidental, deliberate or reckless misuse of agents or technologies generated in the work, or the application of information presented in the manuscript, pose a threat to:

No	Yes	
☐	☐	Public health
☐	☐	National security
☐	☐	Crops and/or livestock
☐	☐	Ecosystems
☐	☐	Any other significant area

Experiments of concern

Does the work involve any of these experiments of concern:

No	Yes	
☐	☐	Demonstrate how to render a vaccine ineffective
☐	☐	Confer resistance to therapeutically useful antibiotics or antiviral agents
☐	☐	Enhance the virulence of a pathogen or render a nonpathogen virulent
☐	☐	Increase transmissibility of a pathogen
☐	☐	Alter the host range of a pathogen
☐	☐	Enable evasion of diagnostic/detection modalities
☐	☐	Enable the weaponization of a biological agent or toxin
☐	☐	Any other potentially harmful combination of experiments and agents

Plants

Seed stocks

Novel plant genotypes

Authentication

ChIP-seq

Data deposition

☐ Confirm that both raw and final processed data have been deposited in a public database such as GEO.

☐ Confirm that you have deposited or provided access to graph files (e.g. BED files) for the called peaks.

Data access links
May remain private before publication.

Files in database submission

Genome browser session
(e.g. UCSC)

Methodology

Replicates

Sequencing depth

Antibodies

Peak calling parameters

Data quality

Software

Flow Cytometry

Plots

Confirm that:

☒ The axis labels state the marker and fluorochrome used (e.g. CD4-FITC).

☒ The axis scales are clearly visible. Include numbers along axes only for bottom left plot of group (a 'group' is an analysis of identical markers).

☒ All plots are contour plots with outliers or pseudocolor plots.

☒ A numerical value for number of cells or percentage (with statistics) is provided.

Methodology

Sample preparation	All cell were mixed and filtered before use. For staining, cells were washed with PBS before staning with antibody. After incubation, cells were washed twice and resuspended in PBS. All flow cytometery was performed on a BD FACS CatoII flow cytometer (BD company).
Instrument	BD FACS CatoII flow cytometer
Software	FlowJo
Cell population abundance	10,000 single cell were collected for each sample.

Gating strategy | Cells were firstly gated to cell debris (FSC-A/SSC-A), and single cells (FSC-A/FSC-H). Cells were then gated for either (FITC), PE or APC, depending on our purpose of the analysis.

☒ Tick this box to confirm that a figure exemplifying the gating strategy is provided in the Supplementary Information.

Magnetic resonance imaging

Experimental design

Design type

Design specifications

Behavioral performance measures

Acquisition

Imaging type(s)

Field strength

Sequence & imaging parameters

Area of acquisition

Diffusion MRI ☐ Used ☐ Not used

Preprocessing

Preprocessing software

Normalization

Normalization template

Noise and artifact removal

Volume censoring

Statistical modeling & inference

Model type and settings

Effect(s) tested

Specify type of analysis: ☐ Whole brain ☐ ROI-based ☐ Both

Statistic type for inference

(See Eklund et al. 2016)

Correction

Models & analysis

n/a	Involved in the study
☐	☐ Functional and/or effective connectivity
☐	☐ Graph analysis
☐	☐ Multivariate modeling or predictive analysis

Functional and/or effective connectivity

Graph analysis

Multivariate modeling and predictive analysis
